# Thioether and Ether Furofuran Lignans: Semisynthesis, Reaction Mechanism, and Inhibitory Effect against α-Glucosidase and Free Radicals

**DOI:** 10.3390/molecules27249001

**Published:** 2022-12-17

**Authors:** Wisuttaya Worawalai, Nantaporn Surachaitanawat, Phonpimon Khongchai, Viwat Vchirawongkwin, Thammarat Aree, Preecha Phuwapraisirisan

**Affiliations:** Department of Chemistry, Faculty of Science, Chulalongkorn University, Bangkok 10330, Thailand

**Keywords:** glucosidase, antioxidant, furofuran lignan, thiols, alcohols

## Abstract

The transformation of sesame lignans is interesting because the derived products possess enhanced bioactivity and a wide range of potential applications. In this study, the semisynthesis of 28 furofuran lignans using samin (**5**) as the starting material is described. Our methodology involved the protonation of samin (**5**) to generate an oxocarbenium ion followed by the attack from two different nucleophiles, namely, thiols (RSH) and alcohols (ROH). The highly diastereoselective thioether and ether furofuran lignans were obtained, and their configurations were confirmed by 2D NMR and X-ray crystallography. The mechanism underlying the reaction was studied by monitoring ^1^H NMR and computational calculations, that is, the diastereomeric α- and β-products were equally formed through the S_N_1-like mechanism, while the β-product was gradually transformed via an S_N_2-like mechanism to the α-congener in the late step. Upon evaluation of the inhibitory effect of the synthesized lignans against α-glucosidases and free radicals, the lignans **7f** and **7o** of the phenolic hydroxyl group were the most potent inhibitors. Additionally, the mechanisms underlying the α-glucosidase inhibition of **7f** and **7o** were verified to be of a mixed manner and noncompetitive inhibition, respectively. The results indicated that both **7f** and **7o** possessed promising antidiabetic activity, while simultaneously inhibiting α-glucosidases and free radicals.

## 1. Introduction

**Figure 1:**
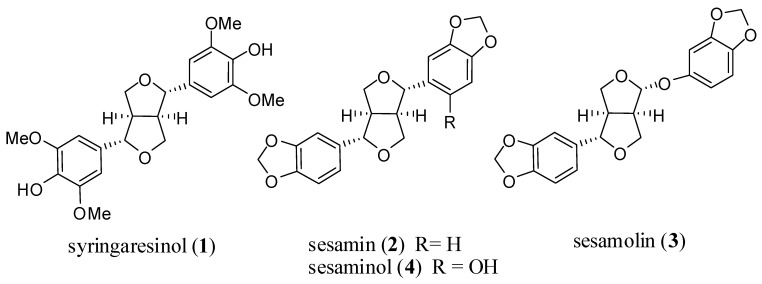
Structures of bioactive furofuran lignans.

Furofuran lignan is an important group characterized by the presence of a 2,6-diaryl-3,7-dioxabicyclo[3.3.0]octane skeleton. Numerous furofuran lignans have been isolated from various plants, and their biological activities have been evaluated; for example, antitumoral activity of syringaresinol (**1**) [1], cholesterol lowering effect of sesamin (**2**) in rat serum and liver [2], and antioxidant of sesamolin (**3**) [3] (Figure 1). Owing to the broad range of activities and structural diversity of furofuran lignans, the development of synthetic methodologies has been reported to improve their bioactivities [4,5,6]. However, no investigation into the structure activity relationship (SAR) has been reported, due to the lack of a practical synthetic method to produce diverse furofuran lignans.

We report a new synthetic approach to easily produce a wide series of furofuran lignans. Our approach is based on the semi-synthesis of sesaminol (**4**) using naturally available sesamolin (**3**) as a starting material in the presence of acidic resin as a catalyst [7]. The success of transforming sesamolin (**3**) into samin (**5**), a versatile lignan building block, enabled us to synthesize a variety of furofuran lignans. We initially applied phenolics and flavonoids as carbon nucleophiles to couple with samin (**5**), thereby producing various pairs of epimeric products (α- and β-forms) in fair to good yields [8,9]. Moreover, we also synthesized a series of furofuran lignans with catechol moieties via oxidative the cleavage of methylenedioxy moiety [10]. As a consequence, up to 50 furofuran lignans could be synthesized and evaluated for their inhibitory effect against α-glucosidase and free radicals, thus elucidating the critical role of the catechol moiety in SAR. However, the carbon nucleophiles used in this reaction were in fact limited to certain phenolics and flavonoids containing more electron-donating groups.

**Scheme 1:**
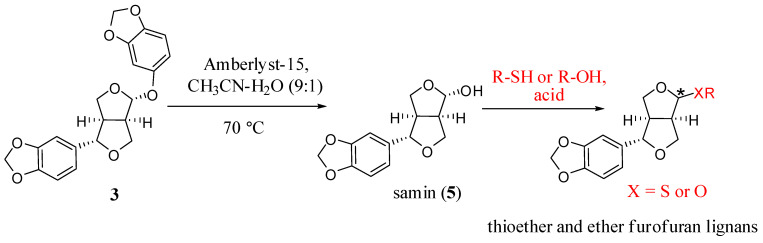
Synthetic strategy for thioether and ether furofuran lignans.

To expand the scope of the synthetic methodology, the synthesis of a new series of furofuran lignans using thiols and alcohols as sulfur and oxygen nucleophiles, respectively, was established (Scheme 1). Thiols and alcohols as stronger nucleophiles would promote product yield, and offer diverse furofuran lignans. In this project, the reaction mechanism and inhibitory effect against α-glucosidases and free radicals, and a kinetic study are also described. This investigation provides insights into furofuran lignans in the scope of the synthetic methodology and the reaction mechanism together with the structure activity relationship with regard to α-glucosidase and free radicals.

## 2. Results and Discussion

### 2.1. Chemistry

Prior to studying the scope of thioethers and ethers as nucleophiles, we screened a variety of acids using methanol as a model nucleophile in an acetonitrile solution of samin (**5**) (Table S1). Lewis acid (AlCl_3_ and BF_3_.Et_2_O) and Brønsted acid (HCl, CF_3_CO_2_H, and Amberlyst-15) efficiently promoted the nucleophilic substitution of samin (**5**) with methanol, thereby providing a single diastereomer (**2α**-**9a**) in a high yield (95–98%), while acetic acid (weak Brønsted acid) failed to trigger the reaction. Although several Lewis and Brønsted acids can be applied in this reaction, the heterogeneous Amberlyst-15 was used for further investigation because no workup step is required when using this.

### 2.2. Synthesis and Characterization of Thioether Furofuran Lignans

With optimized reaction conditions in hand, nucleophilic substitutions of samin (**5**) with thiols (sulfur nucleophiles) were first accomplished (Table 1). The thiols used in this work can be classified into two categories, aliphatic and aromatic thiols. 1-Octanethiol (**6a**) served as a good nucleophile and reacted with samin to generate the product **2α-7a** in a quantitative isolated yield (Table 1, entry 1). The reaction between samin (**5**) and 6-mercapto-1-hexanol (**6b**) produced thioether furofuran lignan **2α-7b** (Table 1, entry 1) without the detection of ether furofuran lignan, although **6b** contained both sulfur and oxygen nucleophiles. Cyclohexanethiol (**6c**) also smoothly reacted with samin (**5**) to furnish the product **2α-7c** with a 95% yield (Table 1, entry 3). Nucleophilic substitution of samin (**5**) and thiols also produced thioether furofuran lignans in high yields (**2α-7d** (93%) and **2α-7e** (96%), although bulky nucleophiles such as **6d** and **6e** were applied (Table 1, entries 4–5).

Electronically diverse substituted thiophenols also served as nucleophiles to generate the S-derived products without the observation of C-derived products. p-substituted aromatic thiols **6f**, **6g**, and **6h** smoothly reacted, thereby producing the desired products **2α-7f**, **2α-7g**, and **2α-7h** in high yield (Table 1, entries 6-8). However, the product yield significantly decreased upon treatment of samin (**5**) with 4-nitrothiophenol (**6i**) to provide **7i** at 83% yield (Table 1, entry 9) because of the lower nucleophilicity contributed by the nitro group. The steric hindrance of o-substituted aromatic thiols also caused a significantly lower yield of lignans. For example, the reaction between samin (**5**) and o-substituted aromatic thiol **6j** furnished a lower yield of lignan **2α-7j** (78%) compared to lignans produced using m- and p-substituted aromatic thiol **6k** and **6l** (85% and 90% yield) as nucleophiles. Some other lignan yields were **2α-7m** (87%), **2α-7n** (93%), and **2α-7o** (89%).

Generally, the reactions between samin (**5**) and thiols produced furofuran lignans in higher yield (78%-quant), and α-products were exclusively obtained as an isolated yield. Theoretically, the reaction proceeds through an oxocarbenium ion and generates a mixture of α- and β-products with a diastereomeric ratio of 1:1 [11]. In our previous studies, we obtained isolated α- and β-products in the ratios of 1:1 to 3:1 from the reactions between samin (**5**) and a series of phenolics and flavonoids as carbon nucleophiles. Therefore, the unexpected results prompted us to determine a diastereomeric ratio of α- and β-thioether furofuran lignans using an ^1^H NMR analysis of the crude reaction mixture (Table 1). Apparently, the reactions between the samin (**5**) and thiols produced α- and β-products in the ratio of 5:1 to 8:1, while the steric hindrance resulted in a higher diastereomeric ratio of 12:1. This led to the inference that the alpha-product was obtained as an isolated yield.

**Figure 2:**
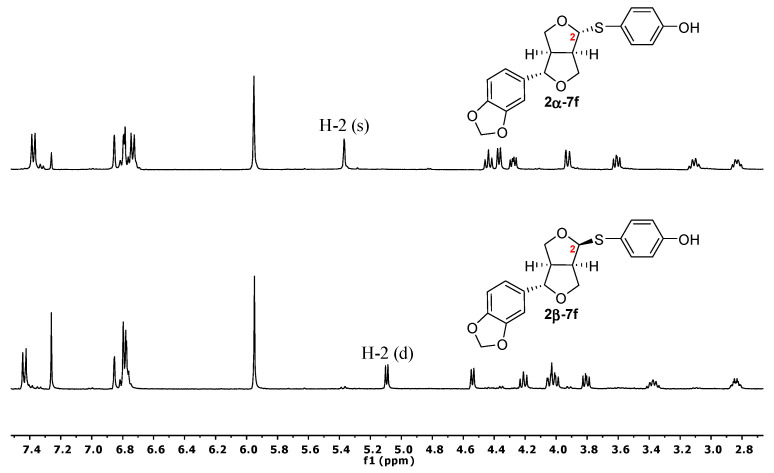
^1^H NMR spectra of **2α-7f** (**top**) and **2β-7f** (**bottom**).

**Figure 3:**
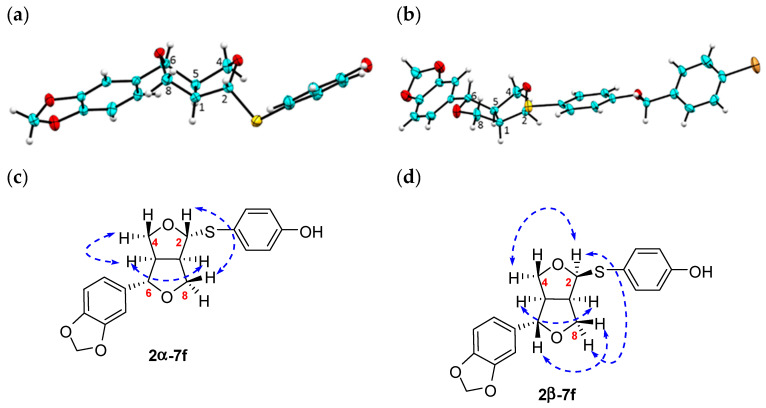
ORTEP plots (thermal ellipsoids at 20% probability level) of (**a**) **2*α*-7f** and (**b**) **2*β*-7fBr** (color codes: C = cyan, S = yellow, Br = orange, O = red, H = white) and select NOESY correlations of (**c**) **2*α*-7f** and (**d**) **2*β*-7f**.

To fully characterize the structures of the thioether furofuran lignans and assign the configuration of the α- and β-products, we scaled up the reaction between samin (**5**) and thiol **6f** to afford an adequate amount of diastereomeric products in a pure form. The structures of **2α-7f** and **2β-7f** were verified by MS and 2D NMR (see supporting information). The configuration of the α- and β-products was assigned by coupling constant (Figure 2) and NOESY (Figure 3) data analysis. A singlet signal of H-2 appearing at δ_H_ 5.37 ppm (Figure 2) was characteristic of the α-product, which was contributed by the dihedral angle (θ) of 90° between H-1 and H-2. On the other hand, the β-product showed a doublet signal of H-2 at δ_H_ 5.10 ppm together with a typical coupling constant of 6.1 Hz (Figure 2). The characteristic splitting pattern of H-2 would be useful to address the configuration of the α- and β-products synthesized from samin (**5**) and thiols.

Furthermore, the structures of the thioether furofuran lignans were unequivocally verified by X-ray crystallography using a pair of diastereomers **7f**. Prior to performing the X-ray analysis, modification of **2β-7f** was required to convert it from a greasy appearance to a crystalline solid by reacting it with 4-bromobenzyl bromide (see Experimental section for details). The X-ray crystal structures of **2α-7f** and **2β-7fBr** (Figure 3) clearly showed the cis fusion of the two furan moieties. The five-membered rings adopted an envelope conformation with the oxygen O-3 and O-7 atoms as a flip. The substituents at C-2 and C-6 of furofuran moiety occupied a syn orientation for **2α-7f** (Figure 3a) while an anti orientation was noticed for **2β-7fBr** (Figure 3b). Notably, the dihedral angle between H-1 and H-2 of **2α-7f** and **2β-7fBr** showed 96°and 28°, respectively. These observations were identical to those of ^1^H NMR results.

### 2.3. Synthesis and Characterization of Oether Furofuran Lignans

The success of synthesizing thioether furofuran lignans led to the subsequent examination of the nucleophilic substitution of samin with alcohols (Table 2). Alcohols used in this experiment can be divided into three categories—primary, secondary, and tertiary alcohols. The reaction between octanol (**8b**), the primary alcohol, and samin (**5**) generated **2α-9b** in a quantitative yield. The lignan product **2α-9c** could be successfully synthesized in a quantitative yield (Table 2, entry 2) using longer aliphatic primary alcohol, decanol (**8d**), as a nucleophile. Aliphatic diols, 1,6-hexanediol (**8d**) and 1,10-decanediol (**8e**), also reacted with samin to furnish the products **2α-9d** and **2α-9e** in quantitative yields (Table 2, entries 3–4) without the detection of the dimeric products. β–Citronellol (**8f**), a natural aliphatic monoterpenoid, also reacted smoothly with samin to generate the target product (**2α-9f**) at 90% yield (Table 2, entry 5). Allylic alcohols, geraniol (**8g**) and trans,trans-farnesol (**8h**), also worked well to generate the products **2á-9g** and **2á-9h** in 89% and 84% yields, respectively (Table 2, entries 6-7). Remarkably, the reaction of samin with piperonyl alcohol (**8i**) produced the target product (**2α-9i**) at 45% yield (Table 2, entry 8) along with the undesired side product (self-coupling of piperonyl alcohol) in nearly 40% yield. The secondary alcohols, cyclohexanol (**8j**), α-terpeneol (**8k**), and borneol (**8l**), reacted smoothly to produce the desired products **2α-9j**, **2α-9k**, and **2α-9l** in 93%, 89%, and 90% yields, respectively (Table 2, entries 9–11). Furthermore, an extremely sterically hindered alcohol **8m** reacted smoothly with samin (**5**) to furnish the target product (**2α-9m**) at 81% yield (Table 2, entry 12).

**Figure 4:**
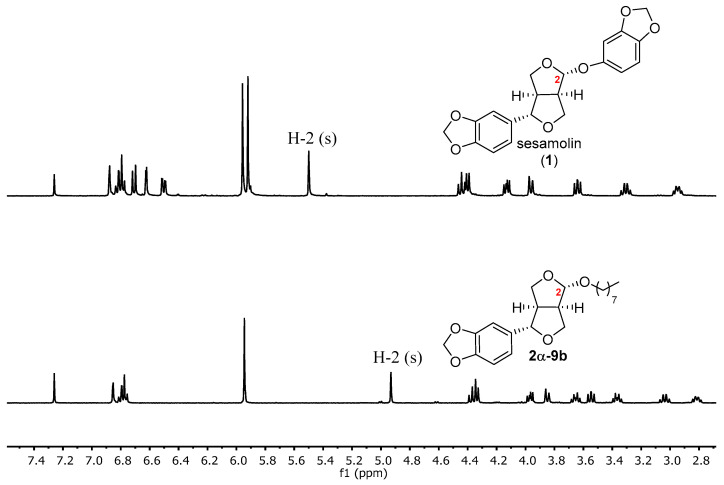
^1^H NMR spectra of sesamolin (**1**) (**top**) and **2α-9b** (**bottom**).

The structures of all ether furofuran lignans (**2α-9b**–**2α-9m**) were characterized by ^1^H NMR, ^13^C NMR, and HRESIMS. The 2α stereochemistry was ascertained by ^1^H NMR data when compared to that of sesamolin. A singlet signal appearing at ä_H_ 4.93 ppm was indicative of β-orientation of H-2 of **9b,** which was similar to that of sesamolin (Figure 4). It should be noted that all the above-mentioned nucleophilic substitutions of samin with alcohols as nucleophiles (Table 2) provided α–products in high yields and excellent diastereoselectivity (>25:1). The unexpected results of this reaction and its mechanism are clarified in the next section.

### 2.4. Investigation of Reaction Mechanism of Ether Furofuran Lignan Formation

**Figure 5:**
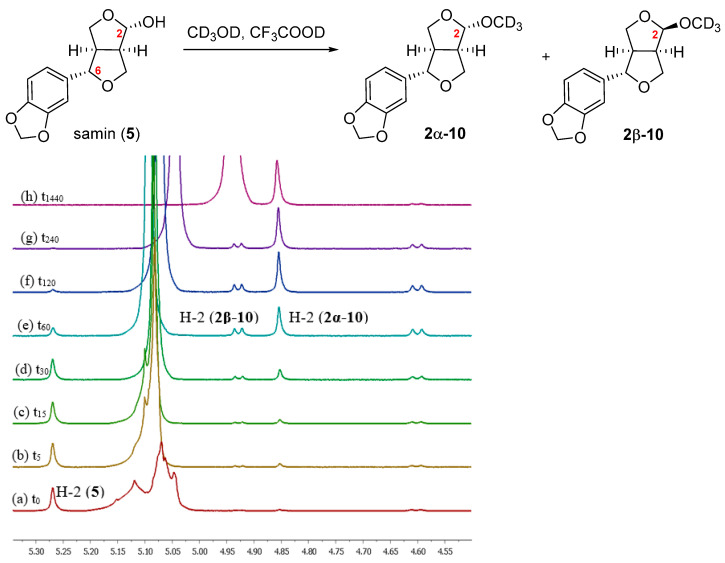
Overlaid ^1^H NMR spectra of the nucleophilic substitution reaction of samin for different times.

**Figure 6:**
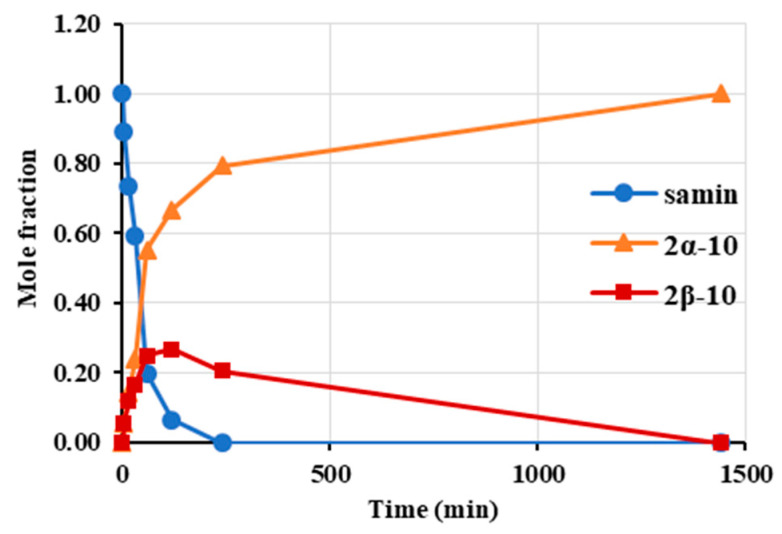
Time course of the evolution of samin (**5**), **2α-10**, and **2β-10** in mole fraction monitored using ^1^H NMR spectroscopy (400 MHz).

To investigate how an α-product exclusively formed during the nucleophilic substitution of samin (**5**) with alcohols (**8b**–**8m**), ^1^H NMR was used to follow the reaction progress. The model reaction comprising of samin (**5**), methanol-d_4_ as a nucleophile and solvent, along with 10 mol% of TFA-d_1_ as an acid catalyst, was carried out in an NMR tube. Figure 5 shows an overlaid ^1^H NMR spectra of the model reaction monitored at eight different times (t = 0, 5, 15, 30, 60, 120, 240, and 1440 min). The occurrence of **2α-10** (α-product) and **2β-10** (β-product) was observed by the presence of H-2 signals at ^TM^_H_ 4.85 (s) and 4.93 (d, *J* = 5.5 Hz), respectively, while the depletion of samin (**5**) was noted by the reduction in signal intensity at ^TM^_H_ 5.27 (s). This observation preliminarily suggested that the substitution of samin (**5**) by alcohol potentially proceeded through the formation of an oxocarbenium ion followed by a nucleophilic attack. To probe the reaction progress after α- and β-products were generated, the time-course plot of the evolution of the starting material and products was constructed (Figure 6).

**Scheme 2:**
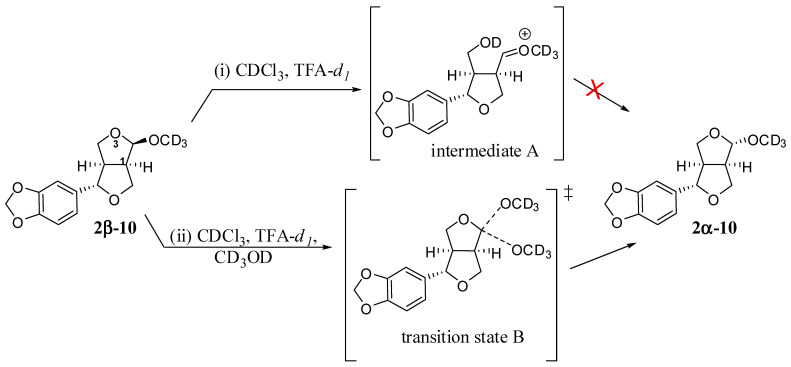
Proposed transformation of **2β-10** to **2α-10** under different conditions.

To probe how the β–product transformed to an α-product after samin (**5**) was completely consumed, we set two independent reactions of **2β-10** with and without methanol-d_4_ as the nucleophile in the presence of TFA-d_1_ as the acid catalyst and CDCl_3_ as the solvent (Scheme 2). Previously, the S_N_1-like mechanism (Route i) had been proposed for the epimerization of certain furofuran lignans [4,12], which involved the protonation of O-3 to generate intermediate A followed by ring closure to yield an α-product. Alternatively, the S_N_2-like mechanism (Route ii) possibly took place via the displacement of –OCD_3_ by the introduction of CD_3_OD through the transition state B. 

**Figure 7:**
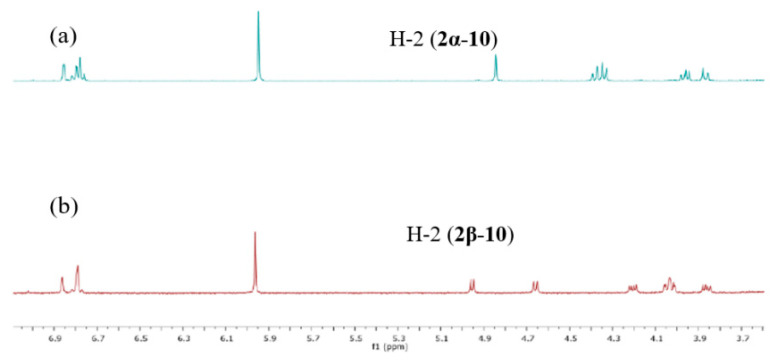
Overlaid ^1^H NMR spectra of (**a**) **2α-10** occurred from the starting **2β-10** treated with CD_3_OD and TFA-*d*_1_ for 10 min and (**b**) the starting **2β-10**.

The results of this experiment are presented in Figure 7. The ^1^H NMR spectra clearly showed that 10 min after the addition of methanol-d_4_ into the reaction, **2α-10** readily formed (Figure 7a). Additionally, we also performed the reaction of **2β-10** with methanol-d_4_ as a nucleophile in CDCl_3_ and the results displayed that it had no product **2α-10** produced in this condition (data not shown). Hence, we hypothesized that the transformation of a β–product to an α–product using alcohol nucleophile could occur through an S_N_2-like mechanism.

**Figure 8:**
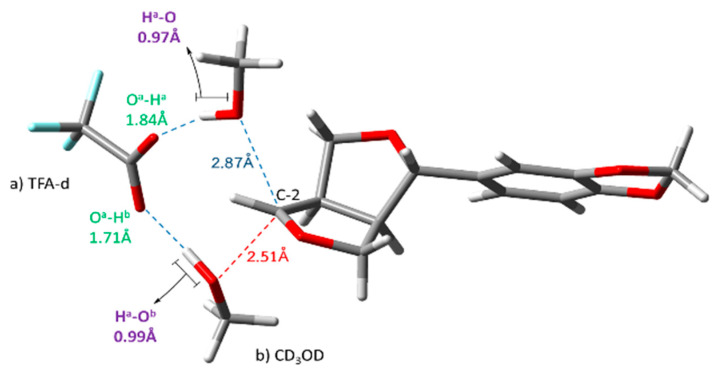
Transition state for the epimerization reaction of **2β-10**.

Our finding is strikingly different from that of Johansson [12], who proposed that the epimerization of furanosides proceeded through an S_N_1-like mechanism without experimental data to support this claim. We also confirmed a transformational possibility of **2β-10** by bond distance observation of the structure at the transition state, using the PCM/B3LYP/6-31+G(d,p) method to optimize the transition state structure (Figure 8). As the nucleophile and leaving group were totally the same alcohols. The C-O (breaking bond) and C-O^b^ (forming bond) bond distances at the transition state were not significantly different. However, the slightly shorter new C-O^b^ (2.51 Å) bond had a higher preference than the C-O bond. The short H^a^-O bond (0.97 Å) also supported the fact that the –OCD_3_ of **2β-10** tends to leave after deuteration by TFA-d_1_. All of the illustrated evidence reasonably demonstrates the transformation of **2β-10** to **2α-10** occurring through a S_N_2-like transition state [13].

**Scheme 3:**
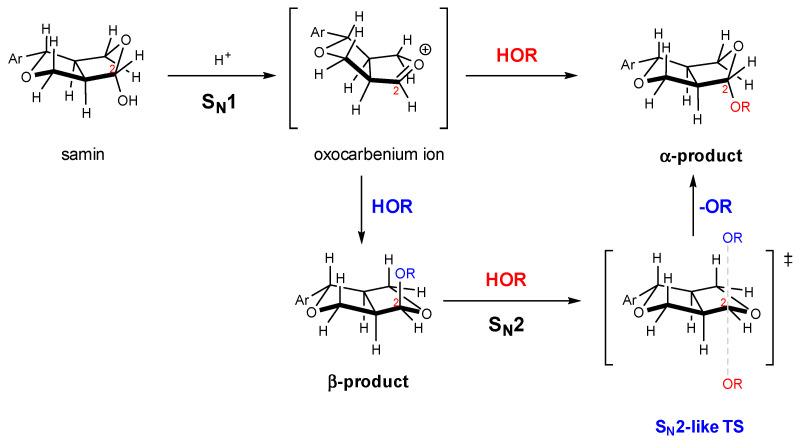
Proposed mechanism of ether furofuran lignan formation.

According to the above results, the mechanism of ether furofuran lignans formation could be deduced as the oxocarbenium ion being first generated by the protonation of the hemiacetal center of samin through an S_N_1-like mechanism to furnish both α– and β–products in an equal ratio. During the reaction progress, the β–product transformed to an α–product via the S_N_2-like transition state contributed by an acid catalyst (Scheme 3).

### 2.5. Evaluation of α-Glucosidase Inhibition and Antioxidant Activity

All furofuran lignans were evaluated for rat intestinal α-glucosidase inhibition as well as antioxidation against DPPH and ABTS radicals (Table 3). The thioether furofuran lignans **2α-7f**, **2β-7f**, and **2α-7o** showed comparable inhibitory effects against α-glucosidases (11.8–16.2 mM) and free radicals (0.20–0.93 mM) while other thioether furofuran lignans and ether furofuran lignans were not active. Remarkably, the bioactive lignans comprised free phenolic moiety that may be involved in exerting an inhibitory effect. The results in this work are consistent with previous observations that the number of free phenolic groups is critical for exhibiting the inhibition [8,9,10]. Additionally, the presence of two tert-butyl groups in **2α-7o** as bulky groups does not alter the inhibitory effects, therefore confirming that free phenolic moiety principally plays an important role in the inhibition.

### 2.6. Enzyme Kinetic Study

**Figure 9:**
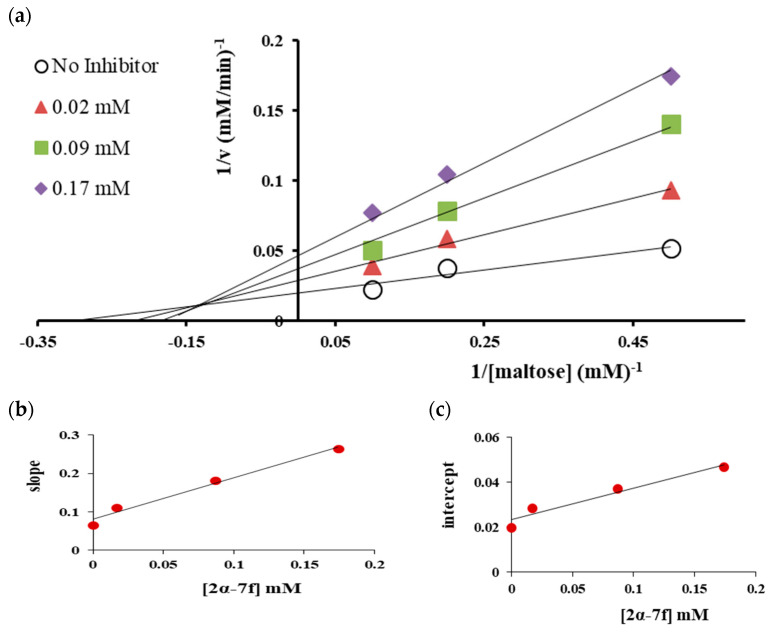
Lineweaver–Burk plots for inhibitory activity of **2α-7f** against rat intestinal maltase (**a**). Secondary replots of (**b**) slope vs. [I] and (**c**) intercept vs. [I] from a primary Lineweaver–Burk plot for the determination of *K*_i_ and *K’*_i_, respectively.

**Figure 10:**
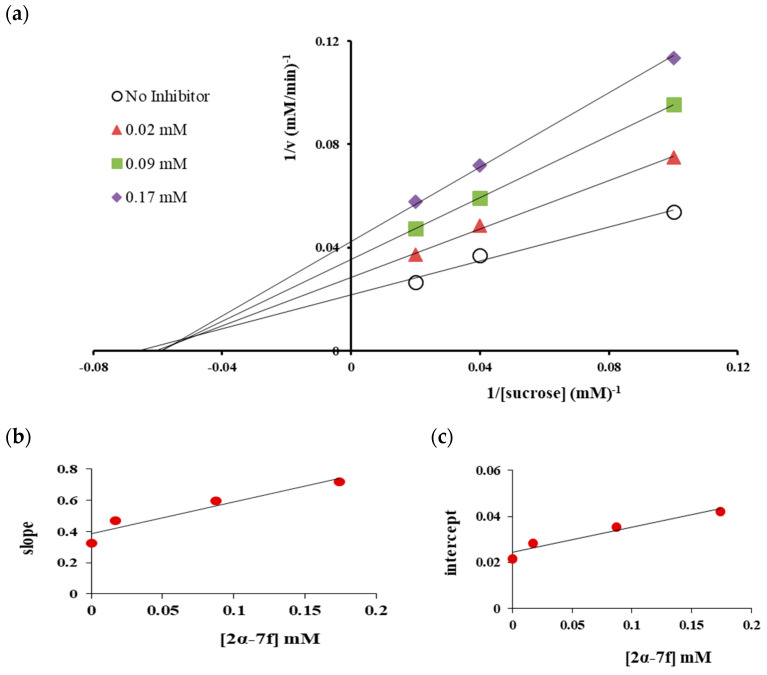
Lineweaver–Burk plots for inhibitory activity of **2α-7f** against rat intestinal sucrase (**a**). Secondary replots of (**b**) slope vs. [I] and (**c**) intercept vs. [I] from a primary Lineweaver–Burk plot for the determination of K_i_ and K’_i_, respectively.

**Scheme 4:**
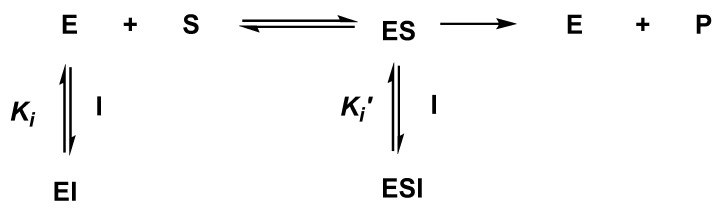
Putative inhibitory mechanism of **2α-7f** and **2α-7o** against rat intestinal α-glucosidases. E, S, I, and P represent enzyme, substrates (maltose and sucrose), inhibitors (**2α-7f** and **2α-7o**), and glucose, respectively.

In order to gain further insight into how these furofuran lignans interact with rat intestinal maltase and sucrase, the inhibition modes of **2α-7f** and **2α-7o**, the representative inhibitors, were analyzed by a kinetic study. In the case of **2α-7f**, the Lineweaver–Burk plot of maltase and sucrase (Figure 9 and Figure 10) showed a series of straight lines, all of which intersected in the second quadrant. Kinetic analysis subsequently showed that V_max_ decreased with elevated K_m_ in the presence of increasing concentrations of **2α-7f**. This behaviour suggests that **2α-7f** inhibited maltase and sucrase in a mixed-type manner comprising two different pathways: competitive and noncompetitive. The observed result was elaborated by the simultaneous formation of an enzyme-inhibitor (EI) and enzyme-substrate-inhibitor (ESI) complexes in competitive and noncompetitive manners, respectively (Scheme 4). We further investigated the pathway by which **2α-7f** was preferentially preceded through determining the dissociation constants of the EI (K_i_) and ESI (K′_i_) complexes (Table 4). Apparently, the secondary plots for maltase (Figure 9b,c) and sucrase (Figure 10b,c) show K_i_ values less than the K_i_′ values (Table 4), indicating that **2α-7f** was predominantly bound to maltase and sucrase (EI) rather than forming the ESI complex.

**Figure 11:**
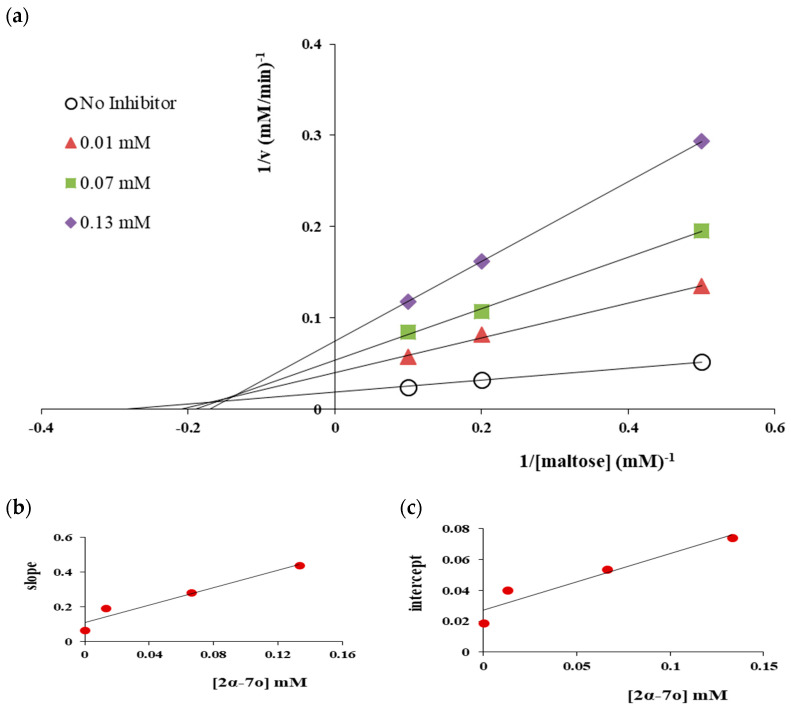
Lineweaver–Burk plots for inhibitory activity of **2α-7o** against rat intestinal maltase (**a**). Secondary replots of (**b**) slope vs. [I] and (**c**) intercept vs. [I] from a primary Lineweaver–Burk plot for the determination of *K*_i_ and *K’*_i_, respectively.

**Figure 12:**
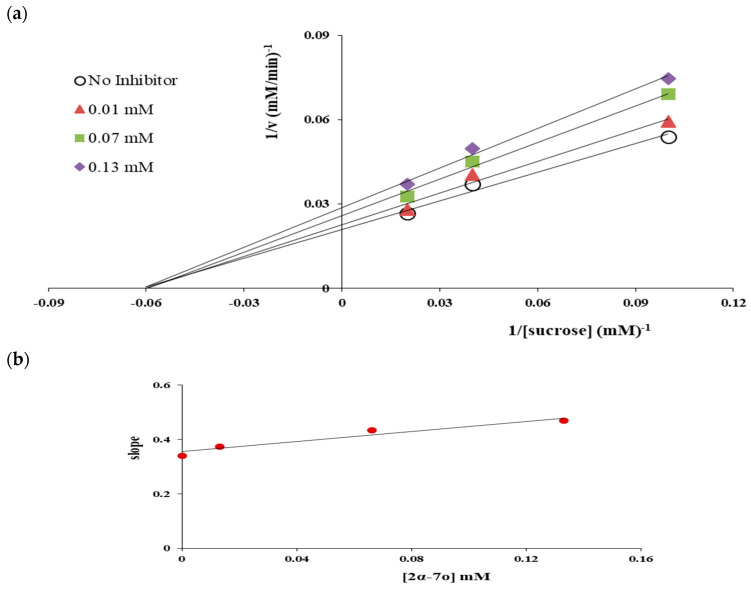
Lineweaver–Burk plots for inhibitory activity of **2α-7o** against rat intestinal sucrase (**a**). Secondary replots of slope vs. [I] (**b**) from a primary Lineweaver–Burk plot for the determination of K_i_.

For **2α-7o**, the inhibitory mechanisms against maltase and sucrase (Figure 11 and Figure 12) were also examined using the above methodology. The active **2α-7o** also inhibited maltase via a mixed-type inhibition (Figure 11). On the other hand, the Lineweaver–Burk plots of **2α-7o** against sucrase (Figure 12) showed a series of straight lines, all of which intersected on the *x*-axis. Kinetic analysis showed that V_max_ decreased with unchanged K_m_ in the presence of increasing concentrations of inhibitor. This behaviour suggested that **2α-7o** was a noncompetitive inhibitor against sucrase. The kinetic parameters of the active compounds are summarized in Table 4.

## 3. Materials and Methods

### 3.1. Chemicals

All moisture-sensitive reactions were carried out under a nitrogen atmosphere. All solvents were distilled prior to use. High resolution electrospray ionisation mass spectra (HRESIMS) were recorded with a Bruker microTof spectrometer (Billerica, MA, USA). ^1^H and ^13^C NMR spectra were recorded (CDCl_3_ and CD_3_OD as solvents) at 400 and 100 MHz, respectively, on a Varian Mercury^+^ 400 NMR and a Bruker (Avance) 400 NMR spectrometer. Chemical shifts are reported in ppm downfield from TMS or solvent residue. Thin layer chromatography (TLC) was performed on precoated Merck (Rahway, NJ, USA) silica gel 60 F_254_ plates (0.25 mm thick layer) and visualized using p-anisaldehyde reagent. Column chromatography was performed using Merck silica gel 60 (70-230 mesh) and Sephadex LH-20.

### 3.2. Synthetic Methods

#### 3.2.1. Preparation of Starting Material (Samin **5**)

A solution of sesamolin (**3**, 0.39 mmol) in a mixture of acetonitrile/H_2_O (9:1, 10 mL) was treated with acidic resin Amberlyst-15 (1 mg/0.005 mmol of **3**). After stirring at 70 °C for 8 h, the reaction mixture was evaporated to dryness and purified using a silica gel column (50%EtOAc-hexane) to produce samin **5** (88 mg, 90%) as a colorless oil.

#### 3.2.2. Synthesis of Thioether and Ether Furofuran Lignans

A solution of samin **5** (1 equiv.) in acetonitrile (1.0 mL/0.1 mmol of **5**) was treated with nucleophiles (3 equiv.), Amberlyst-15 (1 mg/0.005 mmol of **5**), and a 4 Å molecular sieve. After stirring at 70 °C for 8 h, the reaction mixture was evaporated to dryness and purified using a silica gel and sephadex LH-20 column to obtain furofuran lignans **2α-7a**–**2α-7o** and **2α-9a**–**2α-9m**.

The isolated lignan **2β-7f** used for structure characterization and bioactivity evaluation was obtained through multiple synthesis using the procedure described above. The combined crude reaction mixtures were further purified to obtain isolated **2β-7f** and **2α-7f** in pure form.

### 3.3. α-Glucosidase Inhibitory Activity

The α-glucosidase inhibitory activity against rat intestinal maltase and sucrase was determined according to our previous report [14]. The crude enzyme solution prepared from rat intestinal acetone powder (Sigma, St. Louis, MO, USA) was used as a source of maltase and sucrase. Rat intestinal acetone powder (1 g) was homogenized in 30 mL of 0.9% NaCl solution. After centrifugation (12,000× *g* × 30 min), the aliquot was subjected to an assay. The synthesized compounds (1 mg/mL in DMSO, 10 µL) were added with 30 µL of the 0.1 M phosphate buffer (pH 6.9), 20 µL of the substrate solution (maltose: 2 mM; sucrose: 20 mM) in 0.1 M phosphate buffer, 80 µL of glucose assay kit (SU-GLLQ2, Human), and 20 µL of the crude enzyme solution. The reaction mixture was then incubated at 37 °C for 10 min (for maltose) and 40 min (for sucrose). Enzymatic activity was quantified by measuring the absorbance of quinoneimine formed (500 nm) using the Bio-Rad 3550 microplate reader (Hercules, CA, USA). The percentage inhibition was calculated by [(A_0_ − A_1_)/A_0_] × 100, where A_1_ and A_0_ are the absorbance with and without the sample, respectively. The IC_50_ value was deduced from a plot of percentage inhibition versus sample concentration and acarbose was used as a positive control. The experiment was performed in triplicate.

### 3.4. Kinetic Study of α-Glucosidase Inhibition

For kinetic analysis of the active compound, α-glucosidases and active compounds were incubated with increasing concentrations of maltose (0.5–8 mM) and sucrose (5–80 mM). The type of inhibition was determined by the Lineweaver–Burk plot. For calculation of K_i_ and K_i_′ values, the slope and intercept from the Lineweaver–Burk plot were replotted vs. [I], which provided the secondary plot.

### 3.5. DPPH Radical Scavenging

The radical scavenging activity was validated using the DPPH colorimetric method. Briefly, the synthesized compounds (20 μL) were added to 0.1 mM methanolic solution of DPPH (100 μL). The mixture was kept in the dark at room temperature in an incubator shaker for 15 min. The absorbance of the resulting solution was measured at 517 nm with a 96-well microplate reader. The percentage inhibition was calculated by [(A_0_ − A_1_)/A_0_] × 100, where A_0_ is the absorbance without the sample, and A_1_ is the absorbance with the sample. The SC_50_ value was deduced from a plot of percentage inhibition versus sample concentration. Butylated hydroxytoluene (BHT) was used as the standard control and the experiment was performed in triplicate.

### 3.6. ABTS Radical Scavenging

The radical scavenging activity of synthesized compounds against ABTS•+ was carried out according to a procedure described previously [15]. Briefly, ABTS•+ radical cation was produced by mixing 10 mL of 7.4 mM ABTS with 0.5 mL of 2.6 mM potassium persulphate (K_2_S_2_O_8_) for 16 h in the dark at room temperature. Before use, the ABTS•+ solution was diluted with ethanol to an absorbance of 0.70 ± 0.02 at 750 nm. The synthesized compounds (20 μL) were mixed with 80 μL of diluted ABTS•+ solution. After 2 h of incubation, the absorbance was read at 750 nm. The percentage inhibition was calculated by [(A_0_ − A_1_)/A_0_] × 100, where A_0_ is the absorbance without the sample, and A_1_ is the absorbance with the sample. The SC_50_ value was determined from a plot of percentage inhibition versus sample concentration. Butylated hydroxytoluene (BHT) was used as the standard control and the experiment was performed in triplicate.

### 3.7. X-ray Crystallographic Analysis

Single crystal X-ray diffraction data were collected at 296(2) K on a Bruker X8 Prospector Kappa CCD diffractometer using an IìS X-ray microfocus source with multilayer mirrors, yielding intense monochromatic Cu-Ká radiation (ë = 1.54178 Å) for **2α-7f** and on a Bruker X8 APEX II Kappa CCD diffractometer using graphite monochromatized Mo-Ká radiation (ë = 0.71073 Å) for **2β-7fBr**. The structures were solved using SHELXTL XT 2013/1 [16], expanded using the difference Fourier method, and refined using full-matrix least squares on F2 with SHELXTL XLMP 2014/7 [17]. Absolute configurations of the two compounds were ambiguously determined with the estimated Flack parameters (x’s) [18] that are statistically close to zero; the corresponding respective values are 0.040(19) and 0.026(12). A summary of selected crystallographic data for **2α-7f** and **2β-7fBr** is given in Table S2.

### 3.8. Computational Study

Complete geometry optimization was carried out with the density functional theory (DFT) calculations using the popular hybrid method (B3LYP) with the 6-31 + G(d,p) basis set. The methanol phase and the polarizable continuum model (PCM) calculations were performed using the Gaussian09 package (Gaussian, Inc.: Wallingford, CT, USA) [19] with default convergence criteria.

## 4. Conclusions

In conclusion, a new series of thioether and ether furofuran lignans were obtained by nucleophilic substitution between starting samin (**5**) and nucleophiles (thiols and alcohols) under acidic conditions. This synthetic approach provided 28 new furofuran lignans. Additionally, ether furofuran lignans displayed a diastereoselectivity higher than that of the thioether furofuran lignans. All synthesized compounds were fully characterized via different spectroscopic techniques (HRESI-MS and NMR) and single crystal X-ray diffraction. The reaction mechanisms of samin and alcohol nucleophiles were investigated for the first time. The NMR monitoring and DFT calculations suggested that the products were presumably formed through the S_N_1-like mechanism by protonation of the hemiacetal center of samin (**5**) to generate the corresponding oxocarbenium ion as the intermediate. The subsequent reaction of this oxocarbenium ion with the alcohols then led to the observed only α-form either directly, or alternatively, by protonation of their epimer (β-form) through the S_N_2-like transition state contributed by acid-catalysis. Furofuran lignans **2α-7f** and **2α-7o**, with a phenolic hydroxyl group, displayed remarkable inhibitory activities against rat intestinal maltase and sucrase as well as free radicals (DPPH and ABTS). Furthermore, the active **2α-7f** and **2α-7o** were selected as representatives for the kinetic analysis. The kinetic results revealed that **2α-7f** and **2α-7o** inhibited maltase and sucrase by mixed-type and noncompetitive inhibition. The present investigation provides fundamental clues to the structural motifs required for synthesizing a new series of improved inhibitors and a practical synthetic approach. 

## Figures and Tables

**Table 1:** Nucleophilic substitution of samin with thiols *^a^*.

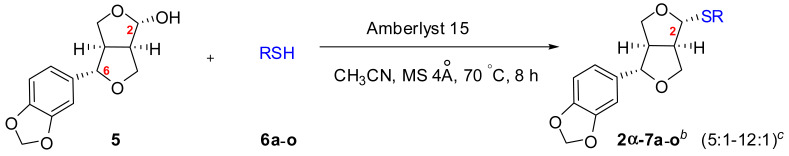
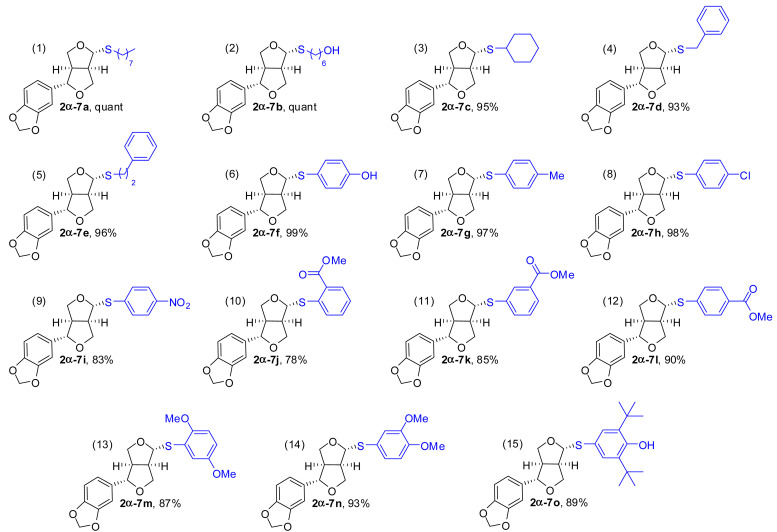

*^a^* Reaction conditions: Unless otherwise mentioned, **5** (1.0 mmol), thiols **6a**-**o** (2.0 mmol), and Amberlyst-15 (200 mg/mmol of **5**) were stirred at 70 °C for 8 h. The crude mixture was further purified using column chromatography. *^b^* Isolated yield of ***α*-7**. *^c^* dr (*α*:*β*) determined by ^1^H NMR analysis of crude reaction mixture.

**Table 2:** Nucleophilic substitution of samin with Oxygen nucleophiles *^a^*.

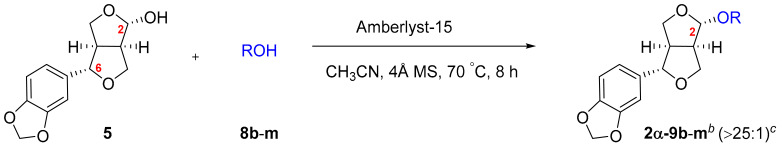
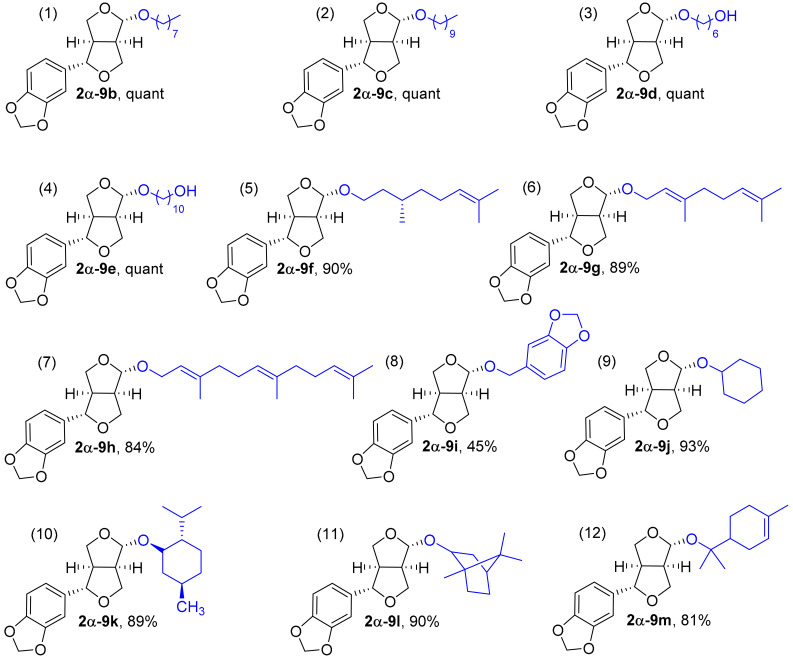

*^a^* Reaction conditions: Unless otherwise mentioned, **5** (1.0 mmol), alcohols **8b**-**m** (2.0 mmol), and Amberlyst-15 (200 mg/mmol of **5**) were stirred at 70 °C for 8 h. The crude mixture was further purified using column chromatography. *^b^* Isolated yield. *^c^* dr (α:β) determined by ^1^H NMR analysis of crude reaction mixture.

**Table 3:** α-Glucosidase inhibitory effect and radical scavenging activity of furofuran lignans.

Compound	α-Glucosidase Inhibitory Effect (IC_50_, mM) ^a^		Radical Scavenging (SC_50_, mM) ^a^	
	Maltase	Sucrase	DPPH	ABTS
**2α-7a**	>30	>30	>10	>10
**2α-7b**	>30	>30	>10	>10
**2α-7c**	>30	>30	>10	>10
**2α-7d**	>30	>30	>10	>10
**2α-7e**	>30	>30	>10	>10
**2α-7f**	14.7 ± 1.0	16.2 ± 0.9	0.93 ± 0.7	0.36 ± 0.5
**2β-7f**	14.2 ± 0.8	15.9 ± 0.7	0.91 ± 0.3	0.29 ± 0.6
**2α-7g**	>30	>30	>10	>10
**2α-7h**	>30	>30	>10	>10
**2α-7i**	>30	>30	>10	>10
**2α-7j**	>30	>30	>10	>10
**2α-7k**	>30	>30	>10	>10
**2α-7l**	>30	>30	>10	>10
**2α-7m**	>30	>30	>10	>10
**2α-7n**	>30	>30	>10	>10
**2α-7o**	11.8 ± 0.3	15.7 ± 0.7	0.87 ± 0.6	0.20 ± 0.5
**2α-9b**	>30	>30	>10	>10
**2α-9c**	>30	>30	>10	>10
**2α-9d**	>30	>30	>10	>10
**2α-9e**	>30	>30	>10	>10
**2α-9f**	>30	>30	>10	>10
**2α-9g**	>30	>30	>10	>10
**2α-9h**	>30	>30	>10	>10
**2α-9i**	>30	>30	>10	>10
**2α-9j**	>30	>30	>10	>10
**2α-9k**	>30	>30	>10	>10
**2α-9l**	>30	>30	>10	>10
**2α-9m**	>30	>30	>10	>10
acarbose^®^	1.40 ± 0.2	3.20 ± 0.4	ND ^b^	ND
BHT	ND	ND	1.56 ± 0.5	0.14 ± 0.8

^a^ IC _50_ or SC _50_ values represent as mean ± SD of three determinations. ^b^ Not determined.

**Table 4:** Kinetic parameters of **2α-7f** and **2α-7o** on α-glucosidases.

Compound	Maltase			Sucrase		
	*K*_i_ (mM)	*K*_i_′ (mM)	Inhibition Type	*K*_i_ (mM)	*K*_i_′ (mM)	Inhibition Type
**2α-7f**	0.06 ± 1.3	0.14 ± 1.1	Mixed	0.16 ± 1.7	0.20 ± 1.6	Mixed
**2α-7o**	0.03 ± 0.9	0.05 ± 1.0	Mixed	0.37 ± 1.4	-	Non- competitive

## Data Availability

The data presented in this work are available in the article and Supplementary Materials.

## Supplementary Materials

The following supporting information can be downloaded at: https://www.mdpi.com/article/10.3390/molecules27249001/s1.
