# Supplementary material for: Thioether and Ether Furofuran Lignans: Semisynthesis, Reaction Mechanism, and Inhibitory Effect against α-Glucosidase and Free Radicals
Source: Molecules. 2022 Dec 17;27(24):9001. doi: 10.3390/molecules27249001 (PMC9785863; doi:10.3390/molecules27249001)
Supplement: Supplementary file 1 [file molecules-27-09001-s001.zip › molecules-1999493-supplementary.pdf]

Supplementary materials

Thioether and ether furofuran lignans: Semisynthesis, reaction mechanism and inhibitory effect against α–glucosidase and free radicals

Wisuttaya Worawalai, Nantaporn Surachaitanawat, Phonpimon Khongchai, Viwat Vchirawongkwin, Thammarat Aree, Preecha Phuwapraisirisan *

Department of Chemistry, Faculty of Science, Chulalongkorn University, Bangkok 10330, Thailand

* *Correspondence: preecha.p@chula.ac.th*

Contents

Optimization of reaction condition……………………………………………………………………………….3

Structures and NMR data for the synthesized compounds…………………………………………………………….4

NMR spectrum for the synthesized compounds…………………………………………………………………...14

Crystal data and refinement parameters of compounds **2α-7f** and **2β-7fBr**………………………………………….47

Table S1. Optimization of Reaction Conditions[a].

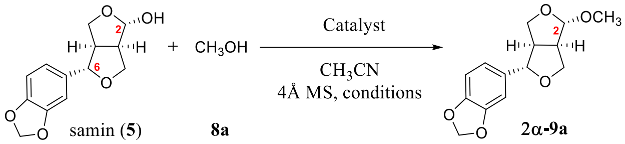

entry	catalyst (10 mol %)	conditions	yield (%)[b]
1	$AlCl_3$	rt, 2 h	96
2	$AlCl_3$	70 °C, 1.5 h	96
3	$BF_3 \cdot Et_2O$	rt, 2 h	95
4	$BF_3 \cdot Et_2O$	70 °C, 1.5 h	96
5	CH_3CO_2H	rt, 24 h	no reaction
6	CH_3CO_2H	70 °C, 24 h	no reaction
7	HCl	rt, 2 h	95
8	HCl	70 °C, 1.5 h	95
9	CF_3CO_2H	rt, 2 h	96
10	CF_3CO_2H	70 °C, 1.5 h	96
11	Amberlyst-15[c]	rt, 2 h	98
12	Amberlyst-15[c]	70 °C, 1.5 h	98

[a]Reaction conditions: **5** (1.0 mmol), CH_3OH (2.0 mmol), and catalyst were mixed in CH_3CN (0.1 M for **5**). The reaction mixture was stirred in the indicated conditions. [b]Isolated yield. [c]200 mg/mmol of **5**.

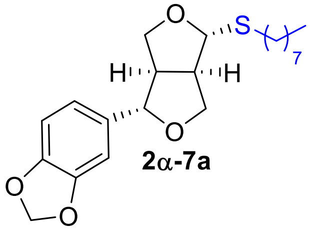

2α-7a

1-Octanethiol samin (**2α-7a**). Yield: quant., yellow oil; ^{1}H NMR (400 MHz, $CDCl_3$) δ 6.86 – 6.75 (m, 3H, H-2′, H-5′ and H-6′), 5.95 (s, 2H, H-7′), 5.38 (s, 1H, H-2), 4.38 (m, 2H, H-6 and H-8), 4.15 (dd, J = 9.3, 5.9 Hz, 1H, H-4), 3.83 (d, J = 9.4 Hz, 1H, H-4), 3.60 (m, 1H, H-8), 2.96 (m, 1H, H-1), 2.77 (m, 1H, H-5), 2.75 (m, 1H, H-1″), 2.55 (m, 1H, H-1″), 1.62 – 1.27 (m, 12H, H-CH_2), 0.88 (t, 3H, H-CH_3); ^{13}C NMR (100 MHz, $CDCl_3$) δ 148.0, 147.3, 134.6, 119.6, 108.2, 106.6, 101.1, 89.0, 86.9, 73.4, 68.3, 53.3, 53.2, 31.8, 31.1, 29.8, 29.2, 29.1, 29.0, 22.6, 14.0; HRESIMS m/z 401.1759 $[M+Na]^+$ (calcd for $C_{21}H_{30}NaO_4S$, 401.1763).

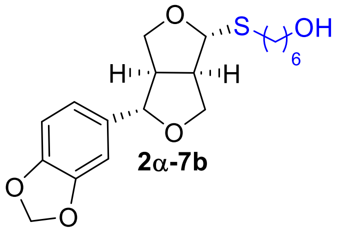

2α-7b

6-Mercapto-1-hexanol samin (**2α-7b**). Yield: quant., yellow oil; ^{1}H NMR (400 MHz, $CDCl_3$) δ 6.86 – 6.75 (m, 3H), 5.94 (s, 2H), 5.26 (s, 1H), 4.42 – 4.32 (m, 2H), 4.12 (dd, J = 9.2, 6.0 Hz, 1H), 3.84 (d, J = 9.4 Hz, 1H), 3.65 – 3.52 (m, 3H), 2.97 (m, 1H), 2.81 – 2.73 (m, 1H), 2.67 (m, 1H), 2.55 (m, 1H), 1.70 – 1.25 (m, 8H); ^{13}C NMR (100 MHz, $CDCl_3$) δ 148.0, 147.3, 134.5, 119.6, 108.2, 106.6, 101.1, 89.0, 86.9, 73.4, 68.3, 62.9, 53.3, 53.2, 32.6, 31.0, 29.7, 28.6, 25.3; HRESIMS *m/z* 389.1395 $[M+Na]^+$ (calcd for $C_{19}H_{26}NaO_5S$, 389.1399).

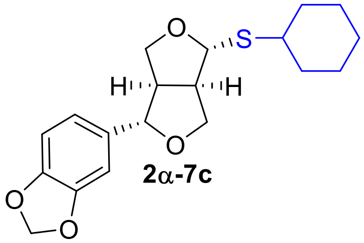

2α-7c

Cyclohexanethiol samin (**2α-7c**). Yield: 95%, yellow oil; ^{1}H NMR (400 MHz, $CDCl_3$) δ 6.86 – 6.75 (m, 3H), 5.95 (s, 2H), 5.38 (s, 1H), 4.42 – 4.35 (m, 2H), 4.15 (dd, J = 9.3, 5.9 Hz, 1H), 3.83 (d, J = 9.4 Hz, 1H), 3.60 (m, 1H), 2.96 (m, 1H), 2.76 (m, 1H), 2.12 – 1.17 (m, 11H); ^{13}C NMR (100 MHz, $CDCl_3$) δ 148.0, 147.3, 134.6, 119.6, 108.8, 106.6, 101.1, 87.9, 86.9, 73.5, 68.2, 53.4, 53.3, 43.4, 34.0, 33.8, 26.1, 26.0, 25.8; HRESIMS *m/z* 371.1297 $[M+Na]^+$ (calcd for $C_{19}H_{24}NaO_4S$, 371.1293).

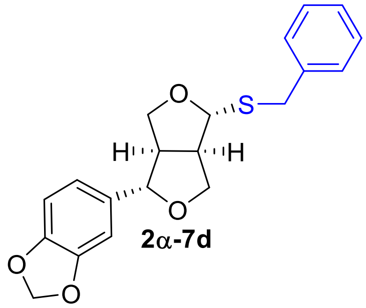

2α-7d

*Benzyl mercaptan samin (**2α-7d**)*. Yield: 93%, yellow oil; ^{1}H NMR (400 MHz, $CDCl_3$) δ 7.37 – 7.24 (m, 5H), 6.86 – 6.76 (m, 3H), 5.95 (s, 2H), 5.12 (s, 1H), 4.37 – 4.31 (m, 2H), 4.18 (dd, *J* = 9.4, 5.9 Hz, 1H), 3.91 – 3.85 (m, 2H), 3.71 (d, *J* = 13.5 Hz, 1H), 3.51 (dd, *J* = 8.9, 7.4 Hz, 1H), 2.96 (m, 1H), 2.79 (m, 1H); ^{13}C NMR (100 MHz, $CDCl_3$) δ 148.0, 147.4, 138.2, 134.5, 128.9, 128.6, 127.0, 119.6, 108.2, 106.6, 101.1, 87.5, 86.9, 73.4, 68.4, 53.3, 52.8, 34.8; HRESIMS m/z 379.0974 $[M+Na]^+$ (calcd for $C_{20}H_{20}NaO_4S$, 379.0980).

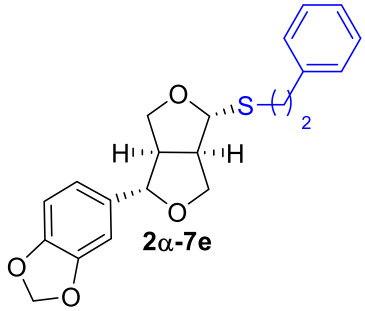

2α-7e

*Phenylethyl mercaptan samin (**2α-7e**)*. Yield: 96%, yellow oil; ^{1}H NMR (400 MHz, $CDCl_3$) δ 7.34 – 7.20 (m, 5H), 6.87 – 6.76 (m, 3H), 5.95 (s, 2H), 5.23 (s, 1H), 4.40 – 4.35 (m, 2H), 4.12 (dd, *J* = 9.4, 5.9 Hz, 1H), 3.85 (d, *J* = 9.4 Hz, 1H), 3.56 (m, 1H), 3.01 – 2.73 (m, 5H); ^{13}C NMR (100 MHz, $CDCl_3$) δ 148.2, 147.5, 140.6, 134.7, 128.7, 128.6, 126.5, 119.8, 108.3, 106.7, 101.2, 89.3, 87.0, 73.6, 68.4, 53.5, 53.3, 36.6, 32.6; HRESIMS m/z 393.1149 $[M+Na]^+$ (calcd for $C_{21}H_{22}NaO_4S$, 393.1136).

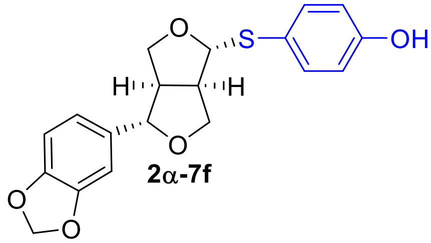

2α-7f

*4-Mercaptophenol samin (**2α-7f**)*. Yield: 99%, white powder; ^{1}H NMR (400 MHz, $CDCl_3$) δ 7.38 (d, *J* = 8.5 Hz, 2H), 6.87 – 6.71 (m, 5H), 5.95 (s, 2H), 5.37 (s, 1H), 4.44 (t, *J* = 8.7 Hz, 1H), 4.37 (d, *J* = 7.5 Hz, 1H), 4.28 (dd, *J* = 9.4, 5.9 Hz, 1H), 3.93 (d, *J* = 9.4 Hz, 1H), 3.61 (dd, *J* = 8.9, 7.1 Hz, 1H), 3.11 (m, 1H), 2.83 (m, 1H); ^{13}C NMR (100 MHz, $CDCl_3$) δ 156.1, 148.2, 147.6, 135.2, 134.4, 133.1, 119.9, 116.2, 108.4, 106.7, 101.2, 93.2, 87.0, 73.5, 68.7, 53.3, 53.0; HRESIMS *m/z* 381.0771 $[M+Na]^+$ (calcd for $C_{19}H_{18}NaO_5S$, 381.0773).

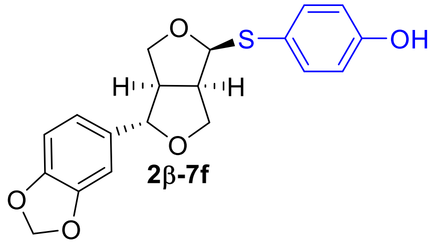

2β-7f

*4-Mercaptophenol samin (**2β-7f**)*. Yield: 34%, white powder; ^{1}H NMR (400 MHz, $CDCl_3$) δ 7.43 (d, *J* = 8.6 Hz, 2H), 6.86 – 6.75 (m, 5H), 5.95 (s, 2H), 5.09 (d, *J* = 6.1 Hz, 1H), 4.54 (d, *J* = 6.7 Hz, 1H), 4.21 (dd, *J* = 9.4, 8.1 Hz, 1H), 4.06-3.99 (m, 2H), 3.81 (dd, *J* = 9.5, 6.7 Hz, 1H), 3.36 (m, 1H), 2.84 (m, 1H); ^{13}C NMR (100 MHz, $CDCl_3$) δ 155.9, 148.1, 147.4, 134.8, 134.6, 125.6, 119.7, 116.3, 108.3, 106.7, 101.2, 90.9, 86.8, 70.6, 69.2, 54.0, 50.7; HRESIMS *m/z* 381.0770 $[M+Na]^+$ (calcd for $C_{19}H_{18}NaO_5S$, 381.0773).

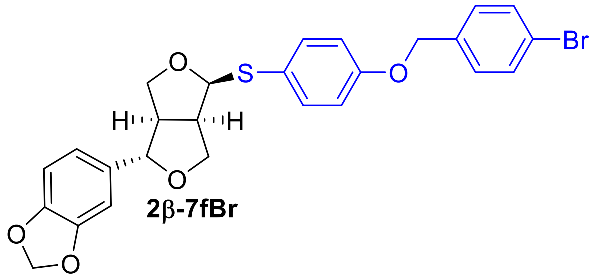

2β-7fBr

*Preparation of **2β-7fBr***: A solution of **2β-7f** (1 mmol) in acetonitrile (15 mL) was treated with 4-bromobenzylbromide (1 mmol) and K_2CO_3 (3 mmol). After stirring at 70ºC for 4 h, the reaction mixture was evaporated to dryness and purified by silica gel column (10%EtOAc-hexane) to give the product as a white solid. This product was further recrystallized using MeOH to afford **2β-7fBr** (80%) as a white crystal; ^{1}H NMR (400 MHz, $CDCl_3$) δ 7.50 (dd, *J* = 11.3, 8.6 Hz, 4H), 7.29 (d, *J* = 8.2 Hz, 2H), 6.91 (d, *J* = 8.7 Hz, 2H), 6.85 (s, 1H), 6.82-6.76 (m, 2H), 5.95 (s, 2H), 5.11 (d, *J* = 6.1 Hz, 1H), 5.00 (s, 2H), 4.54 (d, *J* = 6.7 Hz, 1H), 4.21 (t, *J* = 8.7 Hz, 1H), 4.02 (dd, *J* = 17.2, 9.7 Hz, 2H), 3.81 (dd, *J* = 9.4, 6.7 Hz, 1H), 3.38 (dd, *J* = 15.7, 8.0 Hz, 1H), 2.88-2.82 (m, 1H); HRESIMS *m/z* 549.0349, 551.0329 $[M+Na]^+$ (calcd for $C_{26}H_{23}{}^{79}BrNaO_5S$, 549.0347 and $C_{26}H_{23}{}^{81}BrNaO_5S$, 551.0327).

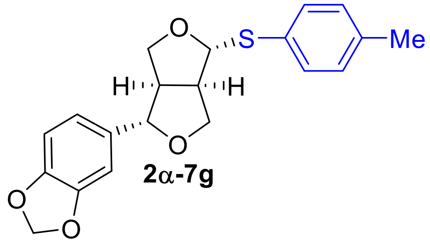

2α-7g

*4-Methylthiophenol samin (**2α-7g**)*. Yield: 97%, white powder; ^{1}H NMR (400 MHz, $CDCl_3$) δ 7.40 (d, *J* = 7.7 Hz, 2H), 7.13 (d, *J* = 7.8 Hz, 2H), 6.88 – 6.76 (m, 3H), 5.96 (s, 2H), 5.47 (s, 1H), 4.44 (t, *J* = 8.7 Hz, 1H), 4.36 (d, *J* = 7.5 Hz, 1H), 4.26 (dd, *J* = 9.4, 5.9 Hz, 1H), 3.92 (d, *J* = 9.5 Hz, 1H), 3.61 (m, 1H), 3.12 (m, 1H), 2.83 (m, 1H), 2.33 (s, 3H); ^{13}C NMR (100 MHz, $CDCl_3$) δ 148.2, 147.5, 137.7, 134.6, 132.3, 131.2, 129.8, 119.8, 108.3, 106.7, 101.2, 92.5, 87.0, 73.5, 68.7, 53.4, 53.3, 21.2; HRESIMS m/z 379.0987 $[M+Na]^+$ (calcd for $C_{20}H_{20}NaO_4S$, 379.0980).

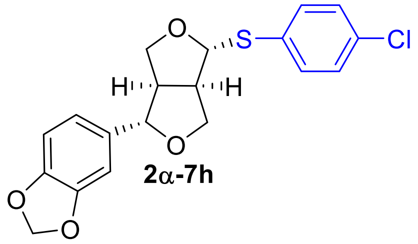

2α-7h

4-Chlorothiophenol samin (**2*α-7h***). Yield: 98%, yellow oil; ^{1}H NMR (400 MHz, $CDCl_3$) δ 7.40 (d, *J* = 8.4 Hz, 2H), 7.25 (d, J = 8.4 Hz, 2H), 6.84 – 6.73 (m, 3H), 5.93 (s, 2H), 5.46 (s, 1H), 4.42 (t, *J* = 8.7 Hz, 1H), 4.34 (d, *J* = 7.5 Hz, 1H), 4.21 (dd, *J* = 9.5, 5.9 Hz, 1H), 3.91 (d, *J* = 9.6 Hz, 1H), 3.59 (dd, *J* = 9.0, 7.1 Hz, 1H), 3.09 (m, 1H), 2.81 (m, 1H); ^{13}C NMR (100 MHz, $CDCl_3$) δ 148.0, 147.4, 134.3, 133.5, 133.4, 132.8, 129.0, 119.6, 108.2, 106.5, 101.1, 92.0, 86.9, 73.3, 68.7, 53.2, 53.1.

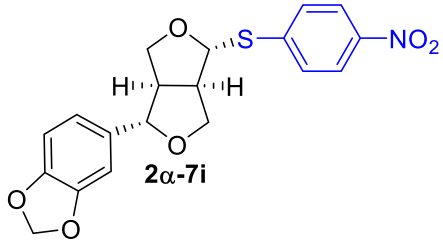

2α-7i

*4-nitrothiophenol samin (***2*α-7i***)*. Yield: 83%, yellow powder; ^{1}H NMR (400 MHz, $CDCl_3$) δ 8.13 (d, *J* = 8.8 Hz, 2H), 7.59 (d, *J* = 8.8 Hz, 2H), 6.87 – 6.75 (m, 3H), 5.95 (s, 2H), 5.73 (s, 1H), 4.48 (t, *J* = 8.8 Hz, 1H), 4.41 (d, *J* = 7.5 Hz, 1H), 4.21 (dd, *J* = 9.7, 5.8 Hz, 1H), 3.99 (d, *J* = 9.6 Hz, 1H), 3.67 (dd, *J* = 9.1, 7.0 Hz, 1H), 3.16 (m, 1H), 2.88 (m, 1H).; ^{13}C NMR (100 MHz, $CDCl_3$) δ 148.2, 147.6, 146.2, 145.8, 134.2, 128.8, 124.0, 119.8, 108.4, 106.6, 101.3, 90.6, 87.0, 73.4, 69.2, 53.3, 53.2.

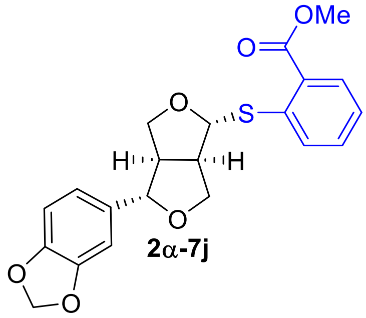

2α-7j

*Methyl thiosalicylate samin (***2*α-7j***)*. Yield: 78%, white powder; ^{1}H NMR (400 MHz, $CDCl_3$) δ 7.93 (dd, *J* = 7.8, 1.1 Hz, 1H), 7.85 (d, *J* = 8.1 Hz, 1H), 7.47 (m, 1H), 7.23 (dd, *J* = 14.2, 6.9 Hz, 1H), 6.88 – 6.75 (m, 3H), 5.95 (s, 2H), 5.68 (s, 1H), 4.48 – 4.41 (m, 2H), 4.23 (dd, *J* = 9.5, 5.8 Hz, 1H), 3.99 – 3.89 (m, 4H), 3.70 (dd, *J* = 9.1, 6.8 Hz, 1H), 3.24 (m, 1H), 2.87 (m, 1H); ^{13}C NMR (100 MHz, $CDCl_3$) δ 167.0, 148.1, 147.4, 139.9, 134.3, 132.4, 130.9, 128.8, 128.4, 125.1, 119.6, 108.2, 106.5, 101.1, 89.8, 86.8, 73.5, 68.9, 53.3, 53.0, 52.2; HRESIMS m/z 423.0884 $[M+Na]^+$ (calcd for $C_{21}H_{20}NaO_6S$, 423.0878).

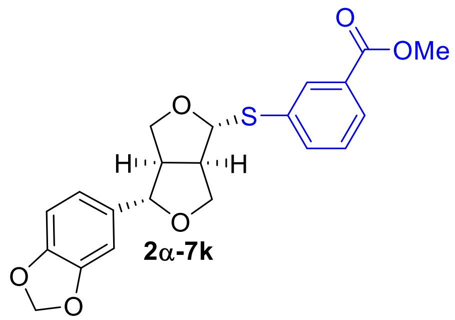

2α-7k

*Methyl 3-mercaptobenzoate samin (**2α-7k**)*. Yield: 85%, white powder; ^{1}H NMR (400 MHz, $CDCl_3$) δ 8.16 (s, 1H), 7.92 (d, *J* = 7.7 Hz, 1H), 7.68 (d, *J* = 7.8 Hz, 1H), 7.38 (t, *J* = 7.8 Hz, 1H), 6.88 – 6.75 (m, 3H), 5.95 (s, 2H), 5.58 (s, 1H), 4.46 (t, *J* = 8.7 Hz, 1H), 4.38 (d, *J* = 7.5 Hz, 1H), 4.26 (dd, *J* = 9.5, 5.9 Hz, 1H), 3.96 (d, *J* = 9.6 Hz, 1H), 3.92 (s, 3H), 3.64 (dd, *J* = 9.0, 7.1 Hz, 1H), 3.14 (m, 1H), 2.85 (m, 1H); ^{13}C NMR (100 MHz, $CDCl_3$) δ 166.5, 148.1, 147.4, 135.7, 135.5, 134.3, 132.2, 130.9, 130.8, 128.9, 128.3, 119.6, 108.2, 106.5, 101.1, 91.8, 86.8, 73.3, 68.7, 53.2, 52.2; HRESIMS m/z 423.0884 $[M+Na]^+$ (calcd for $C_{21}H_{20}NaO_6S$, 423.0878).

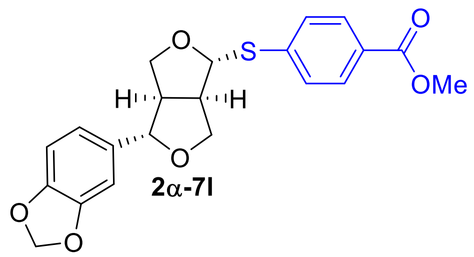

2α-7l

*Methyl 4-mercaptobenzoate samin (**2α-7l**)*. Yield: 90%, white powder; ^{1}H NMR (400 MHz, $CDCl_3$) δ 7.95 (d, *J* = 8.4 Hz, 2H), 7.53 (d, *J* = 8.4 Hz, 2H), 6.88 – 6.76 (m, 3H), 5.96 (s, 2H), 5.68 (s, 1H), 4.47 (t, *J* = 8.7 Hz, 1H), 4.40 (d, *J* = 7.5 Hz, 1H), 4.23 (m, 1H), 3.97 (d, *J* = 9.6 Hz, 1H), 3.91 (s, 3H), 3.67 (dd, *J* = 9.0, 7.1 Hz, 1H), 3.15 (m, 1H), 2.86 (m, 1H); ^{13}C NMR (100 MHz, $CDCl_3$) δ 166.7, 148.1, 147.5, 142.1, 134.2, 129.9, 129.0, 128.8, 119.7, 108.2, 106.5, 101.1, 90.8, 86.9, 73.4, 68.9, 53.2, 53.1, 52.1; HRESIMS m/z 423.0884 $[M+Na]^+$ (calcd for $C_{21}H_{20}NaO_6S$, 423.0878).

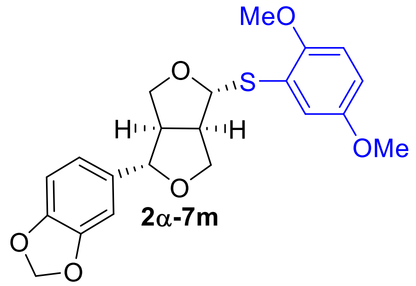

2α-7m

*2,5-Dimethoxythiophenol samin (**2α-7m**)*. Yield: 87%, colorless oil; ^{1}H NMR (400 MHz, $CDCl_3$) δ 7.17 (d, *J* = 2.6 Hz, 1H), 6.87 – 6.74 (m, 5H), 5.94 (s, 2H), 5.68 (s, 1H), 4.44 (t, *J* = 8.7 Hz, 1H), 4.37 (d, *J* = 7.6 Hz, 1H), 4.23 (dd, *J* = 9.5, 5.9 Hz, 1H), 3.91 (d, *J* = 9.5 Hz, 1H), 3.84 (s, 3H, H-OCH_3), 3.77 (s, 3H, H-OCH_3), 3.64 (dd, *J* = 8.9, 7.2 Hz, 1H), 3.17 (m, 1H), 2.84 (m, 1H); ^{13}C NMR (100 MHz, $CDCl_3$) δ 153.9, 152.1, 148.0, 147.4, 134.4, 124.0, 119.6, 118.0, 112.9, 111.7, 108.2, 106.6, 101.1, 89.8, 86.9, 73.4, 68.8, 56.5, 55.8, 53.3, 53.2; HRESIMS m/z 425.1048 $[M+Na]^+$ (calcd for $C_{21}H_{22}NaO_6S$, 425.1035).

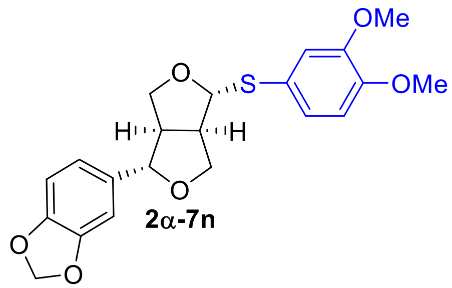

2α-7n

*3,4-Dimethoxythiophenol samin (**2α-7n**)*. Yield: 87%, colorless oil; ^{1}H NMR (400 MHz, $CDCl_3$) δ 7.09 (dd, *J* = 8.3, 1.8 Hz, 1H), 7.05 (d, *J* = 1.6 Hz, 1H) 6.81 (m, 4H), 5.95 (s, 2H), 5.42 (s, 1H), 4.44 (t, *J* = 8.7 Hz, 1H), 4.37 (d, *J* = 7.5 Hz, 1H), 4.27 (dd, *J* = 9.4, 5.9 Hz, 1H), 3.93 (d, *J* = 9.5 Hz, 1H), 3.89 (s, 3H, H-OCH_3), 3.87 (s, 3H, H-OCH_3), 3.62 (dd, *J* = 8.9, 7.1 Hz, 1H), 3.11 (m, 1H), 2.82 (m, 1H); ^{13}C NMR (100 MHz, $CDCl_3$) δ 149.3, 149.1, 148.0, 147.4, 134.4, 125.8, 125.5, 119.6, 116.1, 111.6, 108.2, 106.5, 101.9, 92.9, 86.8, 73.4, 68.6, 56.0, 56.0, 53.3, 53.0; HRESIMS m/z 425.1048 $[M+Na]^+$ (calcd for $C_{21}H_{22}NaO_6S$, 425.1035).

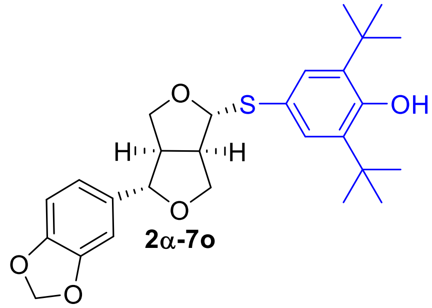

2α-7o

*2,6-Di-tert-butyl-4-mercaptophenol samin (**2α-7o**)*. Yield: 89%, yellow oil; ^{1}H NMR (400 MHz, $CDCl_3$) δ 7.33 (s, 2H), 6.87 – 6.75 (m, 3H), 5.95 (s, 2H), 5.39 (s, 1H), 4.43 (t, *J* = 8.7 Hz, 1H), 4.37 (d, *J* = 7.5 Hz, 1H), 4.29 (dd, *J* = 9.4, 5.9 Hz, 1H), 3.93 (d, *J* = 9.4 Hz, 1H), 3.61 (m, 1H), 3.11 (m, 1H), 2.81 (m, 1H), 1.43 (s, 18H); ^{13}C NMR (100 MHz, $CDCl_3$) δ 154.2, 148.2, 147.5, 136.7, 130.4, 127.9, 119.8, 108.3, 106.7, 101.2, 93.1, 86.98, 73.5, 68.5, 53.5, 53.2, 34.5, 30.3; HRESIMS m/z 493.2024 $[M+Na]^+$ (calcd for $C_{27}H_{34}NaO_5S$, 493.2025).

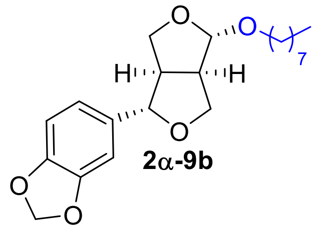

2α-9b

*Octanol samin (**2α-9b**)*. Yield: quant., pale yellow oil; ^{1}H NMR (400 MHz, $CDCl_3$) δ 6.85 (d, *J* = 0.8 Hz, 1H), 6.80 (dd, *J* = 8.0, 1.2 Hz, 1H), 6.78 (d, *J* = 7.6 Hz, 1H), 5.94 (s, 2H), 4.93 (s, 1H), 4.38 (d, *J* = 8.8 Hz, 1H), 4.34 (t, *J* = 7.2 Hz, 1H), 3.97 (dd, *J* = 8.8, 6.0 Hz, 1H), 3.85 (d, *J* = 8.8 Hz, 1H), 3.65 (m, 1H), 3.55 (dd, *J* = 8.8, 7.6 Hz, 1H), 3.37 (m, 1H), 3.04 (q, *J* = 8.8 Hz, 1H), 2.81 (q, *J* = 9.2 Hz, 1H), 1.59-1.54 (m, 4H), 1.27 (overlap, 8H), 0.87 (t, *J* = 6.8 Hz, 3H); ^{13}C NMR (100 MHz, $CDCl_3$) δ 148.1, 147.4, 134.9, 119.8, 108.3, 107.6, 106.7, 101.2, 87.2, 71.6, 69.0, 67.6, 53.2, 52.9, 31.9, 29.8, 29.5, 29.4, 26.3, 22.8, 14.2; HRESIMS *m/z* 385.1997 $[M+Na]^+$ (calcd for $C_{21}H_{30}NaO_5$, 385.1991).

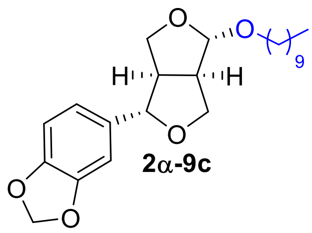

2α-9c

*Decanol samin (**2α-9c**).* Yield: quant., colorless oil; ^{1}H NMR (400 MHz, $CDCl_3$) δ 6.85 (d, *J* = 1.6 Hz, 1H), 6.81-6.75 (m, 2H), 5.94 (s, 2H), 4.93 (s, 1H), 4.38 (d, *J* = 8.8 Hz, 1H), 4.35 (t, *J* = 7.6 Hz, 1H), 3.97 (dd, *J* = 9.2, 6.0 Hz, 1H), 3.85 (d, *J* = 8.4 Hz, 1H), 3.65 (m, 1H), 3.55 (dd, *J* = 8.8, 7.6 Hz, 1H), 3.36 (m, 1H), 3.04 (q, *J* = 8.8 Hz, 1H), 2.81 (m, 1H), 1.60-1.52 (m, 4H), 1.26 (overlap, 12H), 0.87 (t, *J* = 6.8 Hz, 3H); ^{13}C NMR (100 MHz, $CDCl_3$) δ 148.1, 147.4, 134.9, 119.8, 108.3, 107.6, 106.7, 101.2, 87.2, 71.6, 69.0, 67.6, 53.3, 52.9, 32.0, 29.8, 29.7, 29.7, 29.6, 29.4, 26.3, 22.8, 14.2; HRESIMS m/z 413.2306 $[M+Na]^+$ (calcd for $C_{23}H_{34}NaO_5$, 413.2304).

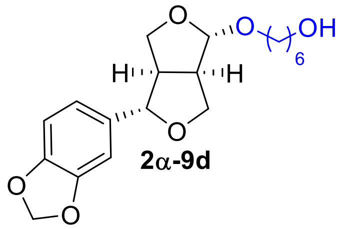

2α-9d

*1,6-Hexanediol samin (**2α-9d**).* Yield: quant., colorless oil; ^{1}H NMR (400 MHz, $CDCl_3$) δ 6.85 (s, 1H), 6.81-6.76 (m, 2H), 5.94 (s, 2H), 4.93 (s, 1H), 4.38 (d, *J* = 8.8 Hz, 1H), 4.35 (t, *J* = 8.0 Hz, 1H), 3.96 (dd, *J* = 9.2, 6.0 Hz, 1H), 3.85 (d, *J* = 8.8 Hz, 1H), 3.69-3.62 (m, 3H), 3.55 (dd, *J* = 8.8, 8.0 Hz, 1H), 3.38 (m, 1H), 3.03 (q, *J* = 8.4 Hz, 1H), 2.81 (q, *J* =8.8 Hz, 1H), 1.59-1.56 (m, 2H), 1.43-1.33 (m, 6H); ^{13}C NMR (100 MHz, $CDCl_3$) δ 148.1, 147.4, 134.9, 119.8, 108.3, 107.6, 106.7, 101.2, 87.2, 71.5, 69.1, 67.4, 64.6, 53.2, 52.9, 32.8, 29.7, 28.7, 25.9; HRESIMS *m/z* 373.1630 $[M+Na]^+$ (calcd for $C_{19}H_{26}NaO_6$, 373.1627).

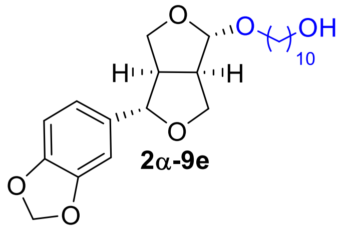

2α-9e

*1,10-Decanediol samin (**2α-9e**).* Yield: quant., colorless oil; ^{1}H NMR (400 MHz, $CDCl_3$) δ 6.85 (s, 1H), 6.81-6.75 (m, 2H), 5.94 (s, 2H), 4.93 (s, 1H), 4.38 (d, *J* = 8.8 Hz, 1H), 4.35 (t, *J* = 7.6 Hz, 1H), 3.97 (dd, *J* = 8.8, 6.0 Hz, 1H), 3.85 (d, *J* = 8.8 Hz, 1H), 3.68-3.62 (m, 3H), 3.55 (dd, *J* = 8.8, 7.6 Hz, 1H, H-8), 3.37 (m, 1H), 3.04 (q, *J* = 8.4 Hz, 1H), 2.82 (q, *J* =8.8 Hz, 1H), 1.57-1.54 (m, 2H), 1.28-1.25 (m, 14H); ^{13}C NMR (100 MHz, $CDCl_3$) δ 148.1, 147.4, 134.9, 119.8, 108.3, 107.6, 106.7, 101.2, 87.2, 71.6, 69.1, 67.6, 63.2, 53.2, 52.9, 32.9, 29.8, 29.8, 29.7, 29.6, 29.5, 26.3, 25.9; HRESIMS m/z 429.2261 $[M+Na]^+$ (calcd for $C_{23}H_{34}NaO_6$, 429.2253).

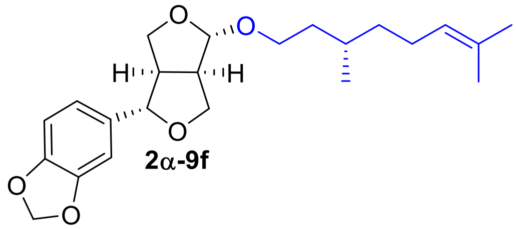

2α-9f

*β–Citronellol samin (**2α-9f**)*. Yield: 90%, colorless oil; ^{1}H NMR (400 MHz, $CDCl_3$) δ 6.85 (s, 1H), 6.81-6.76 (m, 2H), 5.94 (s, 2H), 5.09 (t, *J* = 6.8 Hz, 1H), 4.93 (s, 1H), 4.38 (d, *J* = 8.8 Hz, 1H), 4.35 (t, *J* = 7.2 Hz, 1H), 3.97 (dd, *J* = 9.8, 6.0 Hz, 1H), 3.85 (d, *J* = 8.8 Hz, 1H), 3.70 (m, 1H), 3.55 (dd, *J* = 8.8, 7.6 Hz, 1H), 3.40 (m, 1H), 3.03 (q, *J* = 9.2 Hz, 1H), 2.81 (q, *J* = 6.8 Hz, 1H), 2.03-1.91 (m, 2H), 1.68 (s, 3H), 1.59 (s, 3H), 1.54 (m, 1H), 1.41-1.30 (m, 3H), 1.16 (m, 1H), 0.89 (d, *J* = 6.4 Hz, 3H); ^{13}C NMR (100 MHz, $CDCl_3$) δ 148.1, 147.4, 135.0, 131.3, 124.9, 119.8, 108.3, 107.5, 106.7, 101.2, 87.2, 71.6, 69.1, 65.9, 53.3, 53.0, 37.4, 36.8, 29.8, 25.8, 25.6, 19.8, 17.8; HRESIMS m/z 411.2155 $[M+Na]^+$ (calcd for $C_{23}H_{32}NaO_5$, 411.2147).

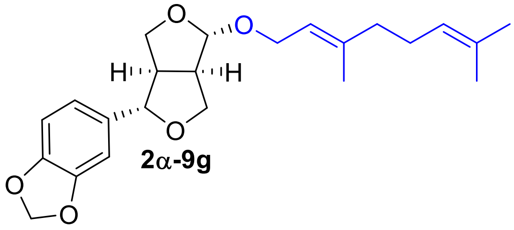

2α-9g

*Geraniol samin (**2α-9g**)*. Yield: 89%, colorless oil; ^{1}H NMR (400 MHz, $CDCl_3$) δ 6.85 (s, 1H), 6.81-6.75 (m, 2H), 5.94 (s, 2H), 5.33 (t, *J* = 6.4 Hz, 1H), 5.09 (m, 1H), 4.99 (s, 1H), 4.38 (d, *J* = 9.2 Hz, 1H), 4.35 (dd, *J* = 8.4, 7.2 Hz, 1H), 4.17 (dd, *J* = 12.0, 6.8 Hz, 1H), 4.02-3.97 (m, 2H), 3.87 (d, *J* = 8.8 Hz, 1H), 3.55 (dd, *J* = 9.2, 7.6 Hz, 1H), 3.06 (m, 1H), 2.82 (m, 1H), 2.10-2.04 (m, 4H), 1.68 (s, 6H), 1.60 (s, 3H); ^{13}C NMR (100 MHz, $CDCl_3$) δ 148.1, 147.4, 141.1, 134.9, 131.8, 124.1, 120.2, 119.8, 108.3, 106.8, 106.7, 101.2, 87.2, 71.6, 69.1, 63.6, 53.3, 53.0, 39.8, 26.5, 25.8, 17.8, 16.6; HRESIMS m/z 409.1992 $[M+Na]^+$ (calcd for $C_{23}H_{30}NaO_5$, 409.1991).

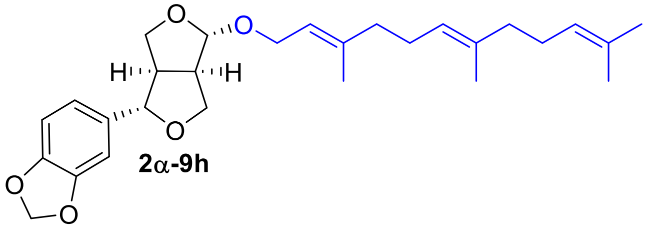

2α-9h

*trans,trans-Farnesol samin (**2α-9h**)*. Yield: 84%, colorless oil; ^{1}H NMR (400 MHz, $CDCl_3$) δ 6.85 (s, 1H), 6.79-6.75 (m, 2H), 5.94 (s, 2H), 5.33 (m, 1H), 5.10-5.07 (m, 2H), 4.98 (s, 1H), 4.38 (d, *J* = 8.8 Hz, 1H), 4.34 (dd, *J* = 9.6, 7.6 Hz, 1H), 4.18 (m, 1H), 4.01-3.98 (m, 2H), 3.86 (d, *J* = 8.8 Hz, 1H), 3.54 (dd, *J* = 8.8, 8.8 Hz, 1H), 3.06 (m, 1H), 2.81 (m, 1H), 2.12-1.95 (m, 8H), 1.69 (s, 3H), 1.67 (s, 3H), 1.59 (s, 6H); ^{13}C NMR (100 MHz, $CDCl_3$) δ 148.1, 147.4, 141.1, 135.5, 134.9, 131.4, 124.5, 123.9, 120.2, 119.8, 108.3, 106.7, 106.7, 101.2, 87.1, 71.5, 69.1, 63.6, 53.2, 53.0, 39.8, 39.7, 26.9, 26.4, 25.8, 17.8, 16.6, 16.1; HRESIMS m/z 477.2617 $[M+Na]^+$ (calcd for $C_{28}H_{38}NaO_5$, 477.2617).

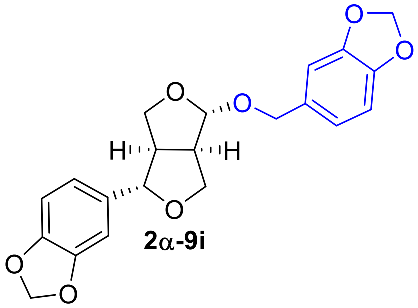

2α-9i

*Piperonyl alcohol samin (**2α-9i**)*. Yield: 45%, white solid; ^{1}H NMR (400 MHz, $CDCl_3$) δ 6.80 (m, 6H), 5.95 (d, *J* = 1.0 Hz, 4H), 5.02 (s, 1H), 4.60 (d, *J* = 11.4 Hz, 1H), 4.42 – 4.31 (m, 3H), 4.03 (dd, *J* = 9.1, 6.0 Hz, 1H), 3.90 (d, *J* = 9.0 Hz, 1H), 3.57 – 3.51 (m, 1H), 3.09 (dd, *J* = 16.9, 8.7 Hz, 1H), 2.83 (dd, *J* = 15.5, 6.7 Hz, 1H); ^{13}C NMR (100

MHz, $CDCl_3$) δ 148.1, 134.8, 131.6, 121.9, 119.8, 108.9, 108.3, 106.7, 106.5, 101.2, 101.2, 87.2, 71.5, 69.3, 68.9, 53.2, 52.9.

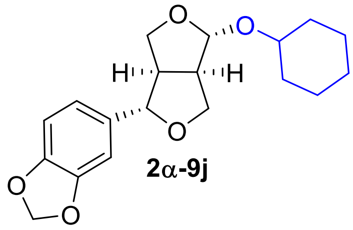

2α-9j

*Cyclohexanol samin (**2α-9j**)*. Yield: 93%, colorless oil; ^1H NMR (400 MHz, $CDCl_3$) δ 6.85 (d, *J* = 0.8 Hz, 1H), 6.81-6.75 (m, 2H), 5.94 (s, 2H), 4.93 (s, 1H), 4.37 (d, *J* = 8.8 Hz, 1H), 4.34 (dd, *J* = 8.0, 5.6 Hz, 1H), 4.01 (dd, *J* = 8.8, 6.0 Hz, 1H), 3.83 (d, *J* = 8.8 Hz, 1H), 3.55 (m, 2H), 3.02 (q, *J* = 8.4 Hz, 1H), 2.83 (m, 1H), 1.89-1.52 (m, 10H); ^{13}C NMR (100 MHz, $CDCl_3$) δ 148.1, 147.4, 135.0, 119.8, 108.3, 106.8, 105.6, 101.2, 87.3, 74.9, 71.6, 69.0, 53.5, 53.0, 34.0, 32.0, 25.8, 24.5, 24.4; HRESIMS m/z 355.1527 $[M+Na]^+$ (calcd for $C_{19}H_{24}NaO_5$, 355.1521).

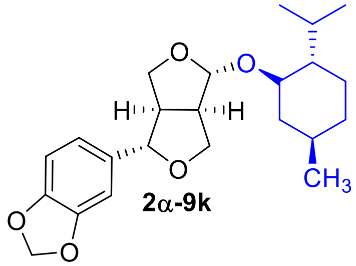

2α-9k

*α-Terpeneol samin (**2α-9k**)*. Yield: 89%, colorless oil; ^1H NMR (400 MHz, $CDCl_3$) δ 6.84 (s, 1H), 6.81-6.75 (m, 2H), 5.94 (s, 2H), 5.01 (s, 1H), 4.38 (d, *J* = 8.8 Hz, 1H), 4.35 (dd, *J* = 8.4, 6.4 Hz, 1H), 4.05 (dd, *J* = 9.2, 6.4 Hz, 1H), 3.84 (d, *J* = 8.8 Hz, 1H), 3.54 (dd, *J* = 9.2, 7.6 Hz, 1H), 3.31 (ddd, *J* = 10.4, 10.0, 4.4 Hz, 1H), 3.04 (q, *J* = 8.0 Hz, 1H), 2.83 (q, *J* = 8.8 Hz, 1H), 2.13-2.04 (m, 2H), 1.63-1.59 (m, 3H), 1.40 (m, 1H), 1.17 (m, 1H), 1.01-0.94 (m, 2H), 0.91 (d, *J* = 4.8 Hz, 3H), 0.89 (d, *J* = 4.4 Hz, 3H), 0.78 (d, *J* = 6.8 Hz, 3H); ^{13}C NMR (100 MHz, $CDCl_3$) δ 148.1, 147.4, 135.1, 119.8, 109.4, 108.3, 106.7, 101.2, 87.3, 79.7, 71.7, 69.1, 53.4, 52.9, 48.9, 43.6, 34.5, 31.9, 25.9, 23.5, 22.4, 21.3, 16.5; HRESIMS m/z 411.2143 $[M+Na]^+$ (calcd for $C_{23}H_{32}NaO_5$, 411.2147).

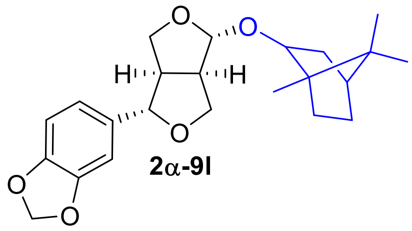

2α-9l

*Borneol samin (**2α-9l**)*. Yield: 90%, colorless oil; ^1H NMR (400 MHz, $CDCl_3$) δ 6.85 (s, 1H), 6.82-6.76 (m, 2H), 5.95 (s, 2H), 4.95 (s, 1H), 4.38 (d, *J* = 8.8 Hz, 1H), 4.34 (dd, *J* = 12.8, 8.0 Hz, 1H), 3.98 (dd, *J* = 8.8, 6.0 Hz, 1H), 3.82 (d, *J* = 8.8 Hz, 1H), 3.76 (brd, *J* = 8.0 Hz, 1H), 3.53 (dd, *J* = 8.8, 7.6 Hz, 1H), 3.07 (q, *J* = 8.4 Hz, 1H), 2.84 (q, *J* = 9.2 Hz, 1H), 2.22 (m, 1H), 1.89 (m, 1H), 1.68-1.58 (m, 2H), 1.22-1.15 (m, 2H), 1.01 (dd, *J* = 13.6, 3.2 Hz, 1H), 0.85 (s, 3H), 0.84 (s, 3H), 0.83 (s, 3H); ^{13}C NMR (100 MHz, $CDCl_3$) δ 148.1, 147.4, 135.1, 119.8, 109.2, 108.3, 106.8, 101.2, 87.2, 84.0, 71.7, 69.0, 53.3, 53.1, 49.4, 47.5, 45.2, 37.9, 28.4, 26.9, 19.9, 18.9, 14.0; HRESIMS m/z 409.1992 $[M+Na]^+$ (calcd for $C_{23}H_{30}NaO_5$, 409.1991).

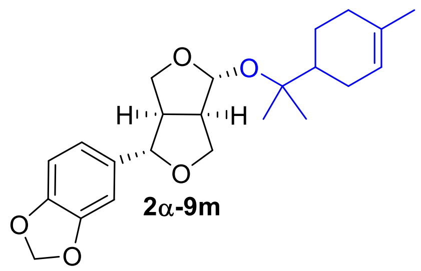

2α-9m

*α-Terpineol samin (**2α-9m**)*. Yield: 81%, colorless oil; ^{1}H NMR (400 MHz, $CDCl_3$) δ 6.84 (d, *J* = 1.6 Hz, 1H), 6.80-6.77 (m, 2H), 5.94 (s, 2H), 5.37 (brs, 1H), 5.24 (s, 1H), 4.36 (d, *J* = 5.6 Hz, 1H), 4.33 (dd, *J* = 6.0, 4.0 Hz, 1H), 4.05 (dd, *J* = 9.2, 6.0 Hz, 1H), 3.80 (d, *J* = 8.8 Hz, 1H), 3.57 (dd, *J* = 8.8, 7.2 Hz, 1H), 2.95 (m, 1H), 2.83 (m, 1H), 2.04-1.96 (m, 4H), 1.83-1.81 (m, 3H), 1.64 (s, 3H), 1.57 (s, 3H), 1.19 (s, 3H); ^{13}C NMR (100 MHz, $CDCl_3$) δ 148.1, 147.4, 135.3, 134.1, 121.0, 119.7, 108.3, 106.7, 102.3, 101.2, 87.2, 78.5, 71.8, 69.1, 54.2, 53.2, 44.2, 31.2, 26.9, 24.3, 24.0, 23.6, 23.5; HRESIMS m/z 409.1992 $[M+Na]^+$ (calcd for $C_{23}H_{30}NaO_5$, 409.1991)

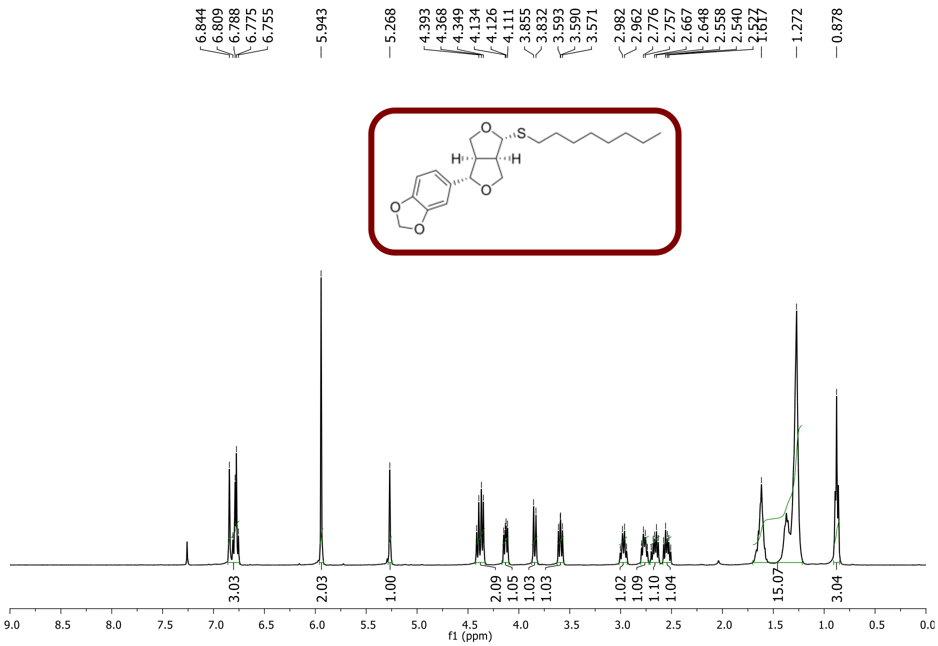

Figure S1. ^{1}H NMR spectrum of **2α-7a** ($CDCl_3$)

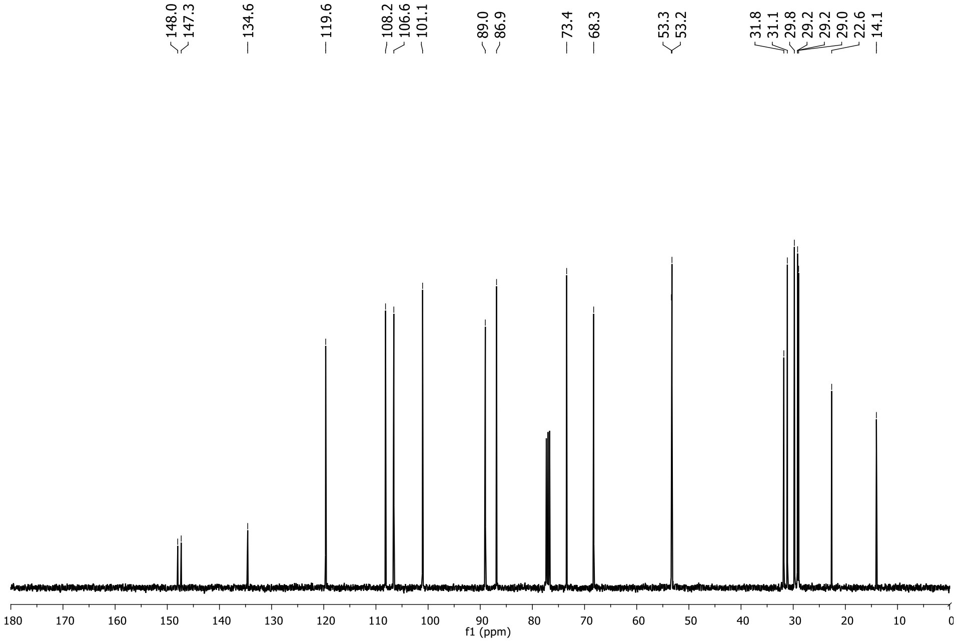

Figure S2. ^{13}C NMR spectrum of **2α-7a** ($CDCl_3$)

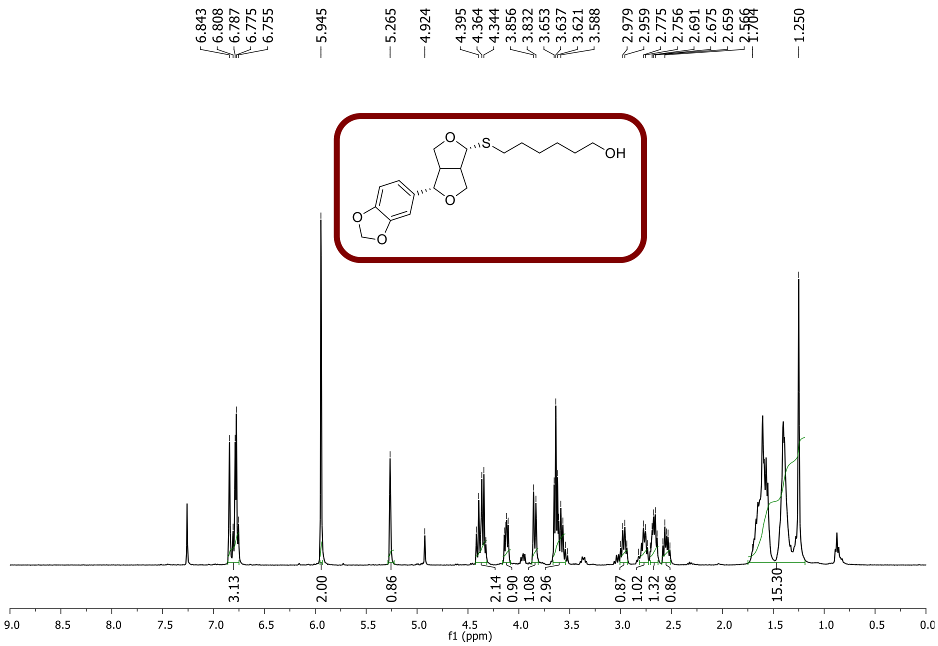

Figure S3. ^{1}H NMR spectrum of **2α-7b** ($CDCl_3$)

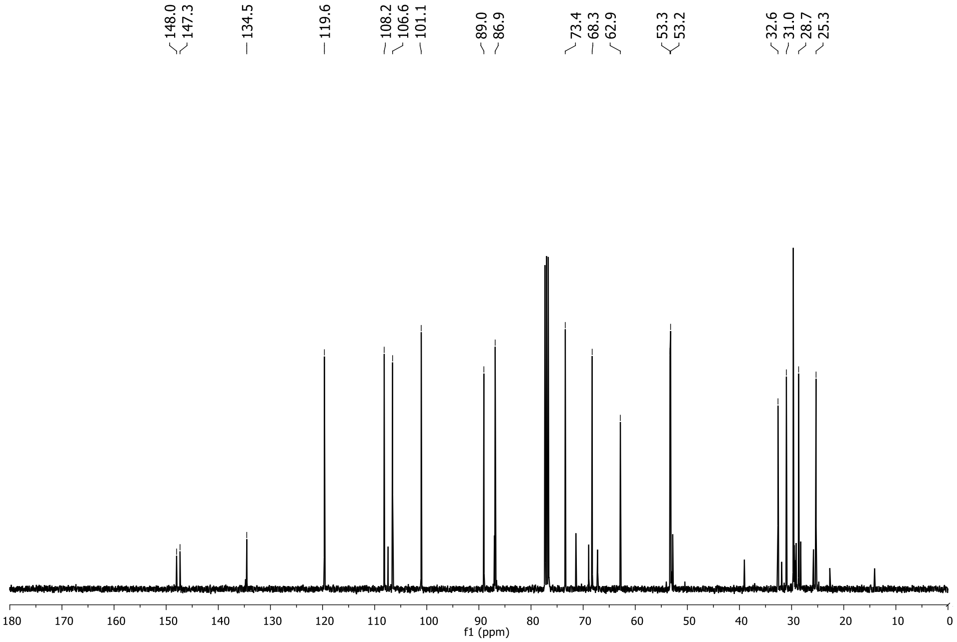

Figure S4. ^{13}C NMR spectrum of **2α-7b** ($CDCl_3$)

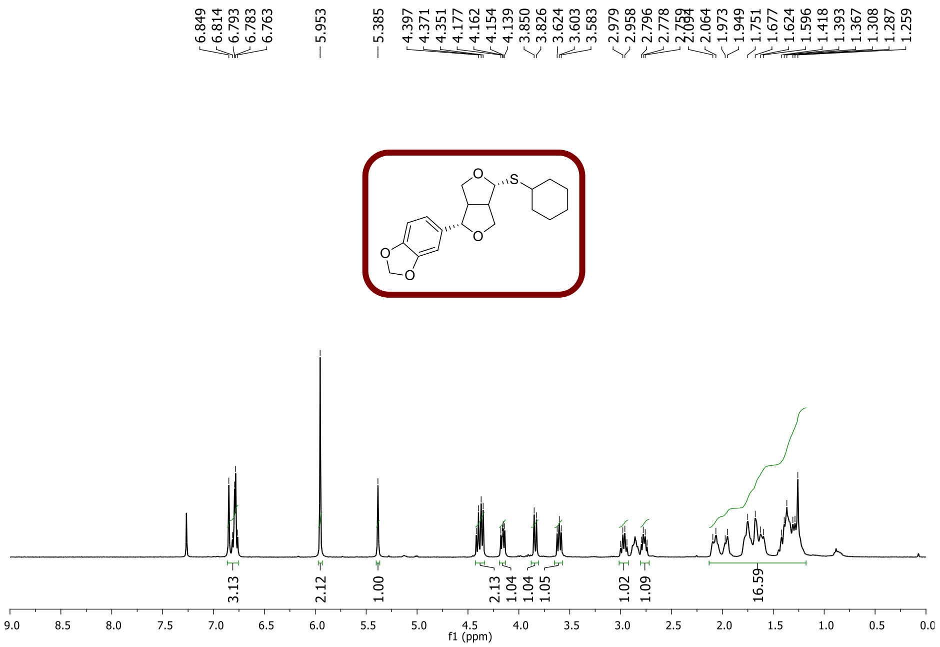

Figure S5. ^{1}H NMR spectrum of **2α-7c** ($CDCl_3$)

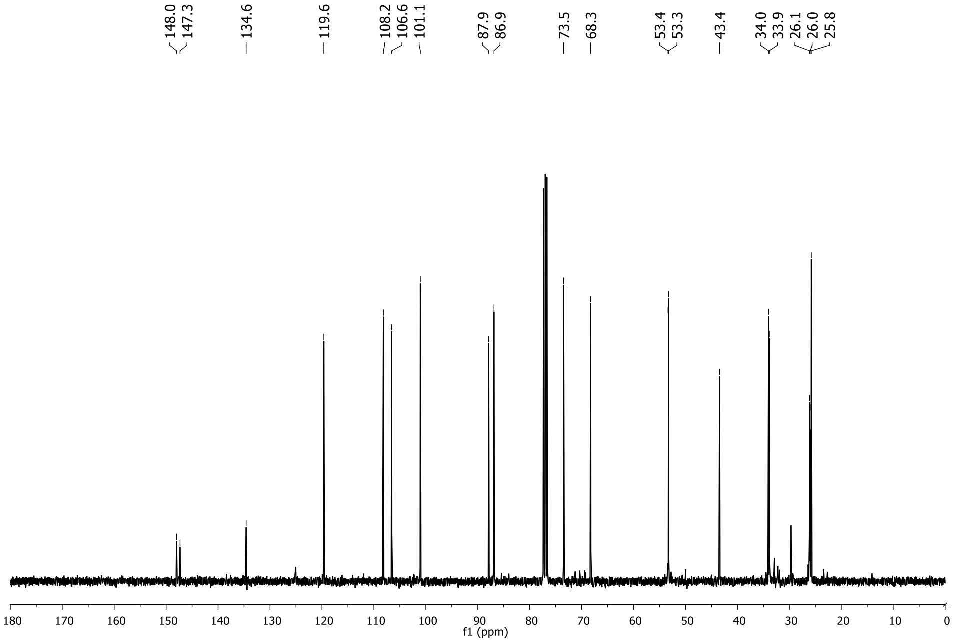

Figure S6. ^{13}C NMR spectrum of **2α-7c** ($CDCl_3$)

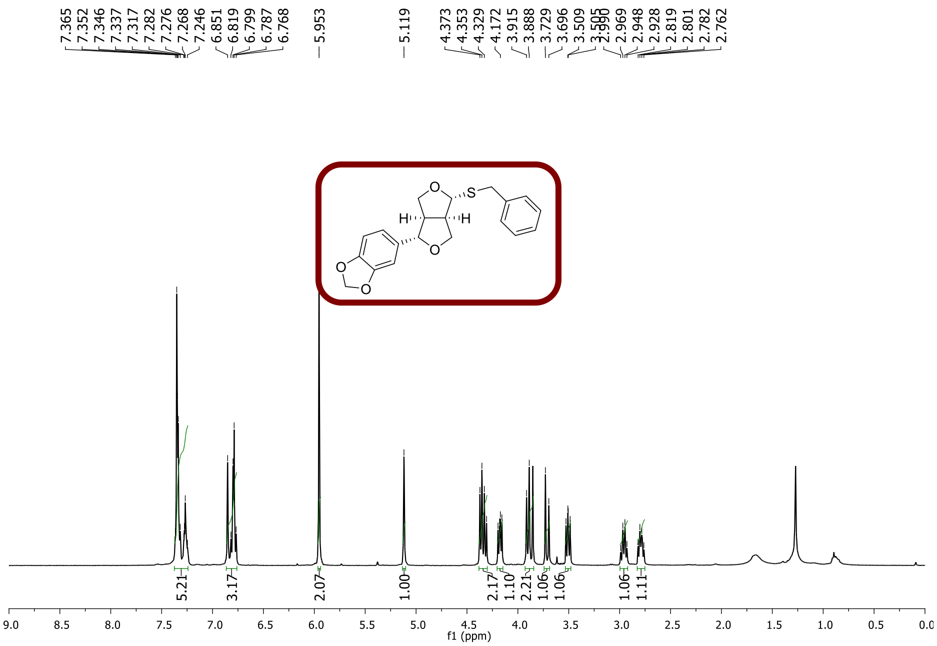

Figure S7. ^{1}H NMR spectrum of **2α-7d** ($CDCl_3$)

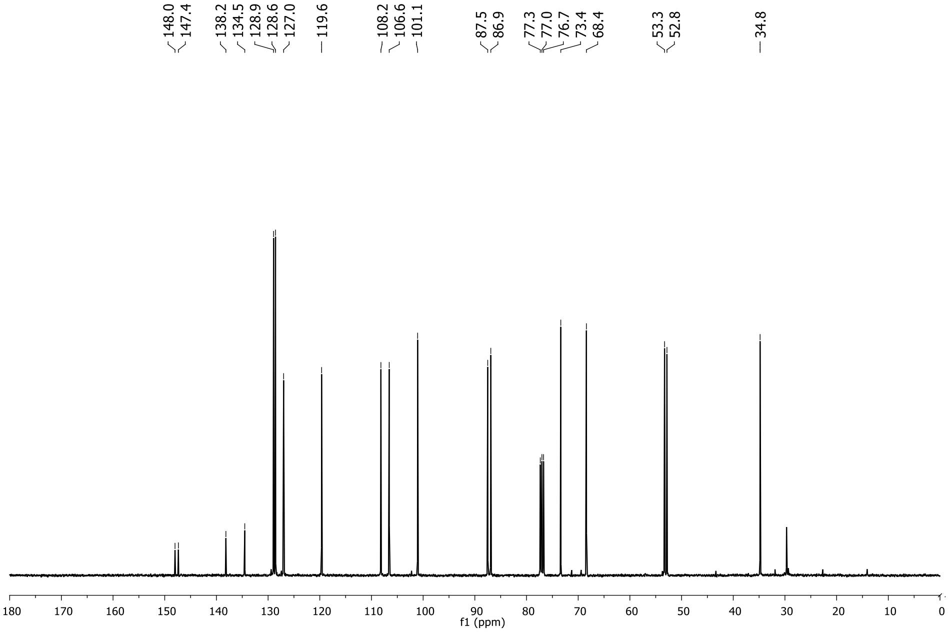

Figure S8. ^{13}C NMR spectrum of **2α-7d** ($CDCl_3$)

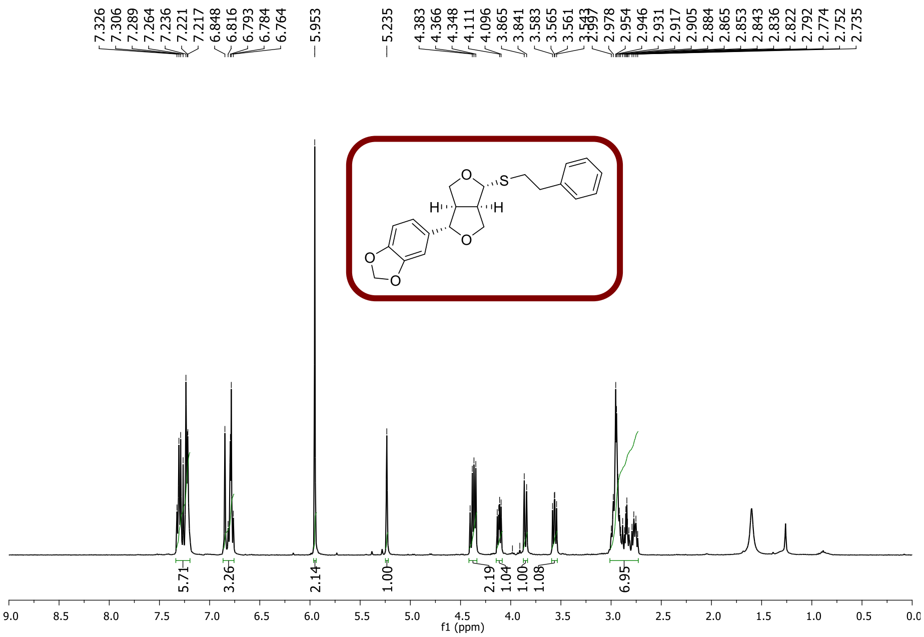

Figure S9. ^{1}H NMR spectrum of **2α-7e** ($CDCl_3$)

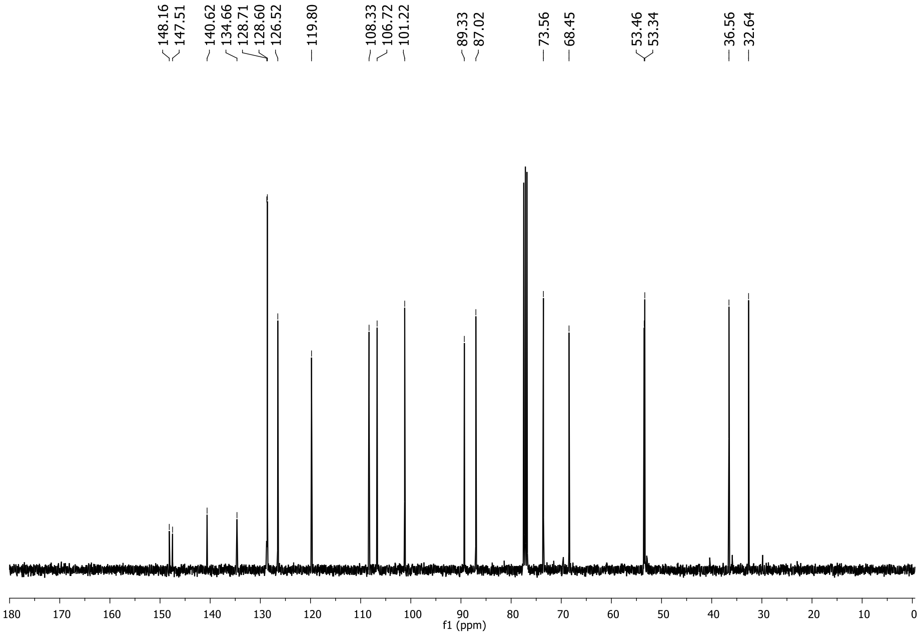

Figure S10. ^{13}C NMR spectrum of **2α-7e** ($CDCl_3$)

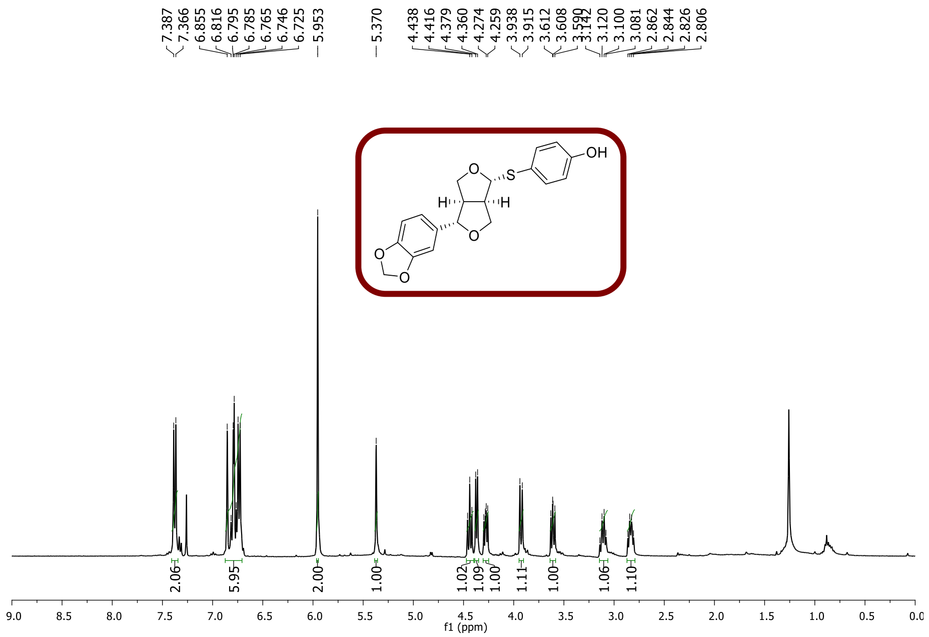

Figure S11. ^{1}H NMR spectrum of **2α-7f** ($CDCl_3$)

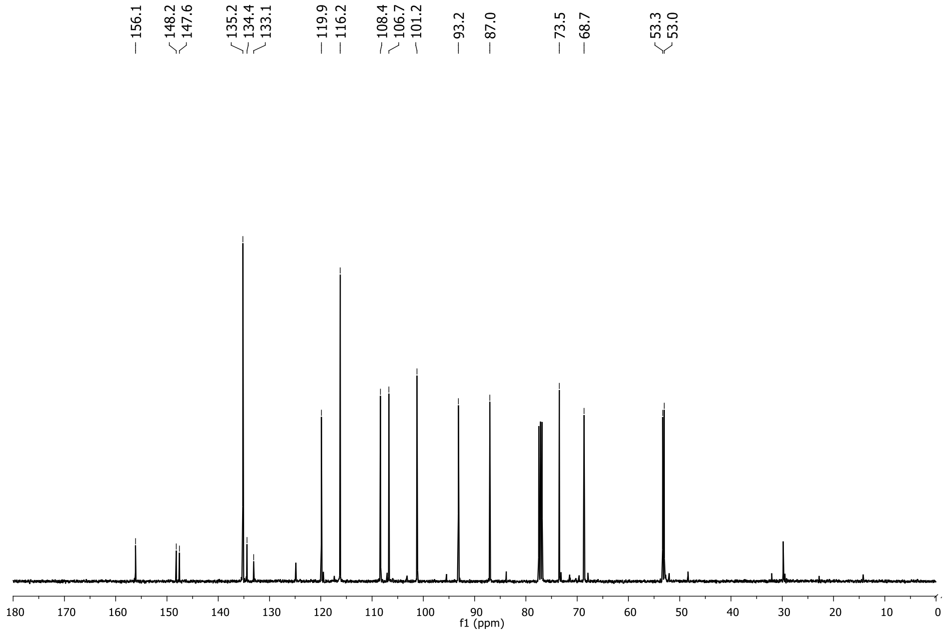

Figure S12. ^{13}C NMR spectrum of **2α-7f** ($CDCl_3$)

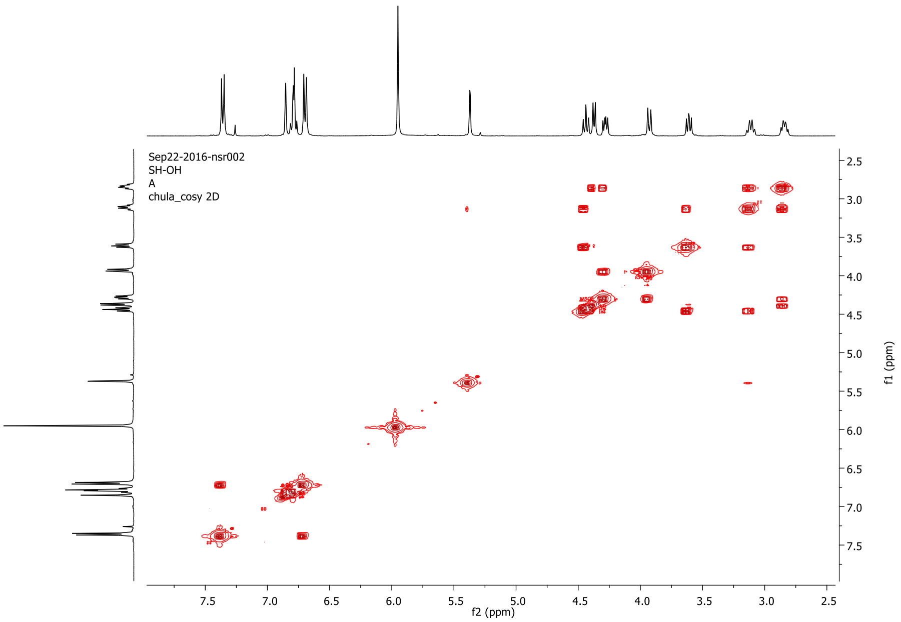

Figure S13. COSY spectrum of **2α-7f** ($CDCl_3$)

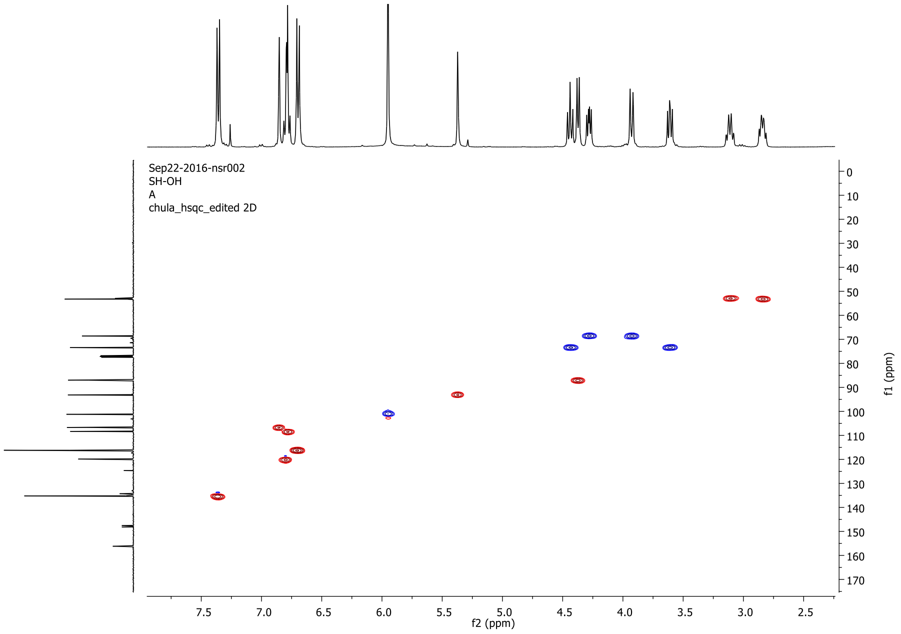

Figure S14. HSQC spectrum of **2α-7f** ($CDCl_3$)

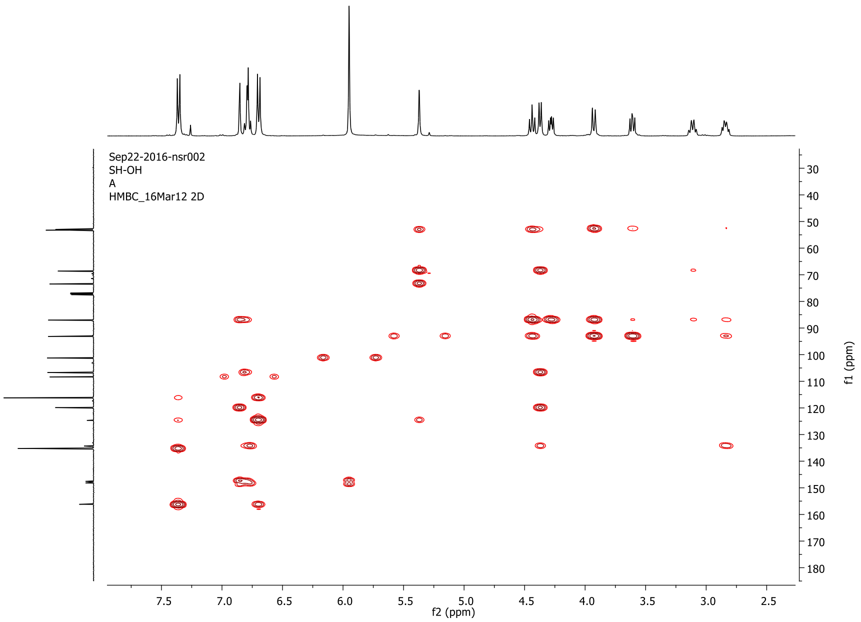

Figure S15. HMBC spectrum of **2α-7f** ($CDCl_3$)

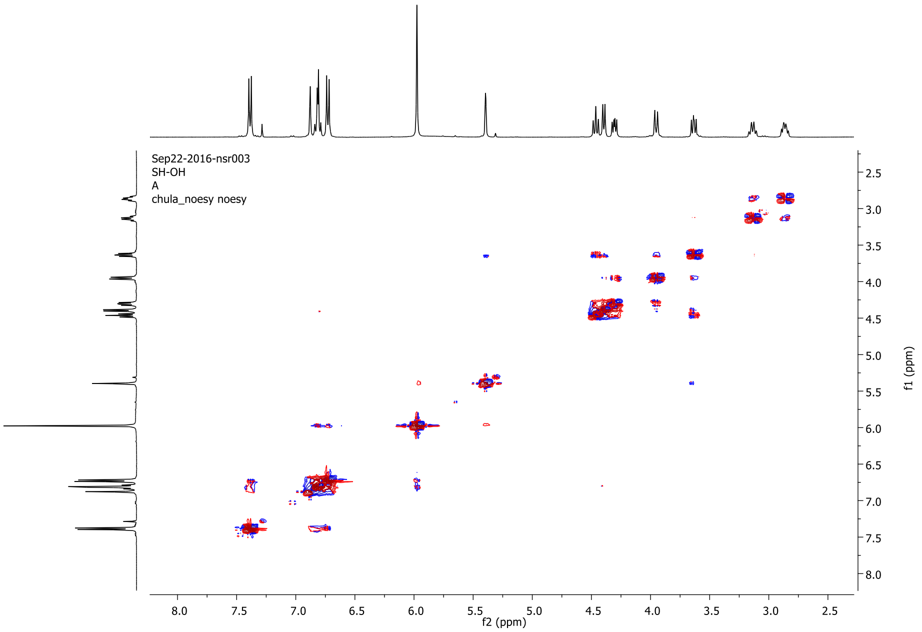

Figure S16. NOESY spectrum of **2α-7f** ($CDCl_3$)

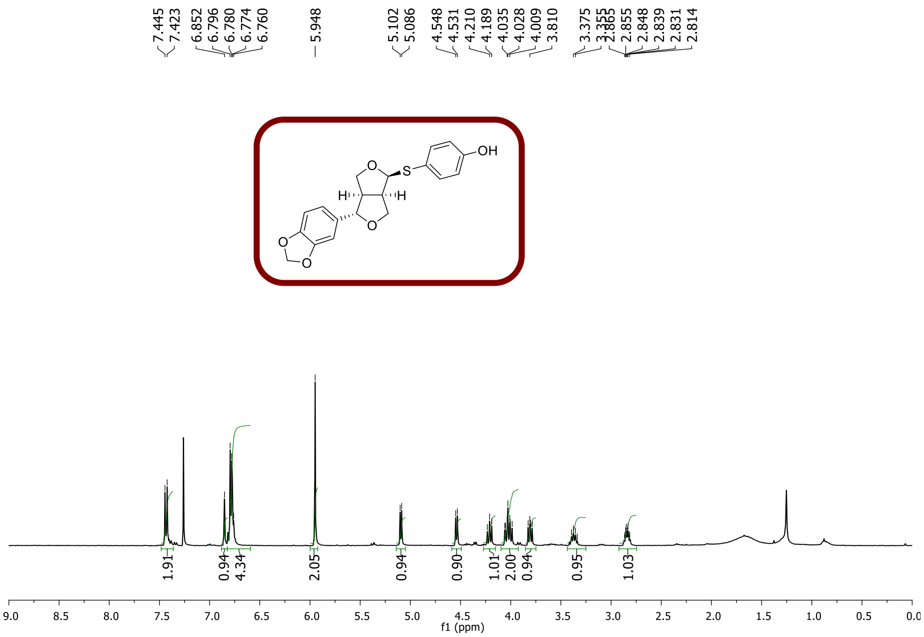

Figure S17. ^{1}H NMR spectrum of **2β-7f** ($CDCl_3$)

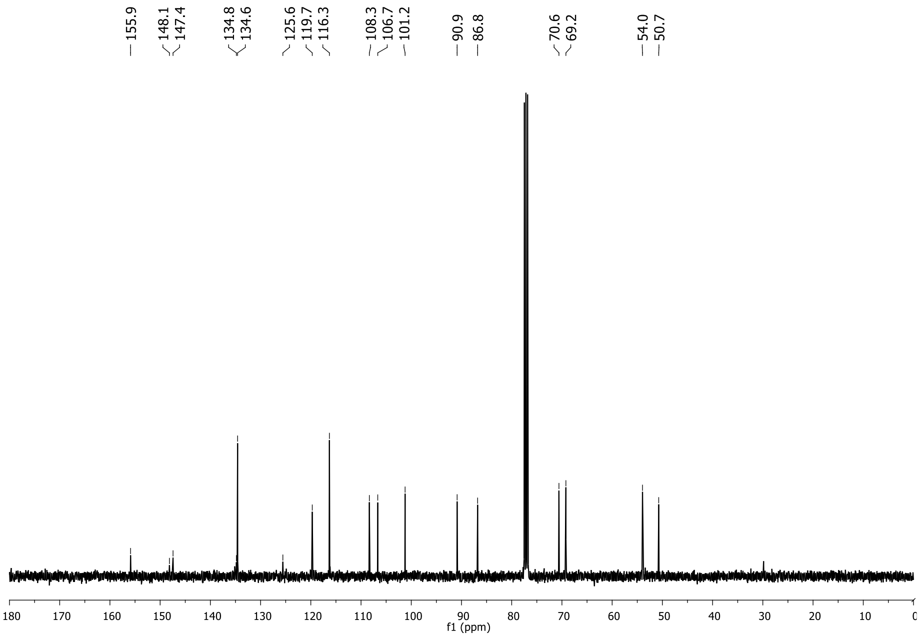

Figure S18. ^{13}C NMR spectrum of **2β-7f** ($CDCl_3$)

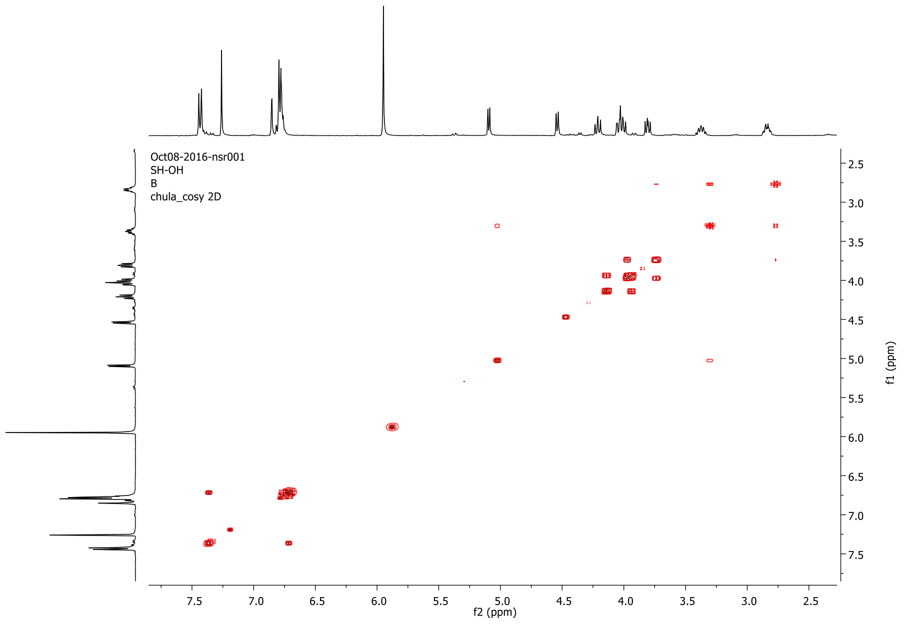

Figure S19. COSY spectrum of **2β-7f** ($CDCl_3$)

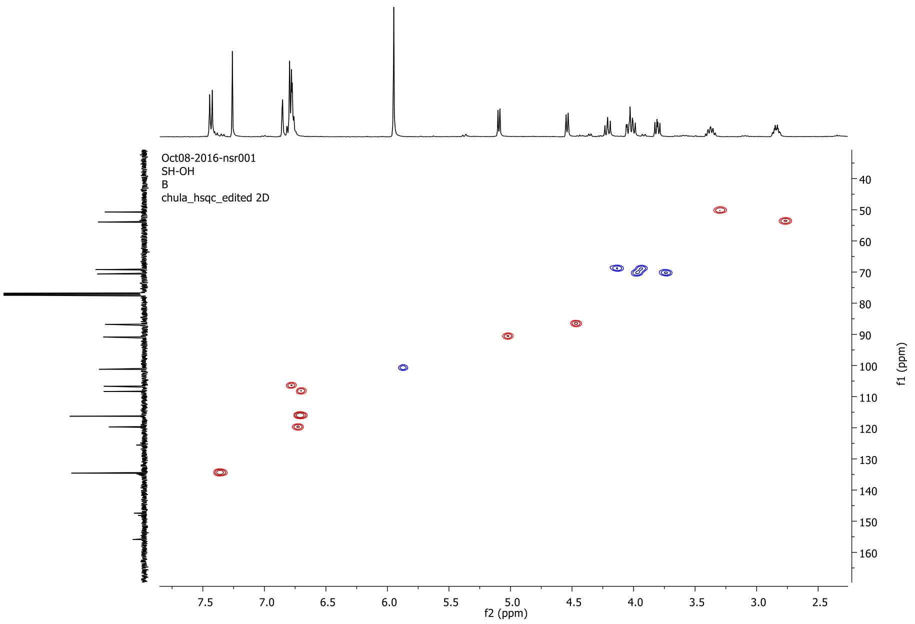

Figure S20. HSQC spectrum of **2β-7f** ($CDCl_3$)

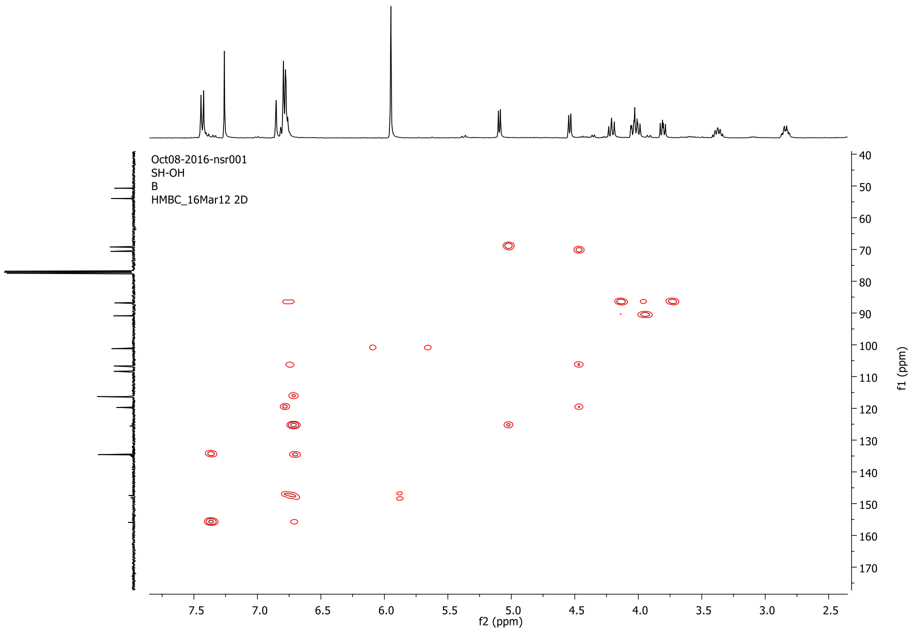

Figure S21. HMBC spectrum of **2β-7f** ($CDCl_3$)

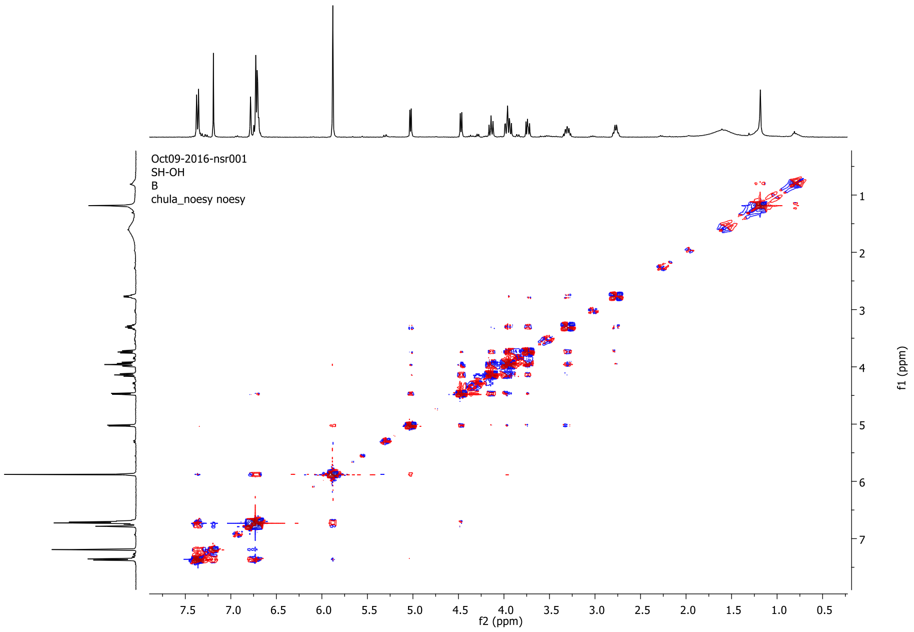

Figure S22. NOESY spectrum of **2β-7f** ($CDCl_3$)

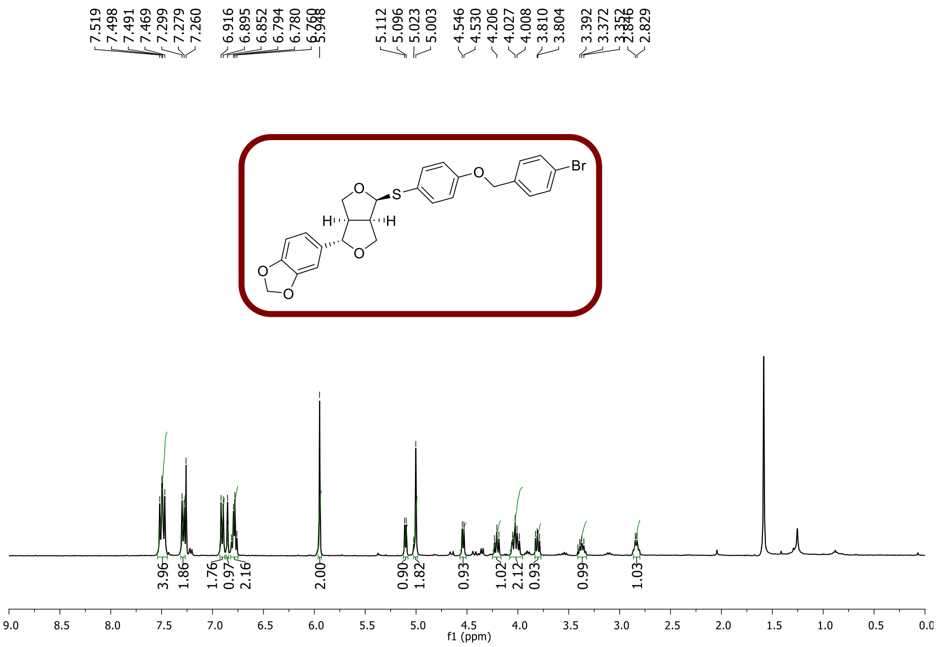

Figure S23. ^{1}H NMR spectrum of **2β-7fBr** ($CDCl_3$)

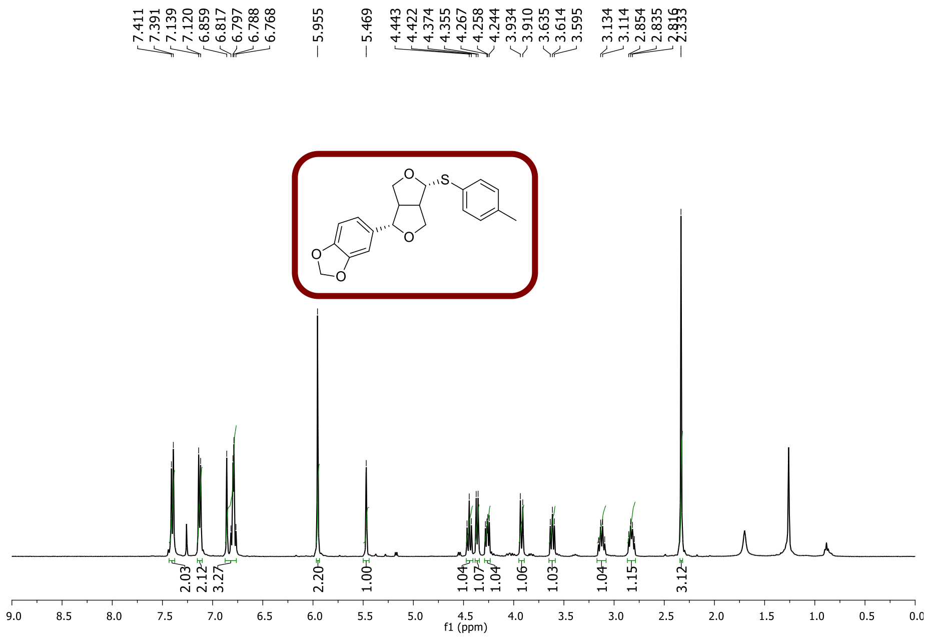

Figure S24. ^{1}H NMR spectrum of **2α-7g** ($CDCl_3$)

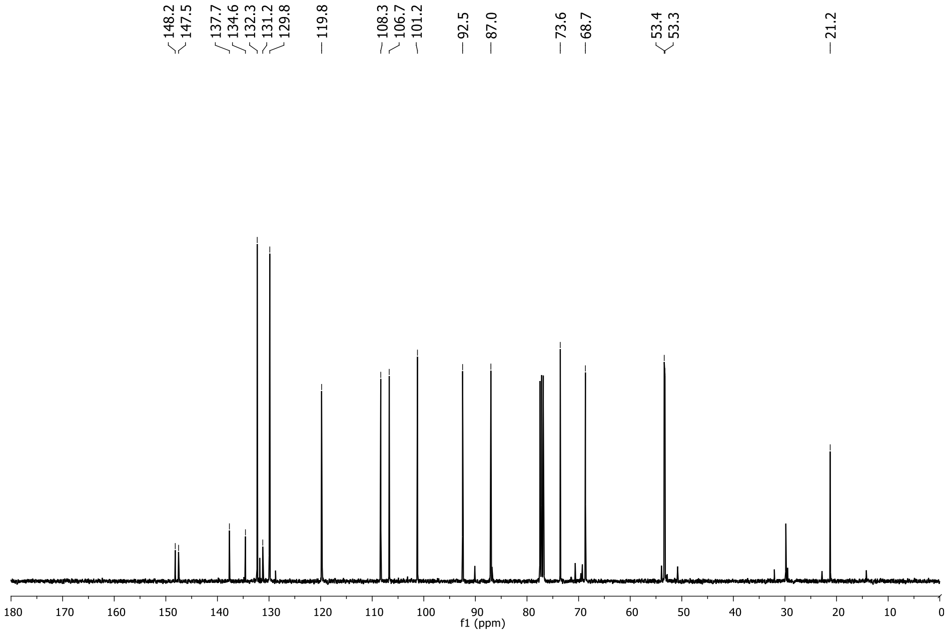

Figure S25. ^{13}C NMR spectrum of **2α-7g** ($CDCl_3$)

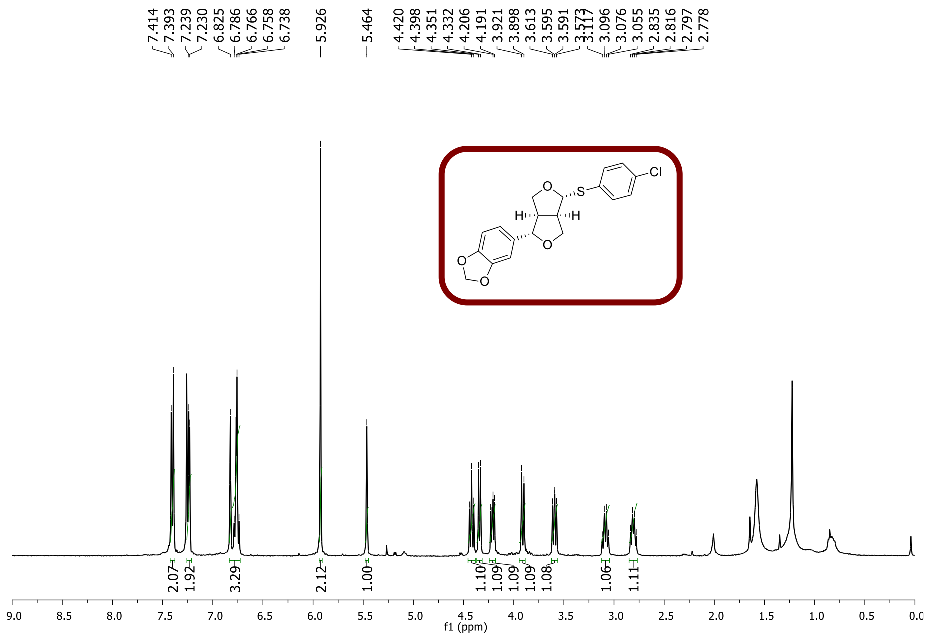

Figure S26. ^{1}H NMR spectrum of **2α-7h** ($CDCl_3$)

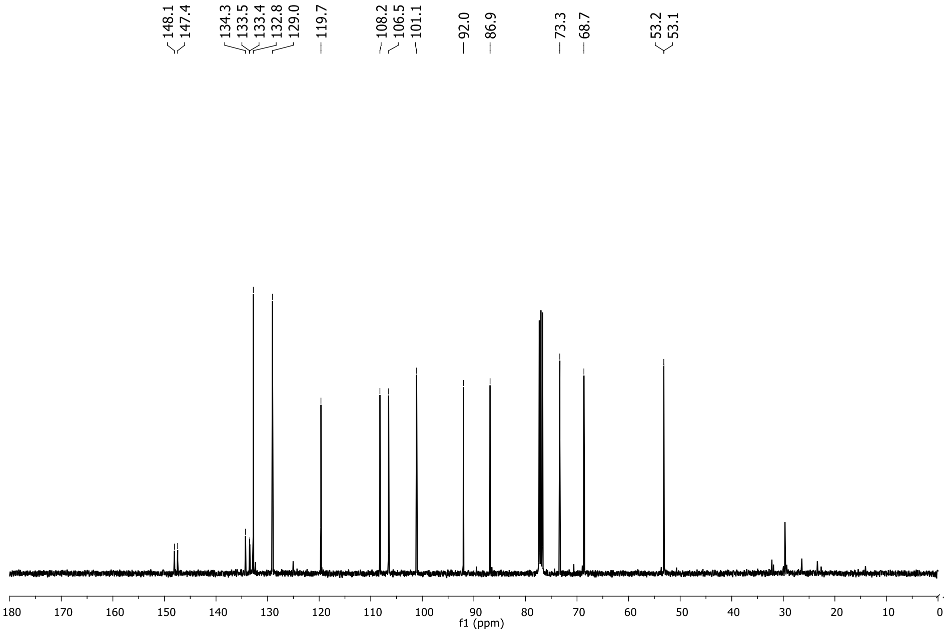

Figure S27. ^{13}C NMR spectrum of **2α-7h** ($CDCl_3$)

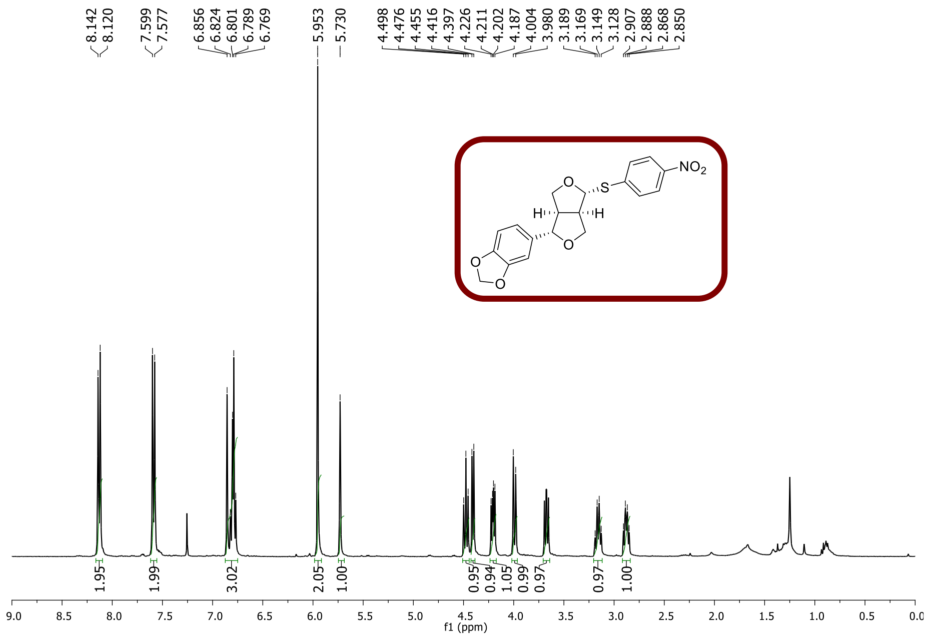

Figure S28. ^{1}H NMR spectrum of **2α-7i** ($CDCl_3$)

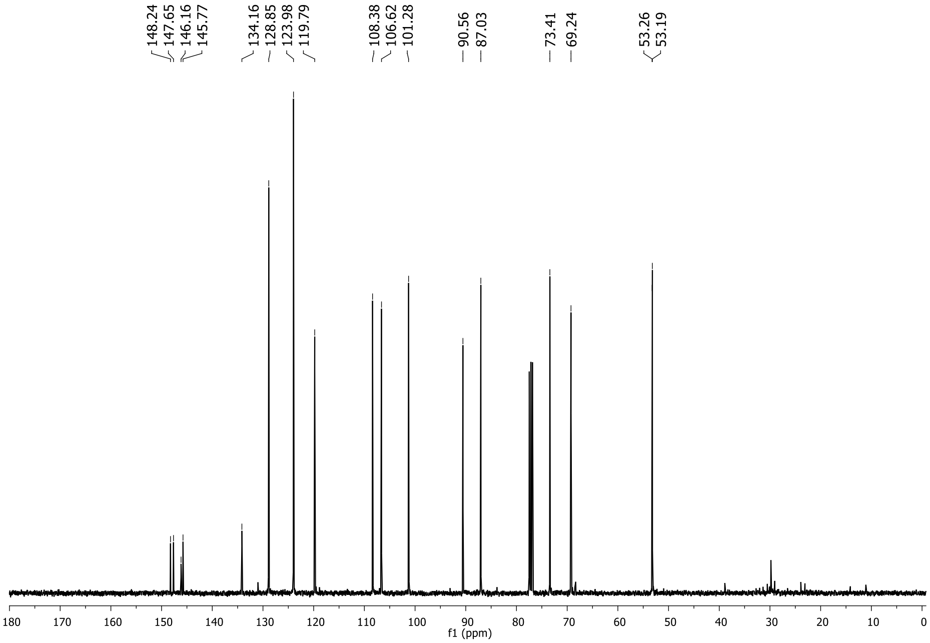

Figure S29. ^{13}C NMR spectrum of **2α-7i** ($CDCl_3$)

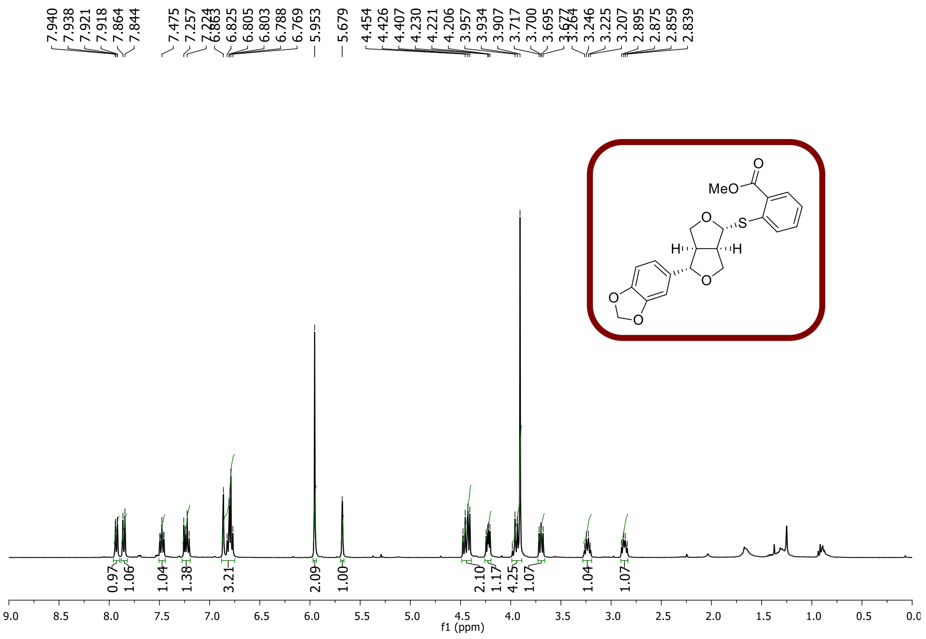

Figure S30. ^{1}H NMR spectrum of **2α-7j** ($CDCl_3$)

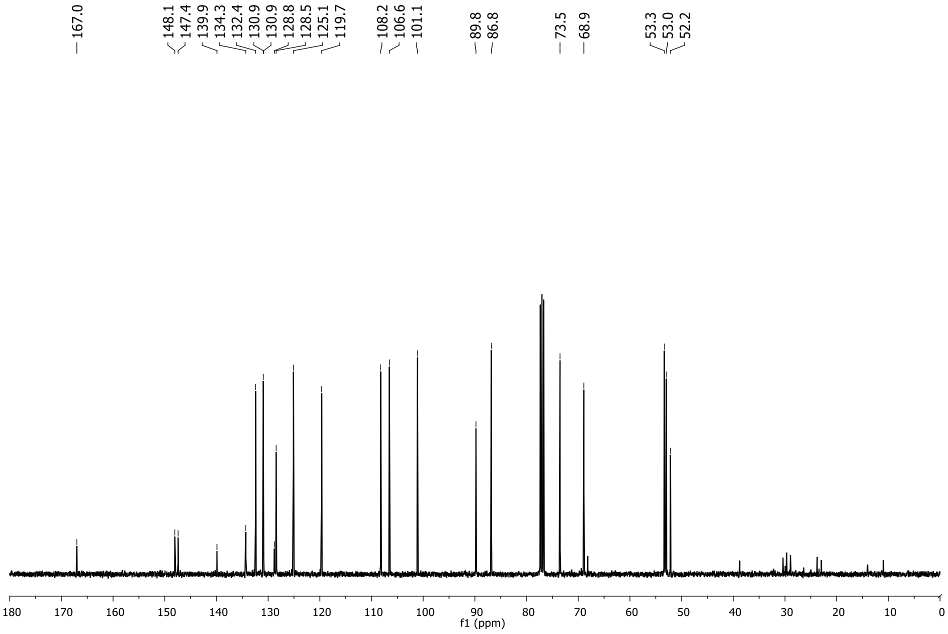

Figure S31. ^{13}C NMR spectrum of **2α-7j** ($CDCl_3$)

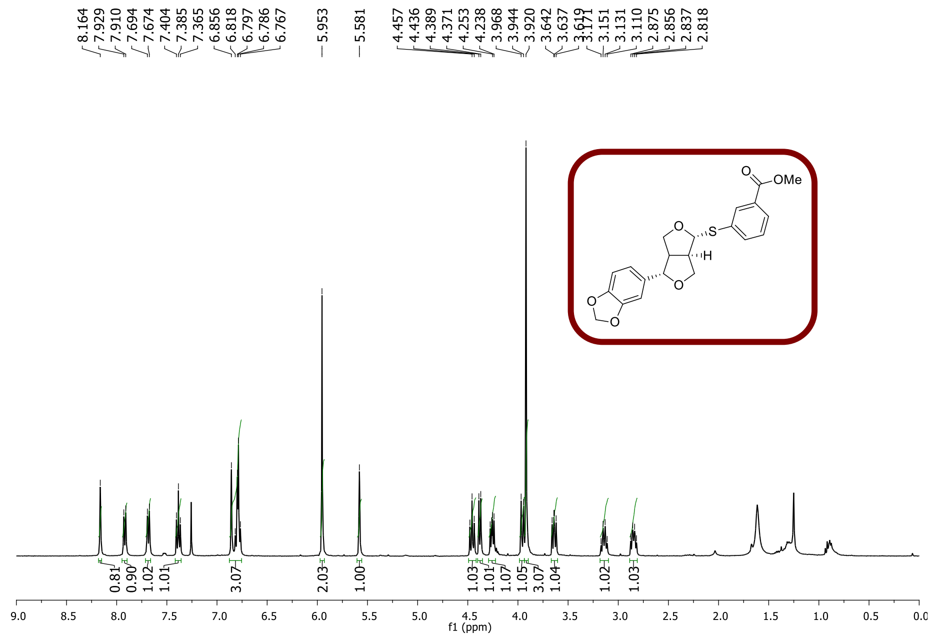

Figure S32. ^{1}H NMR spectrum of **2α-7k** ($CDCl_3$)

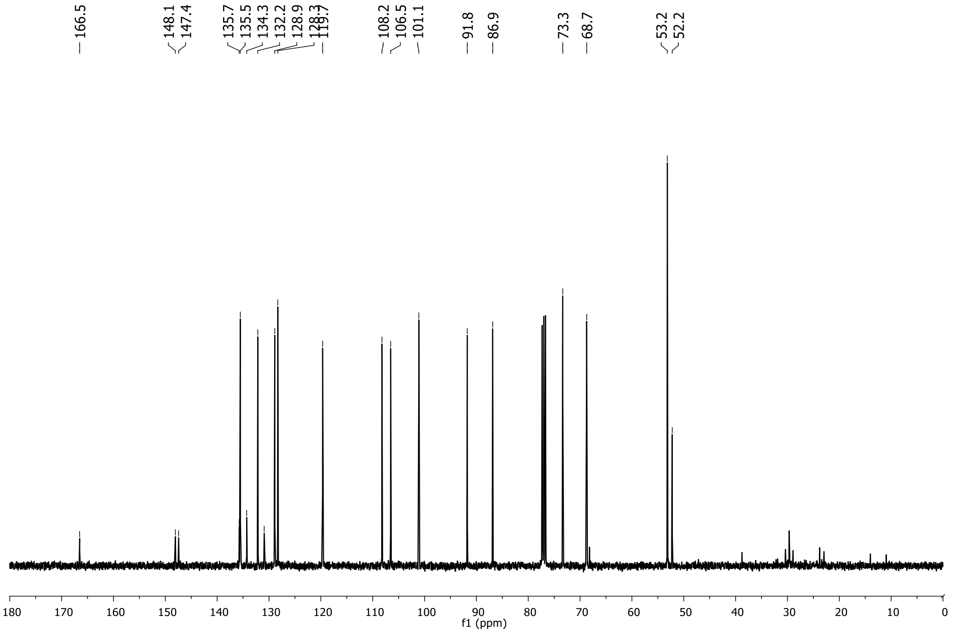

Figure S33. ^{13}C NMR spectrum of **2α-7k** ($CDCl_3$)

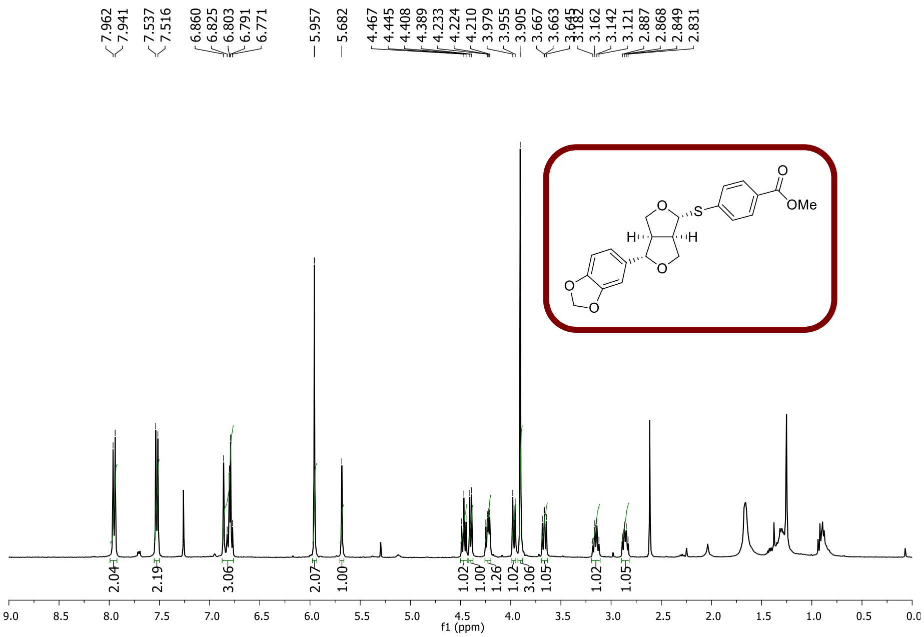

Figure S34. ^{1}H NMR spectrum of **2α-7l** (CDCl$_3$)

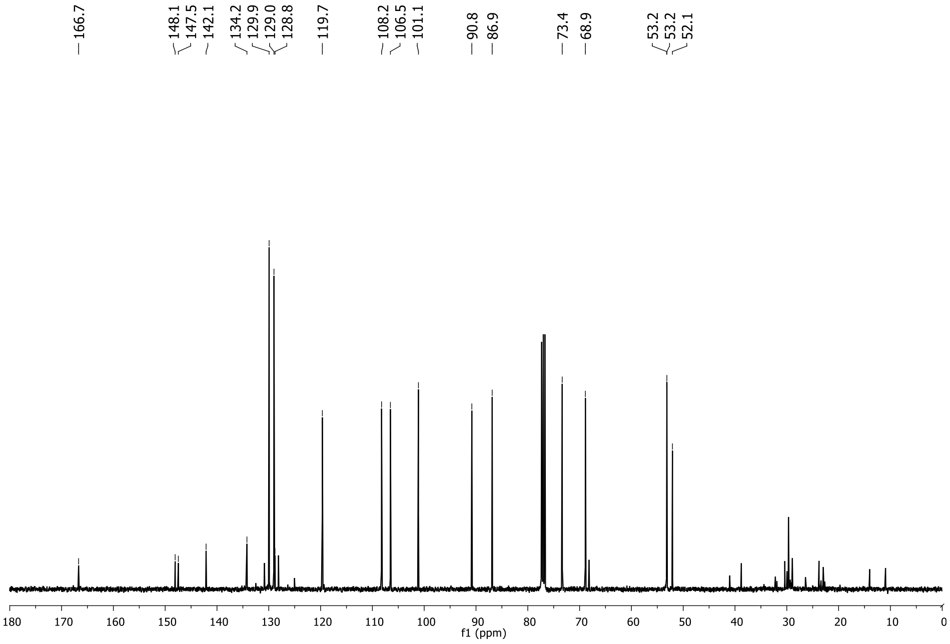

Figure S35. ^{13}C NMR spectrum of **2α-7l** (CDCl$_3$)

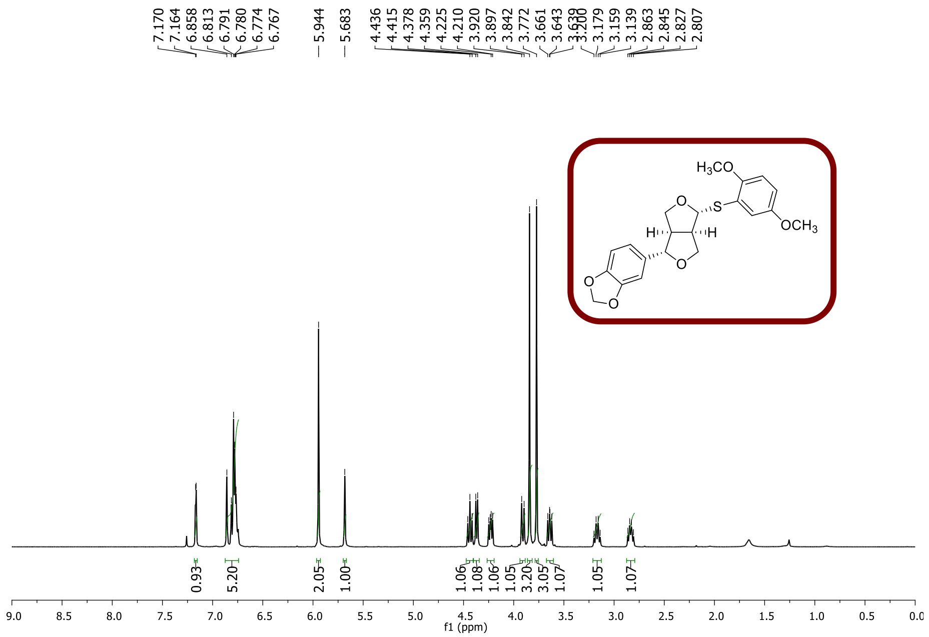

Figure S36. ^{1}H NMR spectrum of **2α-7m** ($CDCl_3$)

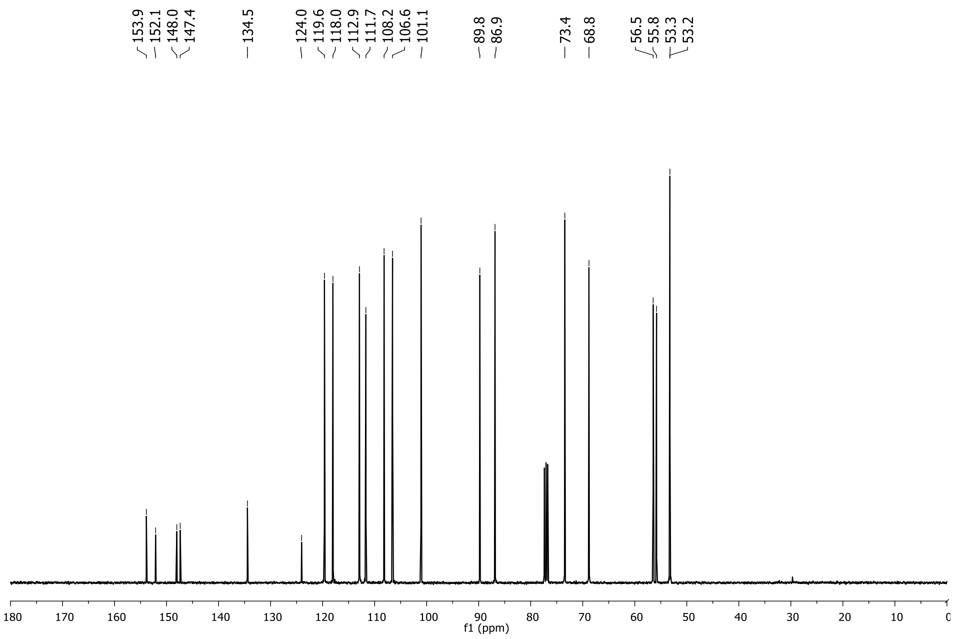

Figure S37. ^{13}C NMR spectrum of **2α-7m** ($CDCl_3$)

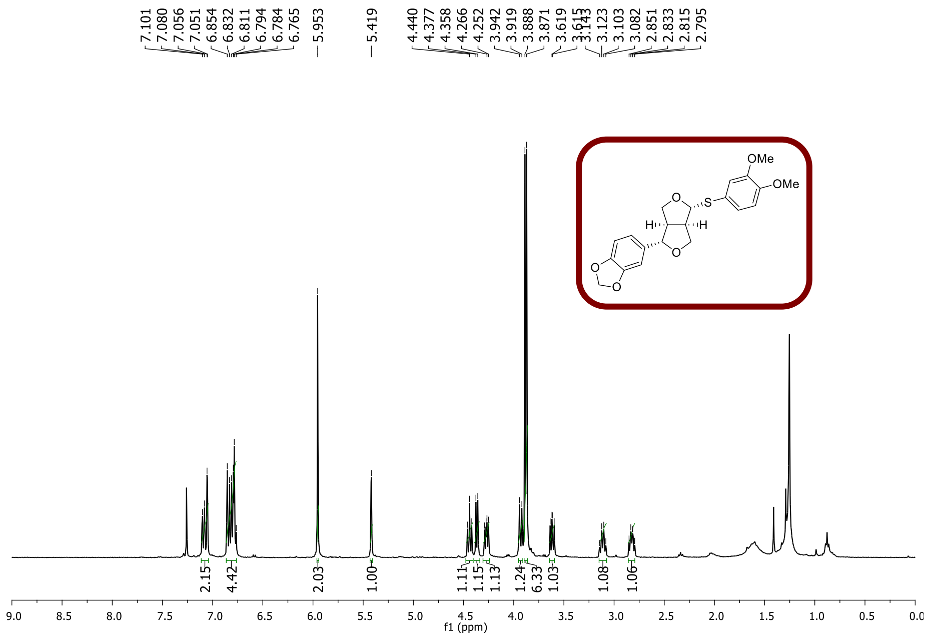

Figure S38. ^{1}H NMR spectrum of **2α-7n** ($CDCl_3$)

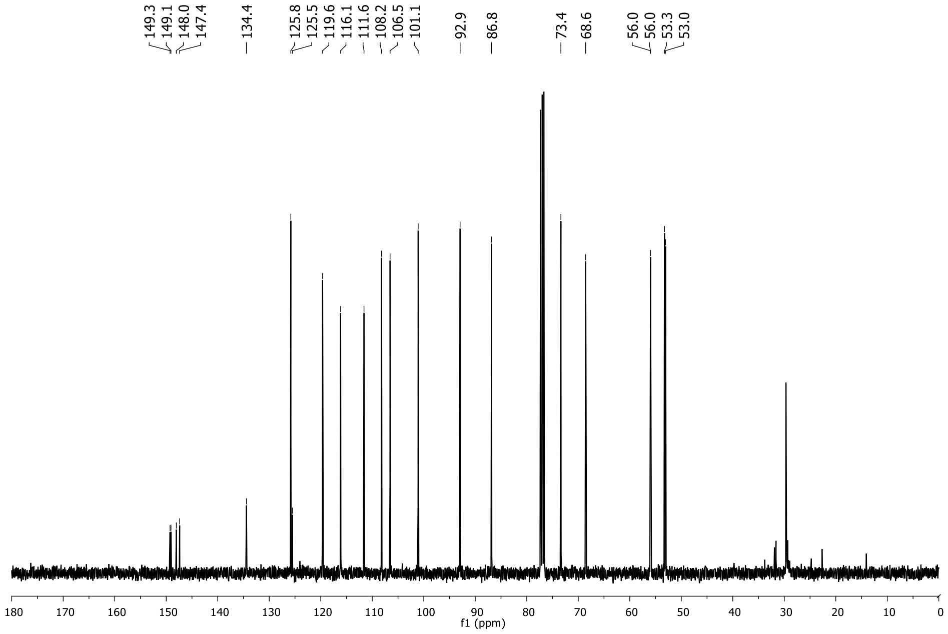

Figure S39. ^{13}C NMR spectrum of **2α-7n** ($CDCl_3$)

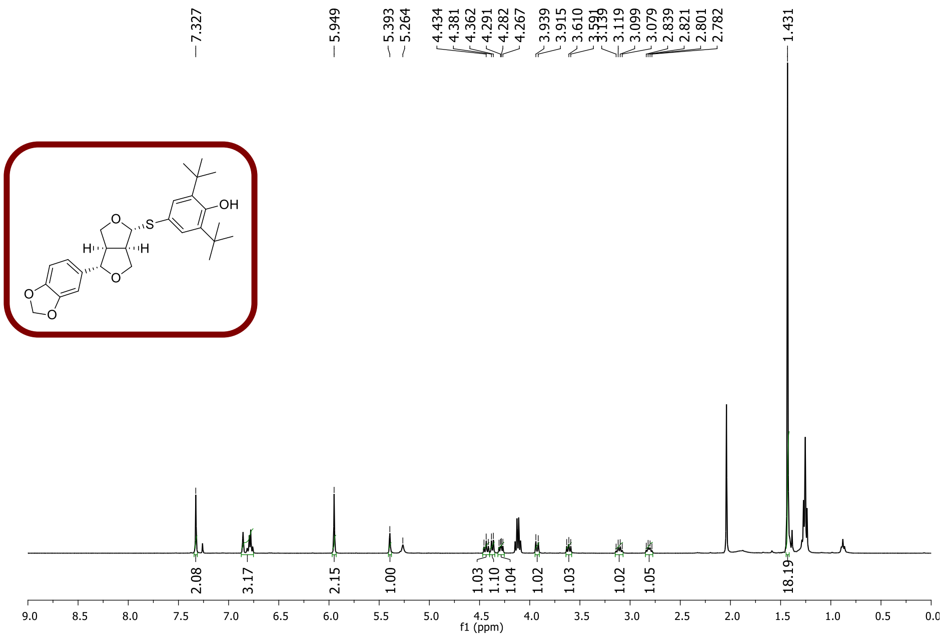

Figure S40. ^{1}H NMR spectrum of **2α-7o** (CDCl$_3$)

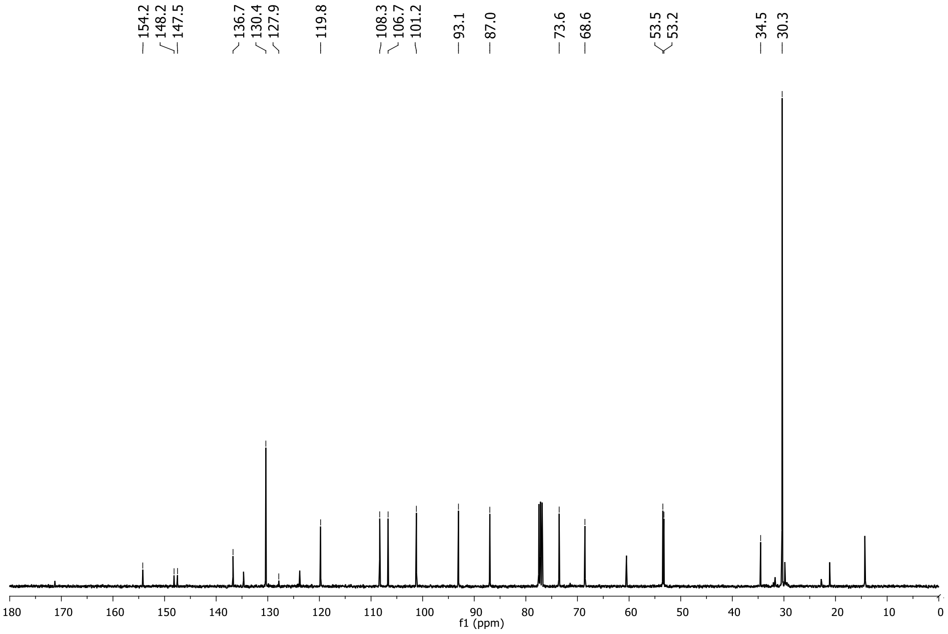

Figure S41. ^{13}C NMR spectrum of **2α-7o** (CDCl$_3$)

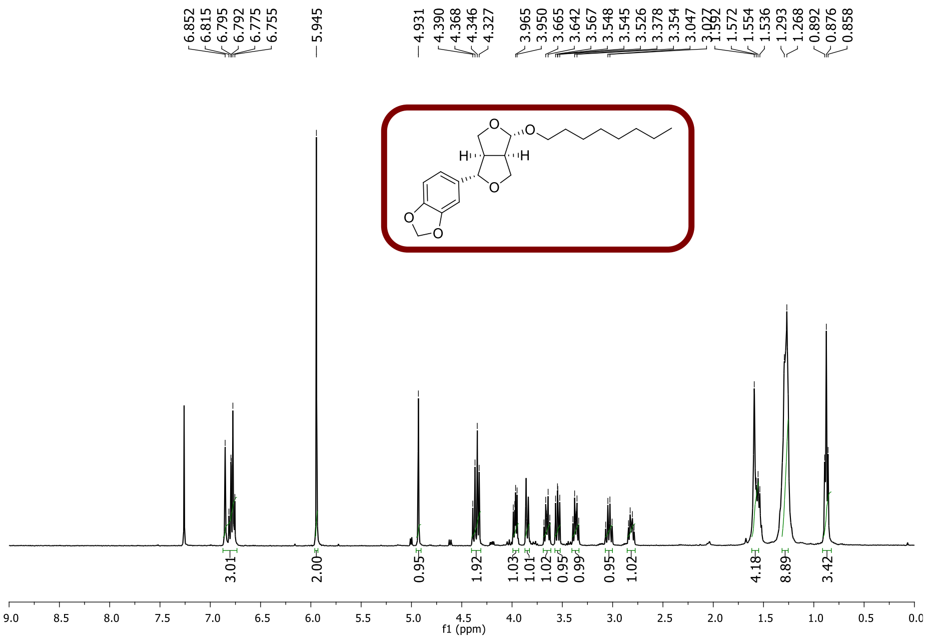

Figure S42. ^{1}H NMR spectrum of **2α-9b** ($CDCl_3$)

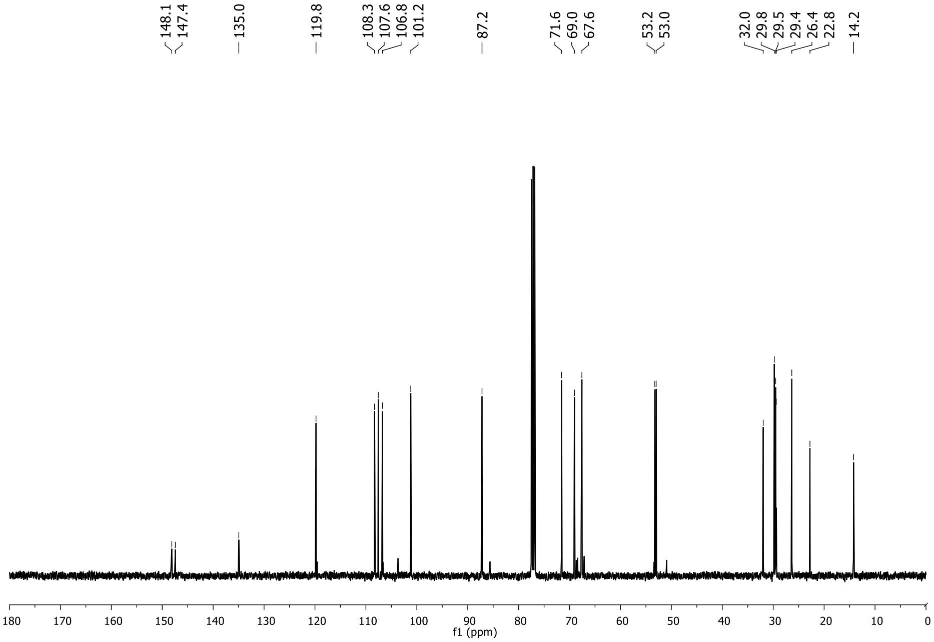

Figure S43. ^{13}C NMR spectrum of **2α-9b** ($CDCl_3$)

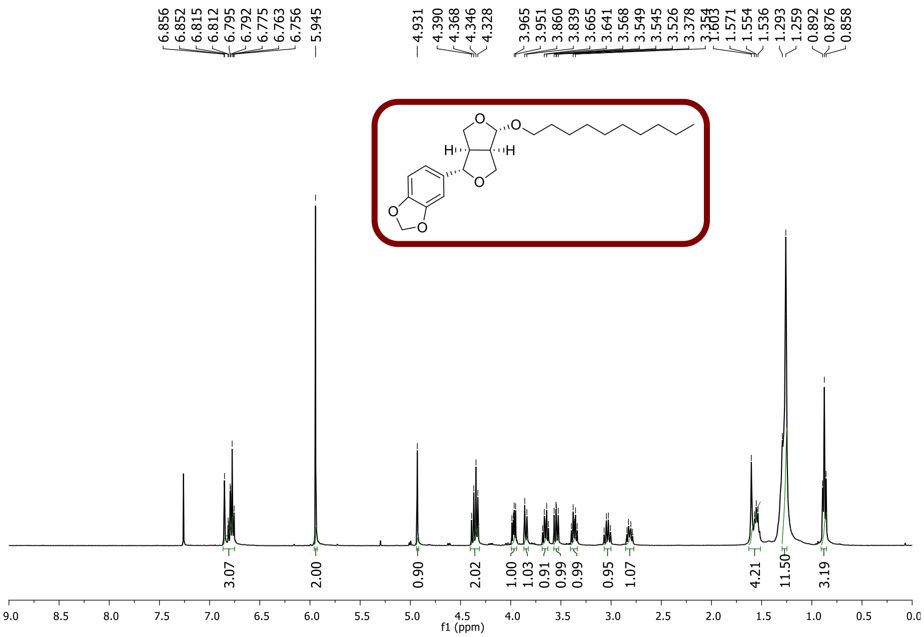

Figure S44. ^{1}H NMR spectrum of **2α-9c** ($CDCl_3$)

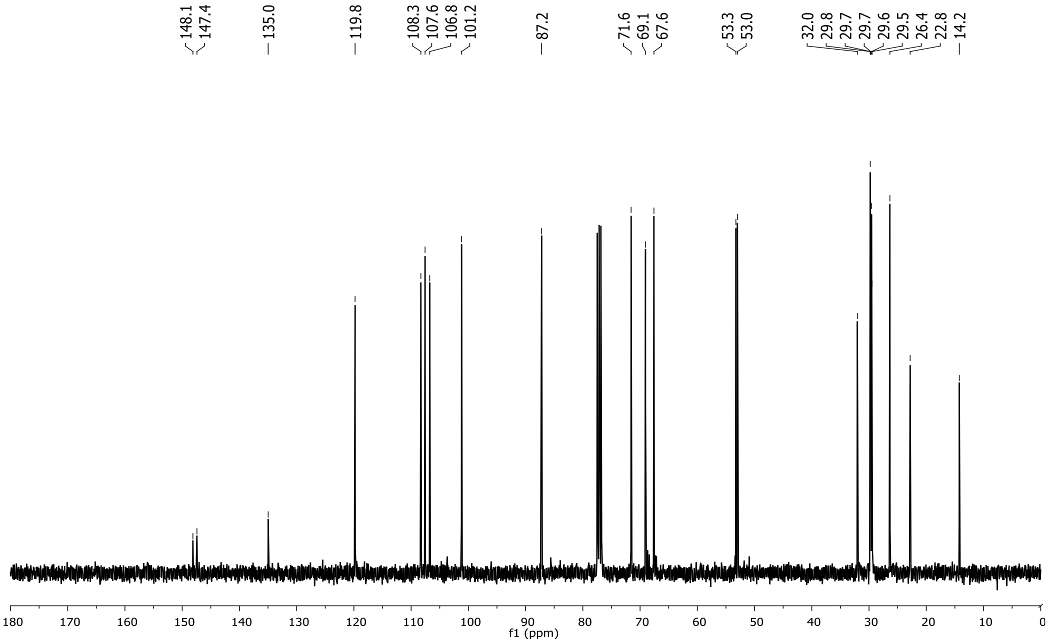

Figure S45. ^{13}C NMR spectrum of **2α-9c** ($CDCl_3$)

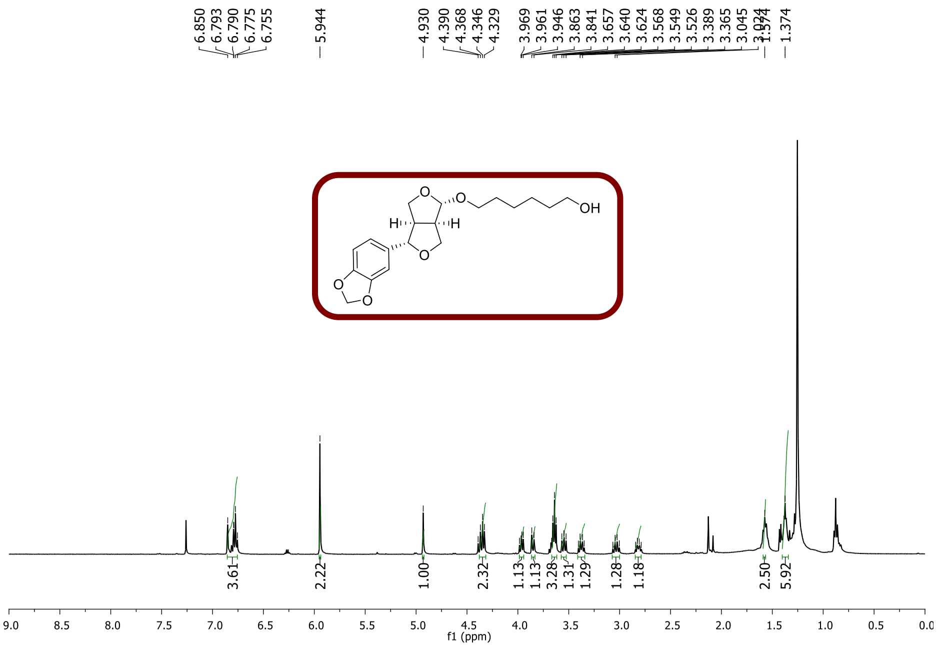

Figure S46. ^{1}H NMR spectrum of **2α-9d** ($CDCl_3$)

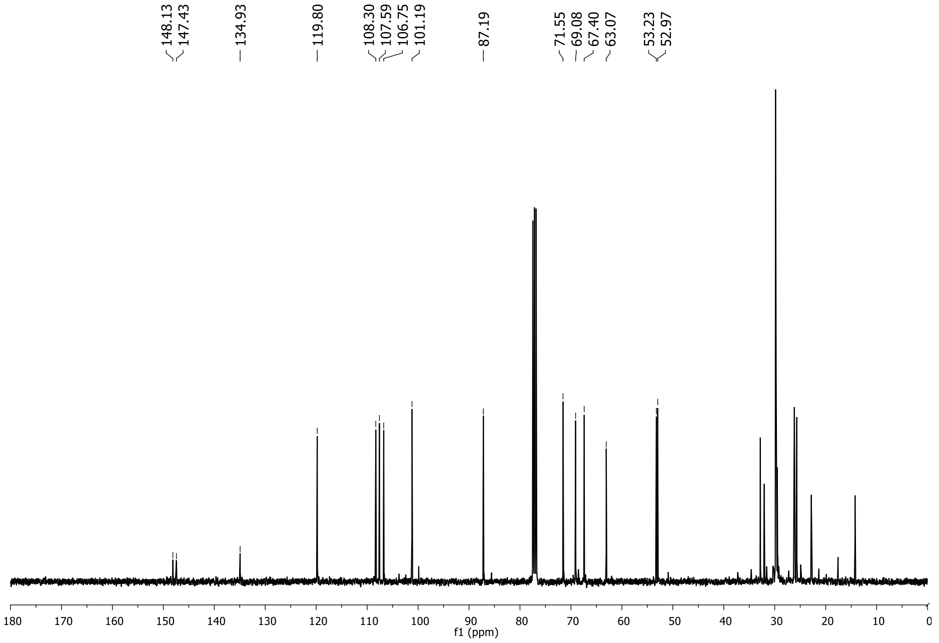

Figure S47. ^{13}C NMR spectrum of **2α-9d** ($CDCl_3$)

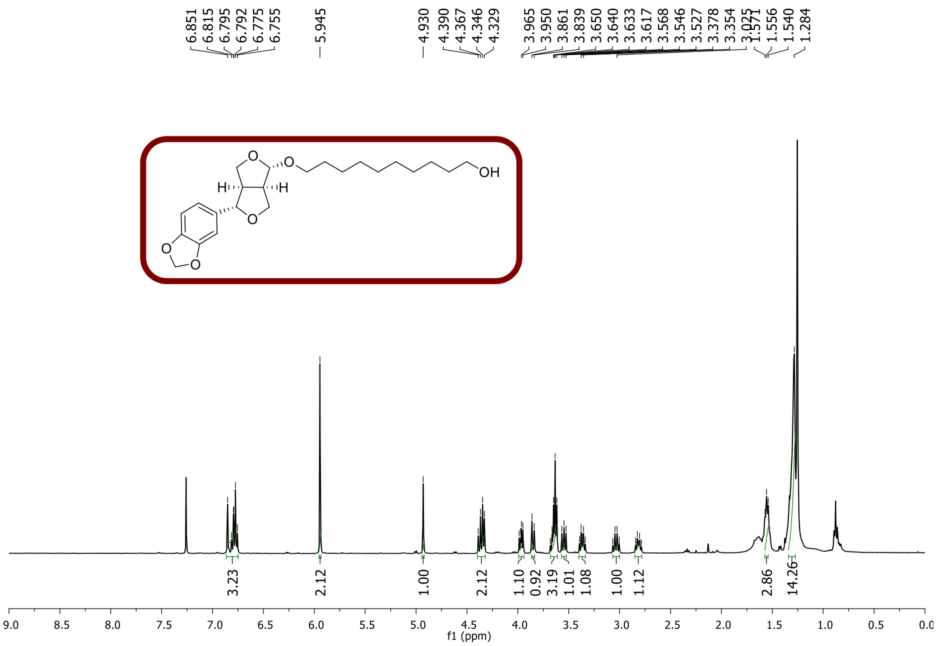

Figure S48. ^{1}H NMR spectrum of **2β-9e** ($CDCl_3$)

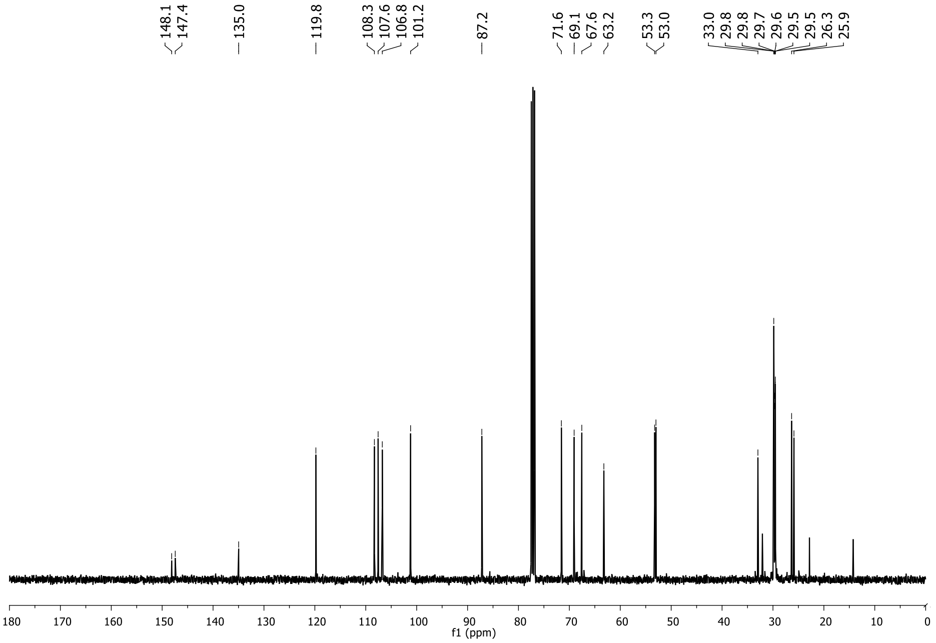

Figure S49. ^{13}C NMR spectrum of **2β-9e** ($CDCl_3$)

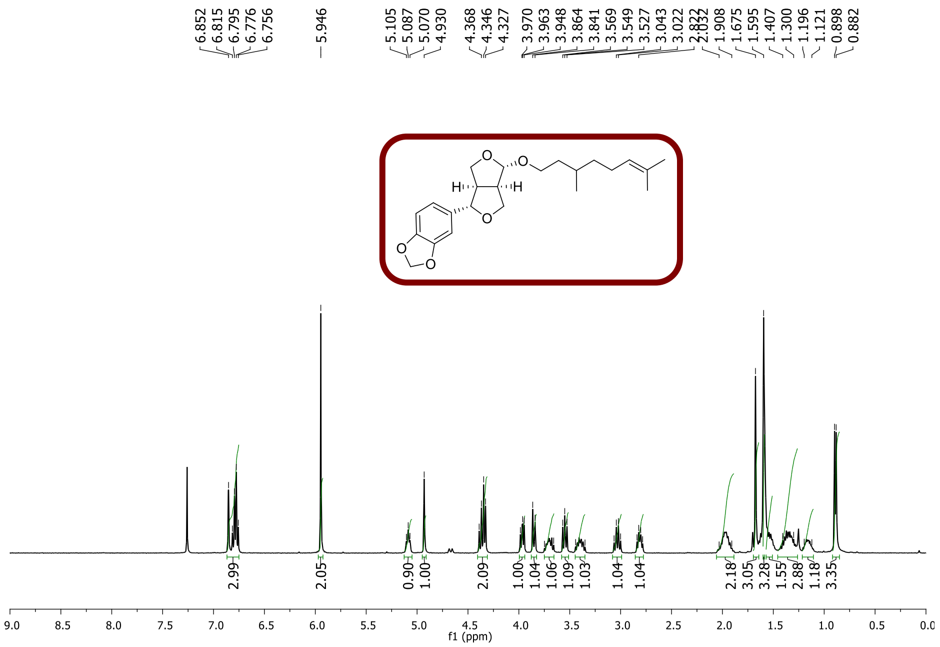

Figure S50. ^{1}H NMR spectrum of **2α-9f** ($CDCl_3$)

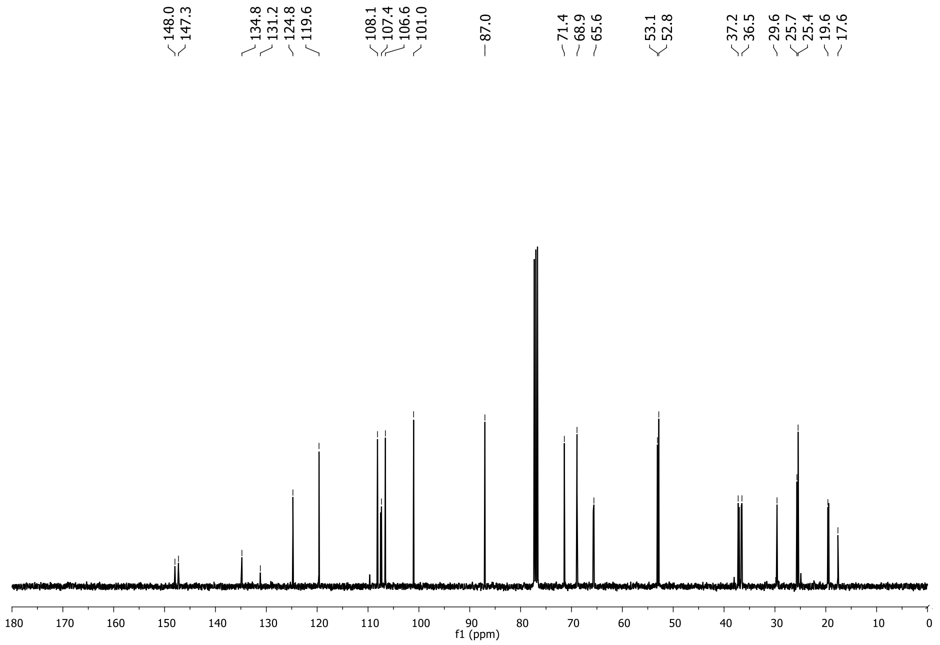

Figure S51. ^{13}C NMR spectrum of **2α-9f** ($CDCl_3$)

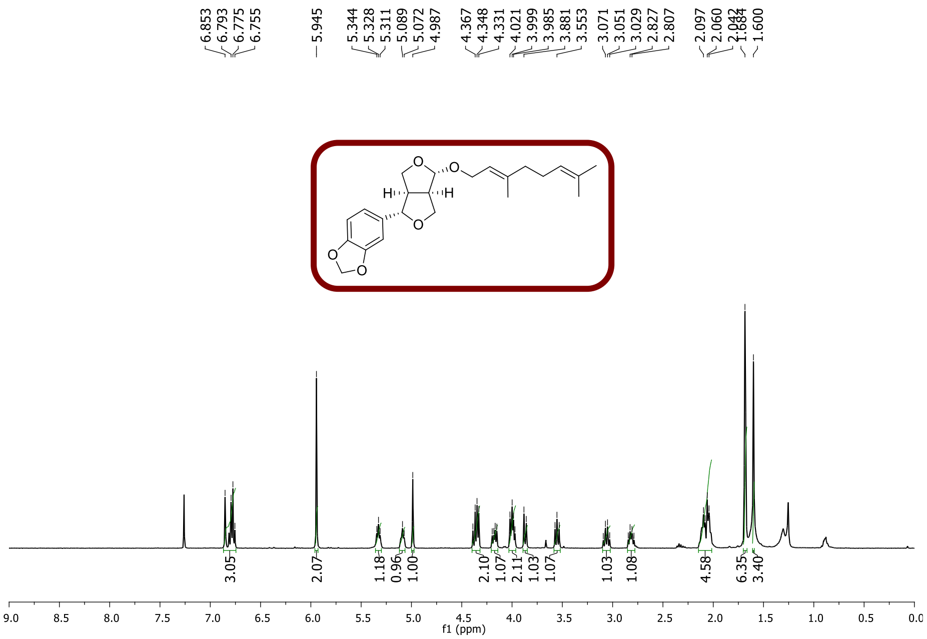

Figure S52. ^{1}H NMR spectrum of **2α-9g** ($CDCl_3$)

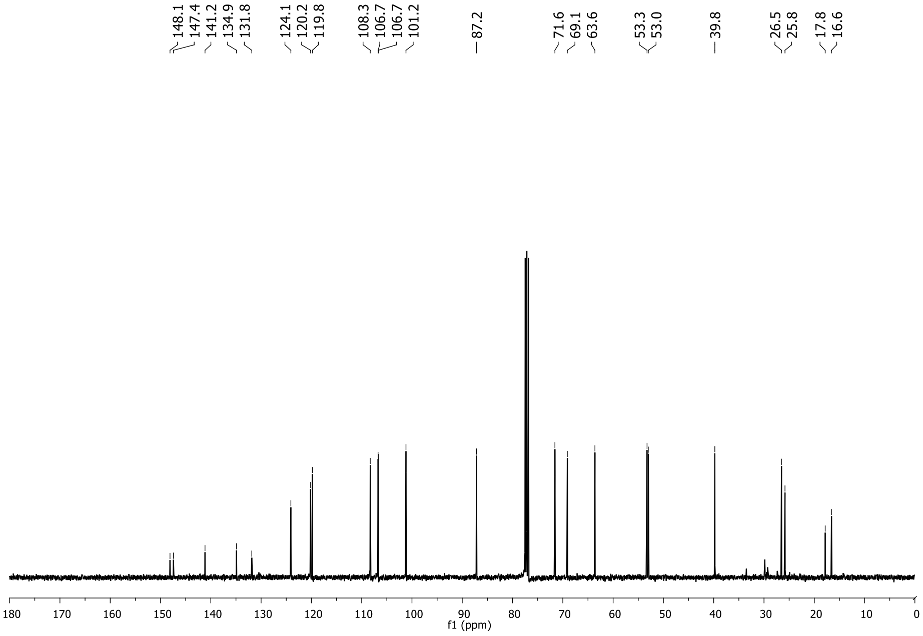

Figure S53. ^{13}C NMR spectrum of **2α-9g** ($CDCl_3$)

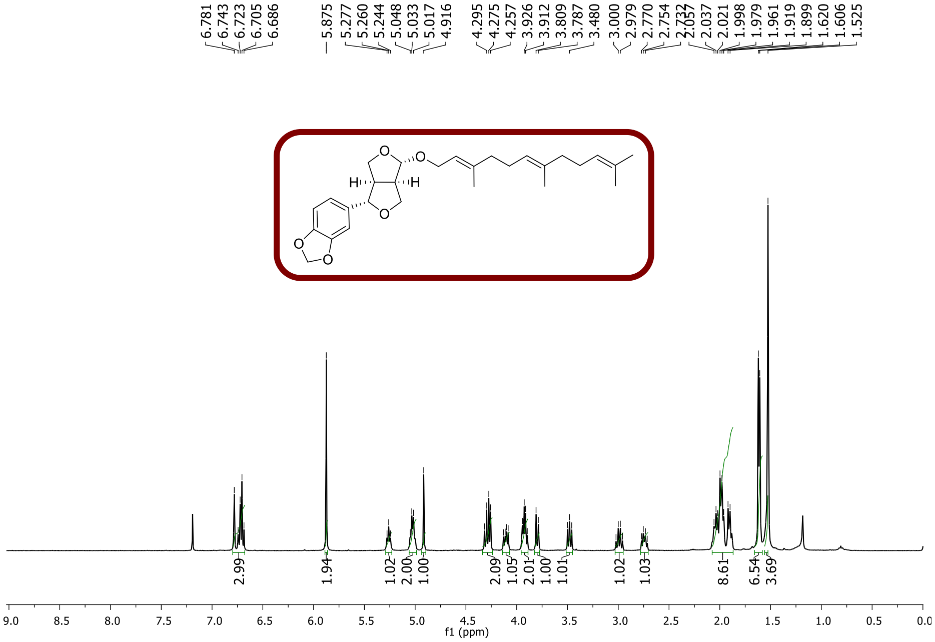

Figure S54. ^{1}H NMR spectrum of **2α-9h** ($CDCl_3$)

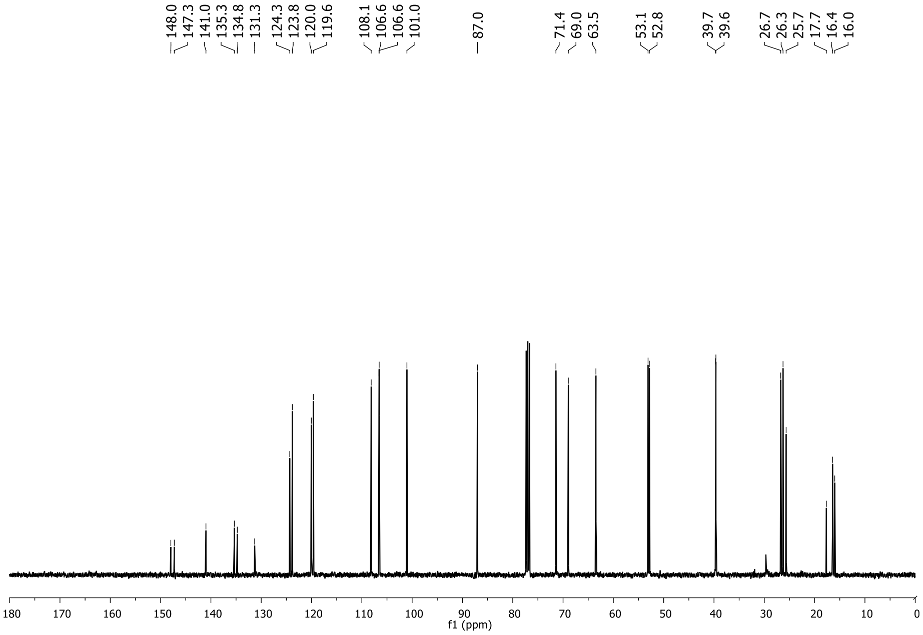

Figure S55. ^{13}C NMR spectrum of **2α-9h** ($CDCl_3$)

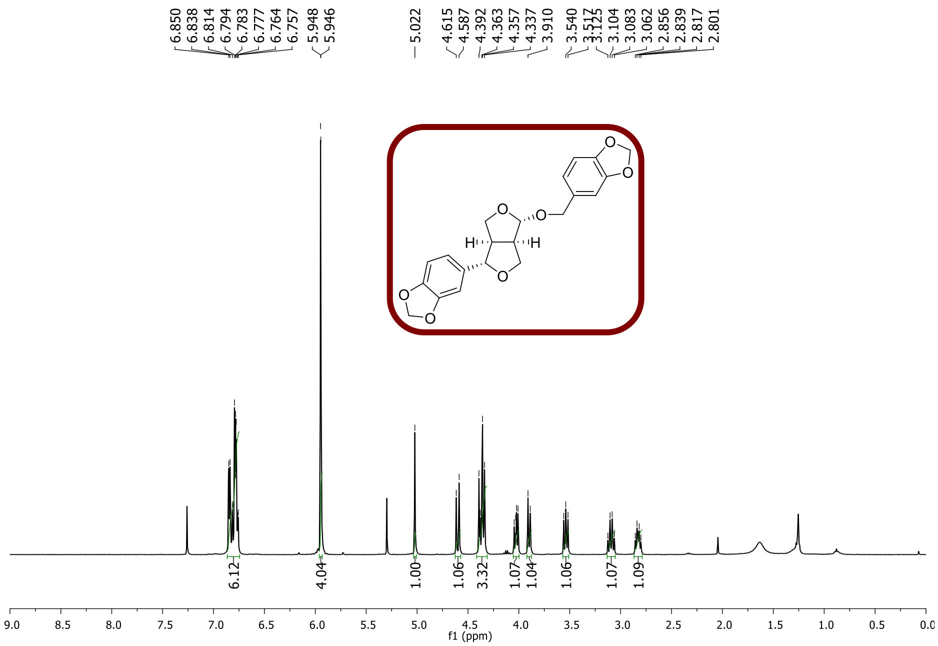

Figure S56. ^{1}H NMR spectrum of **2α-9i** ($CDCl_3$)

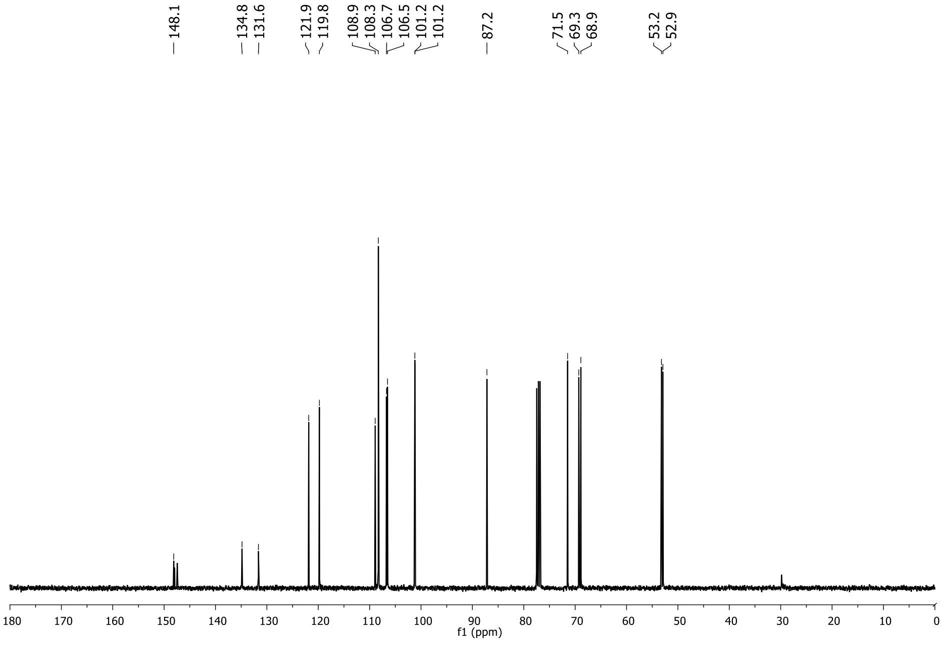

Figure S57. ^{13}C NMR spectrum of **2α-9i** ($CDCl_3$)

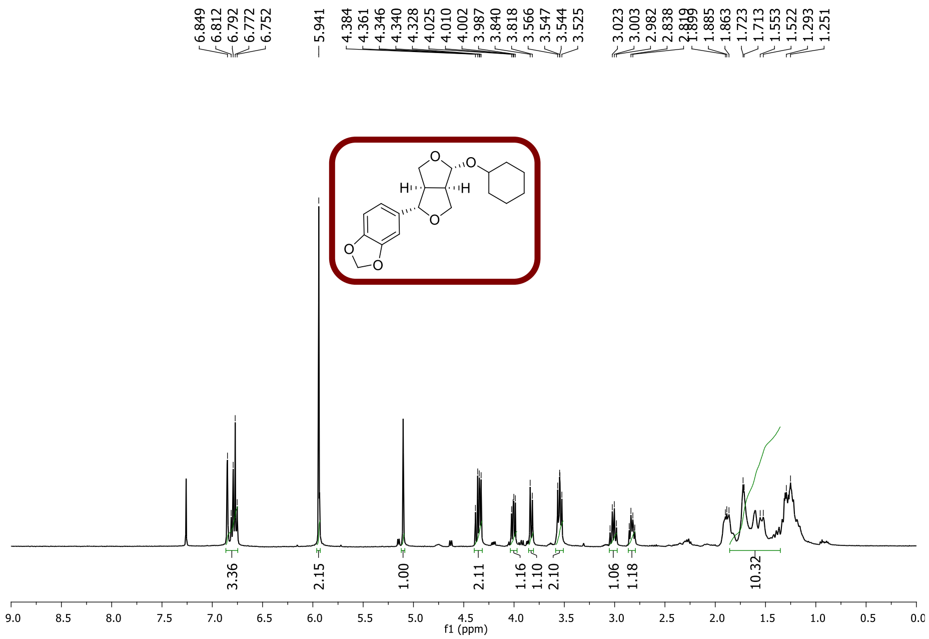

Figure S58. ^{1}H NMR spectrum of **2α-9j** ($CDCl_3$)

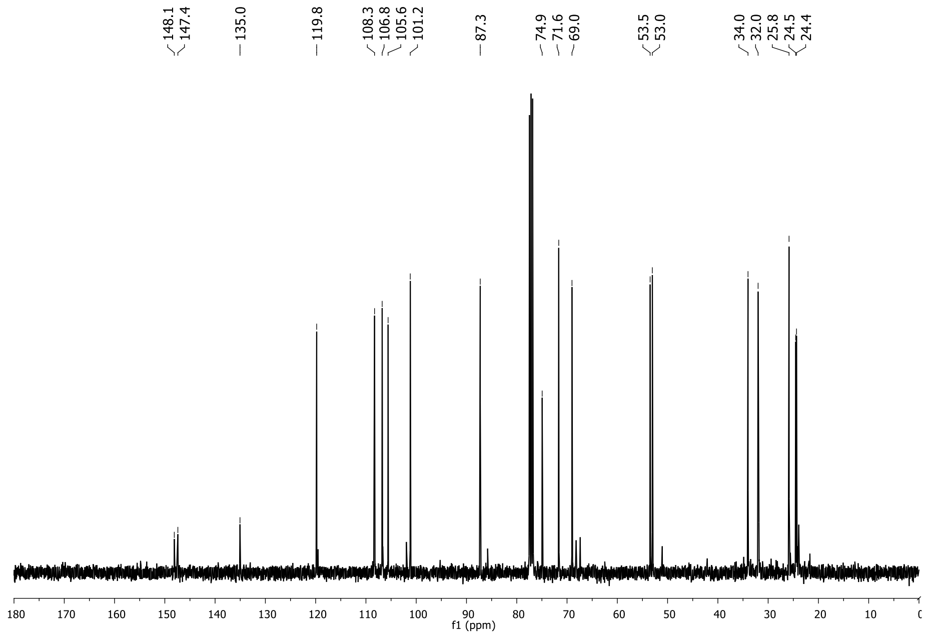

Figure S59. ^{13}C NMR spectrum of **2α-9j** ($CDCl_3$)

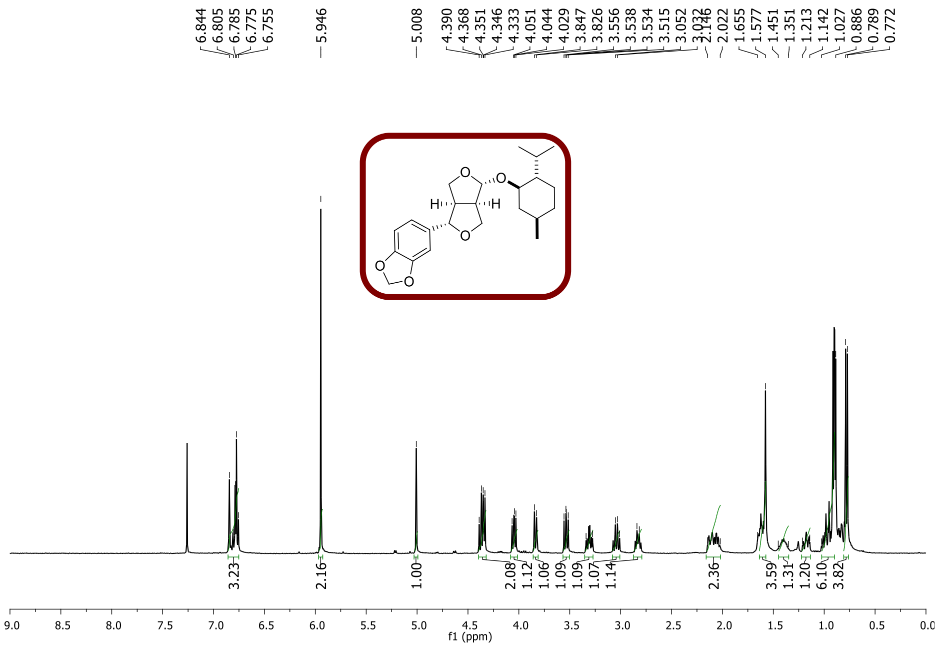

Figure S60. ^{1}H NMR spectrum of **2α-9k** ($CDCl_3$)

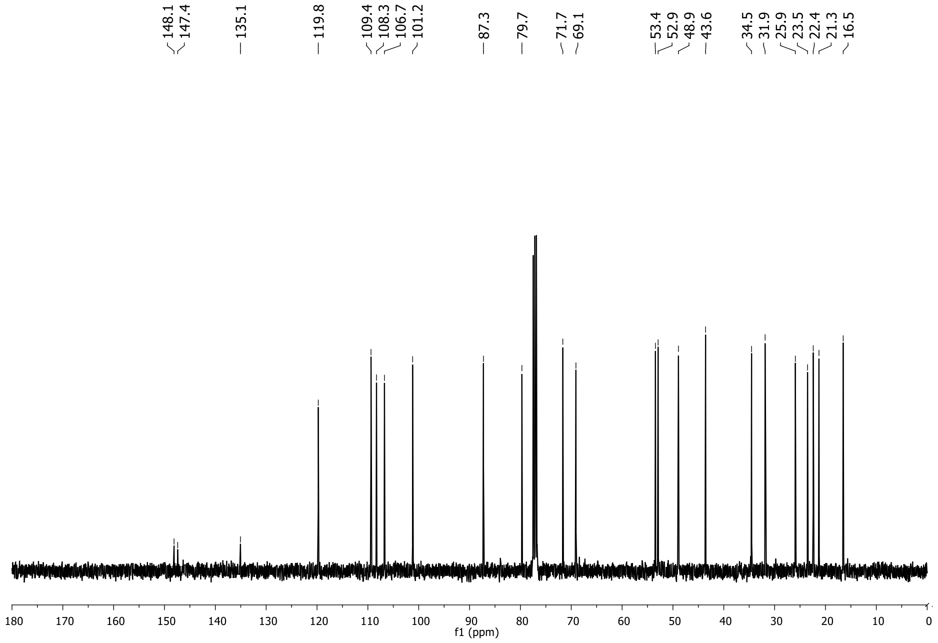

Figure S61. ^{13}C NMR spectrum of **2α-9k** ($CDCl_3$)

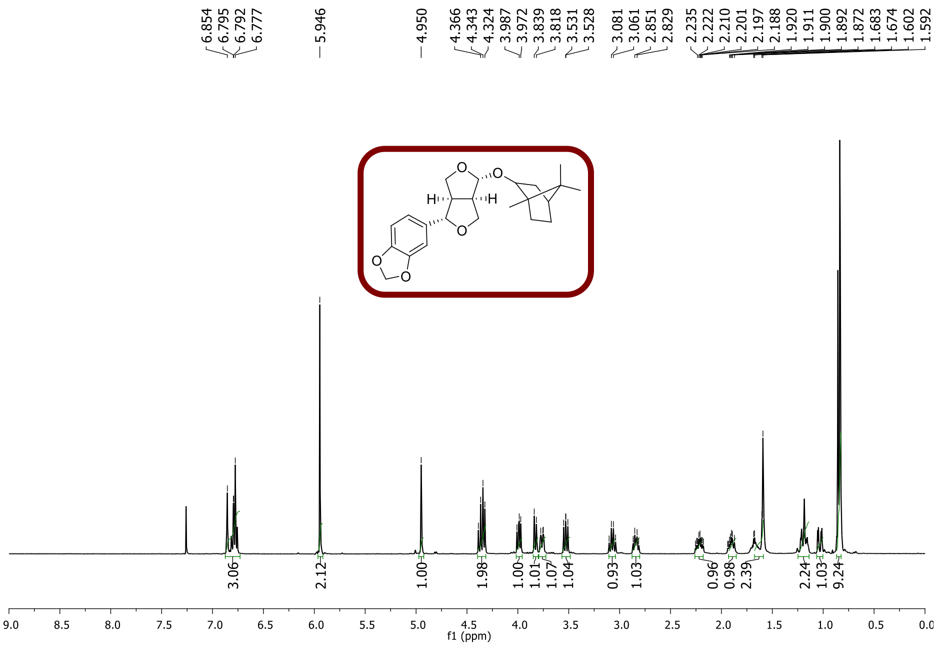

Figure S62. ^{1}H NMR spectrum of **2α-9l** ($CDCl_3$)

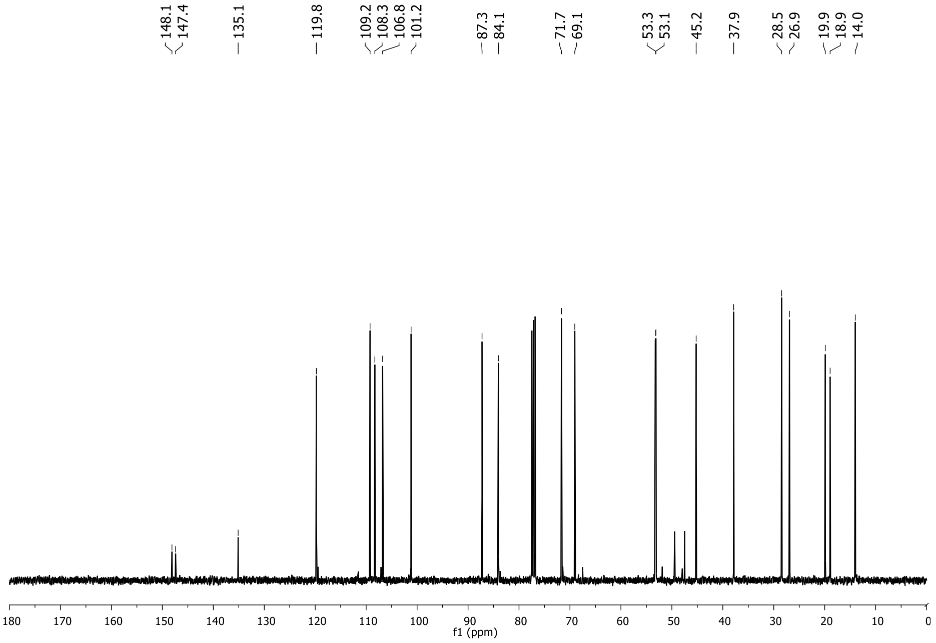

Figure S63. ^{13}C NMR spectrum of **2α-9l** ($CDCl_3$)

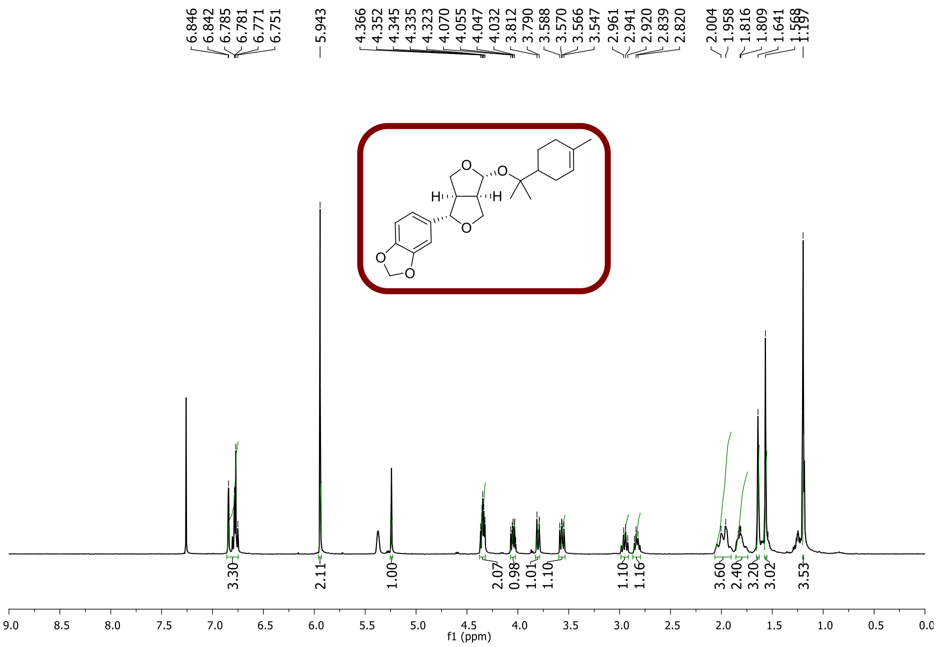

Figure S64. ^{1}H NMR spectrum of **2α-9m** ($CDCl_3$)

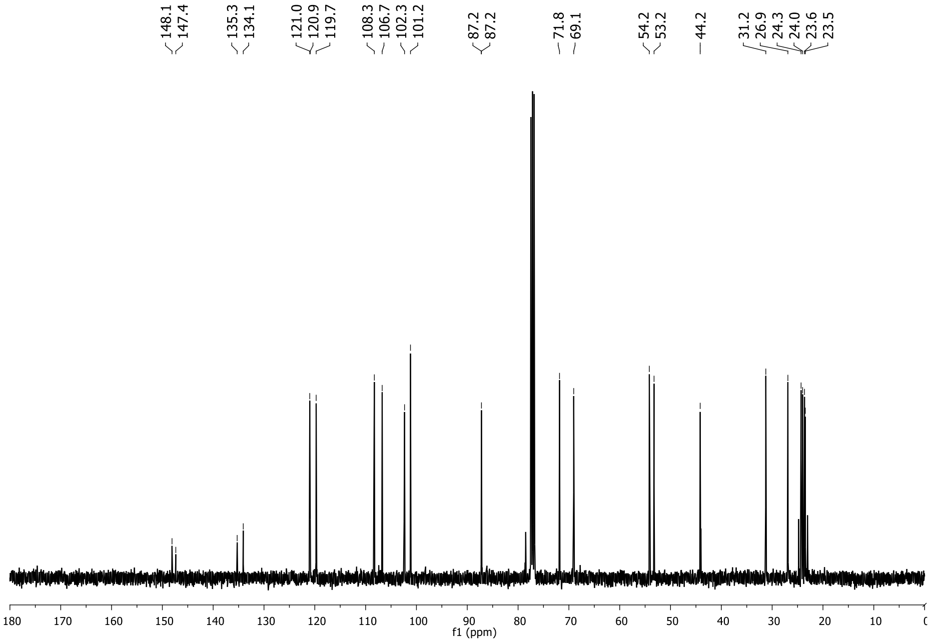

Figure S65. ^{13}C NMR spectrum of **2α-9m** ($CDCl_3$)

Table S2. Crystal data and refinement parameters of compounds **2α-7f** and **2β-7fBr**.

	**2α-7f**	**2β-7fBr**
Crystal habit	Thin plate, colorless	Thin plate, colorless
Crystal size (mm^3)	0.01×0.24×0.34	0.01×0.18×0.20
Empirical formula	$C_{19}\ H_{18}\ O_5\ S$	$C_{26}\ H_{23}\ Br\ O_5\ S$
FW	358.40	527.41
Crystal system	Monoclinic	Triclinic
Space group	$P2_1$ (no. 4)	$P1$ (no. 1)
a [Å]	6.1485(2)	6.7501(13)
b [Å]	7.6475(2)	8.8663(18)
c [Å]	17.9288(4)	20.864(4)
α [º]	90	95.858(5)
β [º]	90.711(1)	94.023(5)
γ [º]	90	111.089(5)
V [Å^3]	842.96(4)	1151.3(4)
Z	2	2
ρ_{calcd} [Mg m^{-3}]	1.412	1.521
μ [mm^{-1}]	1.948	1.912
F(000)	376	540
Diffractometer	Bruker X8 Prospector KAPPA CCD	Bruker X8 APEX II KAPPA CCD
Radiation (λ, Å)	CuKα (1.54178)	MoKα (0.71073)
Resolution (Å)	0.83	0.83
Temperature (K)	296(2)	296(2)
$2\theta_{max}$	136.8	50.6
Completeness (%)	99.6	99.7
Reflns collected/ unique/ > $2\sigma(I)$	6974 / 3010 / 2417	20477 / 8319 / 3229
R_{int}	0.0522	0.0943
Data / parameters	3010 / 227	8319 / 595
Goodness on fit	1.100	0.921
R_1, [a] $wR2$ [b][$I > 2\sigma(I)$]	0.0558, 0.1403	0.0584, 0.0827
R_1, wR_2 [all data]	0.0672, 0.1535	0.2047, 0.1200
Flack parameter (x)	0.040(19)	0.026(12)
Δρ (e Å^{-3})	-0.39, 0.21	-0.28, 0.28

[a] $R = \sum ||F_o| - |F_c|| / \sum |F_o|$; [b] $wR = \sum \{w(F_o^2 - F_c^2)^2 / \sum w(F_o^2)^2\}^{1/2}$.
